# The Complete Genome of *Propionibacterium freudenreichii* CIRM-BIA1^T^, a Hardy Actinobacterium with Food and Probiotic Applications

**DOI:** 10.1371/journal.pone.0011748

**Published:** 2010-07-23

**Authors:** Hélène Falentin, Stéphanie-Marie Deutsch, Gwenaël Jan, Valentin Loux, Anne Thierry, Sandrine Parayre, Marie-Bernadette Maillard, Julien Dherbécourt, Fabien J. Cousin, Julien Jardin, Patricia Siguier, Arnaud Couloux, Valérie Barbe, Benoit Vacherie, Patrick Wincker, Jean-François Gibrat, Claude Gaillardin, Sylvie Lortal

**Affiliations:** 1 INRA, UMR 1253, Science et Technologie du Lait et de l′Œuf, Rennes, France; 2 AGROCAMPUS OUEST, UMR1253, Science et Technologie du Lait et de l′Œuf, Rennes, France; 3 INRA, UR1077, Unité Mathématique, Informatique et Génome, Jouy-en-Josas, France; 4 CNRS-UMR5100, Laboratoire de Microbiologie et Génétique Moléculaires, Campus Université Toulouse III, Toulouse, France; 5 Génoscope (CEA), UMR8030, CNRS and Université d'Evry, Evry, France; 6 AgroParisTech, CNRS UMR2585, INRA UMR1238, Microbiologie et Génétique Moléculaire, Thiverval-Grignon, France; University of Hyderabad, India

## Abstract

**Background:**

*Propionibacterium freudenreichii* is essential as a ripening culture in Swiss-type cheeses and is also considered for its probiotic use [1]. This species exhibits slow growth, low nutritional requirements, and hardiness in many habitats. It belongs to the taxonomic group of dairy propionibacteria, in contrast to the cutaneous species *P. acnes*. The genome of the type strain, *P. freudenreichii* subsp. *shermanii* CIRM-BIA1 (CIP 103027^T^), was sequenced with an 11-fold coverage.

**Methodology/Principal Findings:**

The circular chromosome of 2.7 Mb of the CIRM-BIA1 strain has a GC-content of 67% and contains 22 different insertion sequences (3.5% of the genome in base pairs). Using a proteomic approach, 490 of the 2439 predicted proteins were confirmed. The annotation revealed the genetic basis for the hardiness of *P. freudenreichii*, as the bacterium possesses a complete enzymatic arsenal for *de novo* biosynthesis of aminoacids and vitamins (except panthotenate and biotin) as well as sequences involved in metabolism of various carbon sources, immunity against phages, duplicated chaperone genes and, interestingly, genes involved in the management of polyphosphate, glycogen and trehalose storage. The complete biosynthesis pathway for a bifidogenic compound is described, as well as a high number of surface proteins involved in interactions with the host and present in other probiotic bacteria. By comparative genomics, no pathogenicity factors found in *P. acnes* or in other pathogenic microbial species were identified in *P. freudenreichii,* which is consistent with the Generally Recognized As Safe and Qualified Presumption of Safety status of *P. freudenreichii.* Various pathways for formation of cheese flavor compounds were identified: the Wood-Werkman cycle for propionic acid formation, amino acid degradation pathways resulting in the formation of volatile branched chain fatty acids, and esterases involved in the formation of free fatty acids and esters.

**Conclusions/Significance:**

With the exception of its ability to degrade lactose, *P. freudenreichii* seems poorly adapted to dairy niches. This genome annotation opens up new prospects for the understanding of the *P. freudenreichii* probiotic activity.

## Introduction

The genus *Propionibacterium* belongs to the class of high GC actinobacteria. All species of this genus produce propionic acid as a major metabolic end-product. However, taxonomical studies clearly distinguished two groups of propionibacteria: cutaneous and dairy. Over a century ago, *P. freudenreichii* was the first dairy species isolated from Emmental cheese [2]. Since then, other dairy propionibacteria species such as *P. acidipropionici*, and *P. thoenii* have been found in milk and cheese, and sometimes also in various biotopes like silage, soil, rumen, and waste water [3]. These observations indicate the ability to adapt to various environmental conditions. Dairy propionibacteria grow slowly (generation time ∼5 h under optimal conditions) and have low nutritional requirements.

Due to its long documented use in cheese, and in particular in Swiss type cheeses, *P. freudenreichii* has received the American Generally Recognized as Safe (GRAS) status. It has also been granted the European Qualified Presumption of Safety (QPS) status. During ripening, this species drives the fermentation of lactate into propionate, acetate and CO_2_, *via* the Wood-Werkman cycle, resulting in the formation of the characteristic “eyes” and flavor of these cheeses. *P. freudenreichii* also plays an essential role in the production of other flavor compounds, such as free fatty acids released *via* lipolysis of milk glycerides, and branched-chain acids resulting from the catabolism of amino acids [4], [5]. Because of their impact on flavor, dairy propionibacteria are now used in an increasing number of cheeses without eyes [6]. Meanwhile, a second application of *P. freudenreichii* concerns production of vitamin B12, one of nature's most structurally complex small molecules. Most of the vitamin B12 pathway has been elucidated [7], [8], [9], [10], and overproducing modified strains developed.

Finally, there is increasing interest in the probiotic activity of *P. freudenreichii*, as the bifidogenic compound it produces, 1,4-dihydroxy-2-naphthoic acid (DHNA) [11], [12], stimulates growth of bifidobacteria, which has been shown to be beneficial for human health [13], [14]. Some strains of *P. freudenreichii* adapt very well to gastric and bile salt stresses [15], [16] and are able to survive and maintain active metabolism *in vivo* in the rat or human gut [17], [18], [19]. Supernatants or live freeze-dried strains of propionibacteria are already commercially available as tablets to improve intestinal transit. *In vitro*, *P. freudenreichii* produces beneficial metabolites, including short chain fatty acids, and conjugated linoleic acid; some strains like *P. freudenreichii* JS also exhibit immunomodulatory activity [20].

The only completely sequenced, fully annotated and publicly available genome within the *Propionibacterium* genus is that of the commensal cutaneous species *P. acnes* [21].

The physiology and technological properties of *P. freudenreichii* subsp. *shermanii* CIRM-BIA1^T^ have been studied by several teams around the world. Genome sequencing will provide the molecular basis of traits important for cheese or probiotic applications, and will allow a better assessment of the “distance” between the two main groups of propionibacteria: dairy and cutaneous.

## Results and Discussion

### General genome features

**Figure 1:**
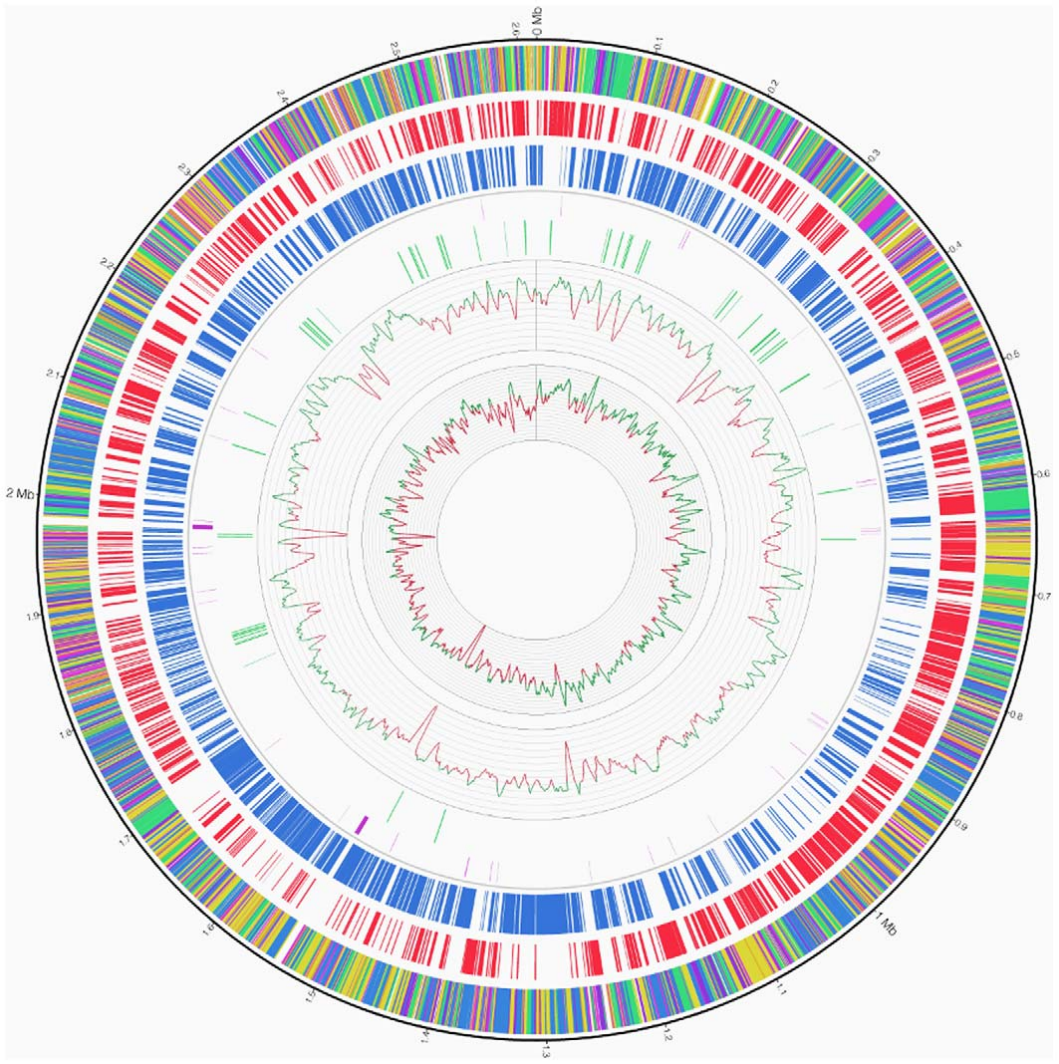
Circular representation of the genome of *P. freudenreichii* strain CIRM-BIA1^T^. The outer circle shows the coding sequence by functional category: cell envelope in green, metabolism in blue, replication, transcription and traduction in yellow, other functions in orange, hypothetical proteins in violet. The second circle shows red blocks for coding sequences on the (+) strand. The third circle shows blue blocks for coding sequences on the (−) strand. The fourth circle shows rRNA in purple and tRNA in light purple. The fifth circle shows the insertion sequences and phage integrase in green. The sixth circle shows the precentage GC (window size 10,000 bp, step size 2000 bp) ranging from 59 to 72% with a step of 1%. The seventh circle shows the GC skew (window size 10,000 bp, step size 2000 bp) ranging from −0.13 to 0.09 with a step of 0.01%. The replication origin is between 10,426 bp and 12,300 bp.

The genome of *P. freudenreichii* CIRM-BIA1^T^ consists of a circular chromosome of 2,616,384 base pairs (bp) with 67% GC content. The genome contains 2 rRNA operons and 45 tRNAs (Fig. 1). These small numbers are in agreement with the slow growth of this bacterium. Indeed, bacteria with few ribosomal operons tend to be slow-growing organisms that can utilize resources efficiently, and are capable of growth in low-nutrient environments [22]. The chromosome is predicted to contain 2439 protein-coding genes, 490 of which were identified by proteomics (2D gels and 2D-LC), followed by liquid chromatography and mass spectrometry (Table S1). These proteins correspond to 16% of the total number of predicted proteins, and 29% of the 1391 proteins predicted in the pI 4–7 and 15–150 kDa ranges. The CIRM-BIA1^T^ strain harbors no plasmid, whereas one or two plasmids, generally cryptic, have been reported in 10–30% of *P. freudenreichii* strains [23].

### Mobile elements and CRISPR

Insertion sequences and transposable elements may promote genome plasticity and contribute to bacterial adaptability [24]. Seventy-two different transposases were found in the *P. freudenreichii* genome and each of them is repeated one to eight times. The genome also contains 22 different Insertion Sequences (IS) belonging to four different families (IS3, ISL3, IS30 and IS481) according to the nomenclature at http://www-is.biotoul.fr (Table S2, [25]). IS represent 3.47% (in base pairs) of the genome, with 2.95% of complete IS and 0.52% of partial IS. Each complete IS is repeated one to ten times. IS are located in the first and last quarters of the genome (near the replication origin).

Six of the 22 IS are repeated twice and are colocalized with two integrases between the 1,242,179 bp and 1,287,760 bp positions, which could be potentially a highly variable region. This region does not contain other genes encoding proteins of other functions.

The transposase gene is mutated in ISPfr8: a guanidine insertion created a frameshift observed in all three copies rendering it non-functional. IS Aar30 was also found in *Arthrobacter arilaitensis,* a bacterium of smear-ripened cheese surfaces. In only two cases does the IS contain functional genes (different from transposases): (1) IS*Pfr*21 contains a transposase gene and two other genes, one coding for PFREUD_08140, a transcription factor belonging to the TetR regulator family, and the other coding for PFREUD_08150, a protein of unknown function similar (42%) to a protein of *Sinorhizobium* sp.; (2) a mobile insertion cassette (MIC) with the same termini as ISPfr11 but without the transposase gene is located between 371,214 and 372,936 bp. This MIC contains a gene encoding a protein of unknown function, PFREUD_03020, similar (52%) to a protein of *Kineococcus radiotolerans*. In most cases, the IS insertion has no obvious impact because very few genes are present.

**Figure 2:**
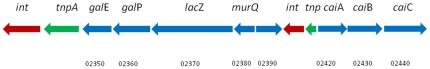
In *P. freudenreichii* strain CIRM-BIA1^T^, genes involved in lactose metabolism are bordered by a transposase and integrase. The lactose locus consists of three genes, PFREUD_02370, PFREUD_02360 and PFREUD_02350 (numbers are indicated below the arrows), which encode a βgalactosidase, LacZ, a galactoside transporter, and an UDP-glucose isomerase, GalE, respectively. *Lac* genes may have been acquired through a horizontal transfer event. PFREUD_02380 encodes the N-terminal end of an N-acetylmuramic acid 6-phosphate etherase and PFREUD_02390 encodes a transcriptional regulator.

However, the transposable elements of *P. freudenreichii* can induce phenotypic changes. The first example is provided by an IS upstream the *gtf* gene, PFREUD_19370. The *gtf* gene encodes a polysaccharide synthase involved in the synthesis of capsular exopolysaccharides (EPS). While the presence of capsular EPS in *P. freudenreichii* is strain-specific, all strains tested were shown to possess one *gtf* copy. Recently, we demonstrated that the presence of an IS element within the putative promoter region of *gtf* enhanced *gtf* transcription, and was associated to the capsular phenotype [26], [27]. The second example concerns the ability to ferment lactose. *P. freudenreichii* CIRM-BIA1^T^ is able to degrade lactose but this trait is strain-dependent. In the genome, the lactose locus consists of three genes, PFREUD_02370, PFREUD_02360 and PFREUD_02350, encoding a β-galactosidase, LacZ, a galactoside transporter, GalP, and an UDP-glucose isomerase, GalE1, respectively. These three genes are surrounded by integrases (PFREUD_24460) and transposases (PFREUD_24470) (Fig. 2). GalE is similar (73% to Q6AGJ6 from *Leifsonia xylii*) to GalE of other actinobacteria, but LacZ and GalP are similar to those of several species of *Mannheimia succiniciproducens* and of *Clostridium* (LacZ, 55% similar to Q65UK4 from *Mannheimia succiniciproducens*; GalP, 52% similar to Q2WUG8 from *Clostridium beijerincki*). These two species, like propionibacteria, are present in the cow rumen ecosystem. These observations strongly suggest that the Lac genes may have been acquired through a horizontal transfer event mediated by phage infection, which could explain why this ability is strain dependent. As lactose fermentation is one of the two phenotypic criteria used to distinguish the subspecies *P. freudenreichii* subsp. *shermanii* (positive for lactose fermentation) from *P. freudenreichii* subsp. *freudenreichii* (negative for lactose fermentation), the justification of these subspecies should be further investigated. Since no difference in GC content was observed in these regions relative to the rest of the genome, this locus may have been acquired a long time ago.

The phages in the genome could not be precisely located because the *attP* and *attB* sites are unknown. One temperate phage closely related to the L5 [28] and FRAT1 [29] temperate mycobacteriophages was identified. This locus contains a gene coding for a capsid protein of the HK97 family (PFREUD_04110, 57% similar to Q1B5C3 from *Mycobacterium sp*.), several proteins of unknown function, and a phage integrase (PFREUD_04210) with 45% similarity with the integrase of mycobacteriophage L5 (Swissprot entry VINT_BPML5).

Several traces of prophages were also identified in the genome. They are evidenced by the presence of 31 integrase loci, scattered throughout the chromosome, but with most (22/31) clustered less than 100 kb from each other. Six regions of the chromosome have clusters of integrases: (1) between 124,000 and 129,000 bp, two copies of the PFREUD_24770; (2) from 233,000 to 291,000 bp, three integrases PFREUD_24990, PFREUD_24980 and PFREUD_24460 at the lactose locus; (3) from 427,000 to 509,000, three integrases (PFREUD_24610, PFREUD_24630 and PFREUD_04210); (4) from 1,825,318 to 1,836,629 bp, PFREUD_24770 and PFREUD_24830; (5) from 2,288,000 to 2,295,000 bp PFREUD_24990, PFREUD_24980; and, lastly, (6) from 2,432,000 to 2,482,000, PFREUD_21190, PFREUD_22610 to PFREUD_22650, PFREUD_25160, and PFREUD_25070. These regions also have a lower GC content (Fig. 1). This illustrates the highly variable regions in the genome.

Phage attacks are frequently reported during cheese manufacture using lactic acid bacteria, but not for dairy propionibacteria. However, lysogeny was fully demonstrated for *P. freudenreichii* ITG P18 and the presence of prophage was detected using hybridization experiments with phi101 in many species, including CIRM BIA1 [30]. Clustered regularly interspaced short palindromic repeats (CRISPR) provide acquired resistance to viruses in prokaryotes, as recently demonstrated in *Streptococcus thermophilus* [31]. CRISPR loci typically consist of several non-contiguous direct repeats separated by variable sequences called spacers and are often adjacent to *cas* (CRISPR associated) genes. Three CRISPR loci were identified in the *P. freudenreichii* genome using the CRISPR finder tool (crispr.u-psud.fr/Server/CRISPRfinder.php, [32]). *In silico* analyses of the spacers showed sequence similarities with foreign elements, including bacteriophages sequences. The CRISPR1 locus, located between 441,264 and 443,689 bp, harbors a 36 bp direct repeat (DR) sequence GCCTCAATGAAGGGCCCCTCCAGAAGGAGGGGCAAT and 34 spacer sequences of 35 to 40 bp. Blast results on the EMBL phage database (http://blast.ncbi.nlm.nih.gov/Blast.cgi) showed that the third spacer displays a strong similarity (32/39 nt identity) to the *Stenotrophomonas* phage S1 (EU849489), and that the 26^th^ and 27^th^ spacers shared a high level of identity with orf6 and orf9 of the *Propionibacterium* phage phiB5, a filamentous ssDNA inovirus [33]. Two other possible CRISPR loci were identified, however they did not contain two identical DRs. The first locus, located between 652,209 and 652,301 bp, contains the 23 bp DR sequence CAAGCGCCCTGCTGTGTTCGTTT and only one spacer, without homology. The second one, located between 2,215,874 and 2,216,038, contains a 24 bp DR sequence CTTCTTCGCCGCCGGCTTCTTGGC and three spacer sequences without homology in the database. The PFREUD_03690 gene for cas1/cas4 associated CRISPR proteins was found near the replication origin between 399 and 2000 bp but no CRISPR was associated with it. The presence of CRISPR loci indicates that *P. freudenreichii* has been in contact with phages, and strongly suggests that they may contribute to its resistance to phage attacks.

In conclusion, IS and prophages are a source of plasticity and adaptability of the bacteria to various environments, and the influence of transposable elements on two phenotypic traits in *P. freudenreichii* was illustrated by the formation of a capsular EPS and the ability to ferment lactose. The presence of the CRISPR loci may protect *P. freudenreichii* against phage attacks.

### Comparative genomics with *P. acnes*

**Figure 3:**
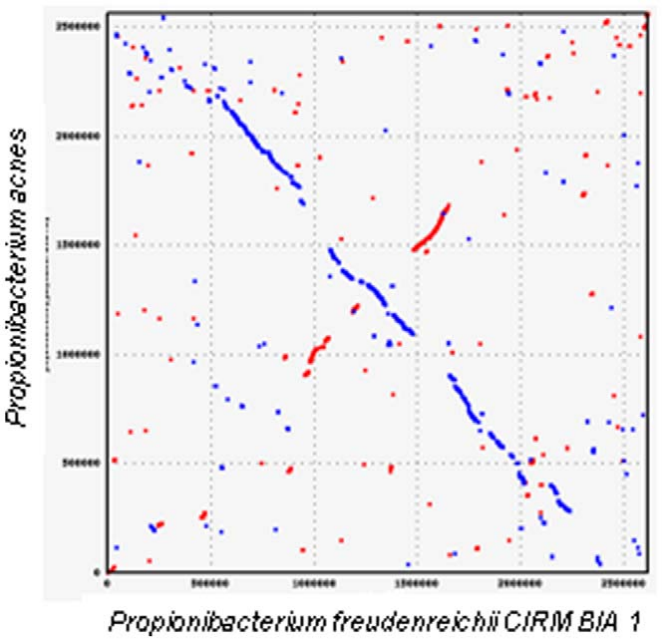
Homology dot plot comparing *P. freudenreichii* strain CIRM-BIA1^T^ and *P. acnes* at the protein level. *dnaA* is at the zero position. Forward matches are displayed in red and reverse matches are displayed in blue. Relatively high synteny along the genome is observed, with the exception of two inversions between 90,000–120,000 bp and 100,000–115,000 bp.

Belonging to the cutaneous group, *P. acnes* is the only species within the *Propionibacterium* genus with a complete genome sequence publicly available. A comparison of this species with *P. freudenreichii* CIRM-BIA1^T^ was carried out. *P. acnes* has a 2.5 Mb and 60% GC genome composed of 2333 putative genes [21]. The two species differ by their habitat and ecology. *P. acnes* is a major inhabitant of human skin and is considered to be an opportunistic pathogen, while *P. freudenreichii* has GRAS status. The two species, however, share some similarities. Both species can grow under microaerophilic and anaerobic conditions, and produce propionic acid as the main fermentation product. To compare gene colinearity, we aligned the genomes at the protein level because the nucleotide sequences were too divergent (Fig. 3). A relatively high synteny between the genomes was observed, with the exception of two inversions between 90,000–120,000 bp and 100,000–115,000 bp. We also mined the *P. freudenreichii* genome to look for the genes potentially involved in pathogenicity described in the *P. acnes* genome. In performing bidirectional best hits (Table S3), no similarity was found between the *P. freudenreichii* predicted protein set, and *P. acnes* proteins putatively involved in the degradation of host molecules or in mediating inflammation. In particular, the genes encoding endoglycoceramidase, sialidase, hemolysin, CMP factor, and toxins in *P. acnes* were not identified in *P. freudenreichii*. However, two genes annotated in *P. acnes* as encoding invasion-associated proteins (PPA_1962 and PPA_0721) were found in *P. freudenreichii* (three-quarters of the C-terminal part of PFREUD_04850 is 50% similar to Q6A6D4; PFREUD_04280 is 73% similar to Q6A9T8 from *P. acnes*). These proteins were annotated in *P. freudenreichii* as “cell-wall peptidase, NlpC/P60 family secreted protein”, due to their high similarity (73% and 76%) to *S. coelicolor* Q9KY71_STRCO and Q9KY68_STRCO, respectively, and the presence of a signal peptide and Pfam 00877 domain.

We also compared the putative esterases/lipases in *P. freudenreichii* and *P. acnes* because lipolytic activity is an important feature in both species. *P. freudenreichii* has a major role in the hydrolysis of milk glycerides during cheese ripening [5]. The free fatty acids resulting from lipolysis are important compounds in cheese flavor. *P. acnes* lipases are thought to degrade human skin lipids such as sebum. Free fatty acids resulting from this activity could promote bacterial adherence and colonization of the sebaceous follicle [21]. Comparison of esterases/lipases in *P. acnes* and *P. freudenreichii* showed that the two species shared a common pool of intracellular esterases, but differed in secreted esterases/lipases. *P. acnes* produces a secreted lipase, GehA, and another lipase with 41% identity to GehA at the protein level, encoded by the genes PPA2105 and PPA1796, respectively [21], [34]. Neither of these proteins has an ortholog in *P. freudenreichii. P. freudenreichii* was recently shown to produce a secreted lipase active on milk fat, encoded by PFREUD_04340 [35]. However, this enzyme has no ortholog in *P. acnes.* Of the 11 putative esterases identified in the *P. freudenreichii* genome, six predicted cytoplasmic proteins and one potentially surfaced exposed protein [35], [36], have orthologs in *P. acnes.*

In conclusion, no pathogenicity factor from *P. acnes* was identified in *P. freudenreichii*. This finding is consistent with the GRAS and QPS status of *P. freudenreichii.*

### Reconstruction of specific metabolic pathways

#### Carbon substrates and nutritional requirements

Experimentally, *P. freudenreichii* is able to grow, under anaerobic conditions, in a minimal medium containing a carbon source, ammonium as the sole nitrogen source, minerals, and two to four vitamins. Like most other *P. freudenreichii* strains, CIRM-BIA1^T^ is able to use a variety of carbon substrates, including sugars (lactose, galactose, D-glucose, D-mannose), alcohols (erythritol, glycerol, adonitol), and acids (lactic acid, gluconic acid) [37] (Table S4). Genome annotation clearly confirmed that this strain was able to import these carbon sources and to catabolise them by different pathways (glycolysis, pentose phosphate, and Entner-Doudoroff pathways). The use of other carbon substrates, such as D-fructose, L-arabinose, ribose, D-raffinose, saccharose, xylitol, and gluconic acid, is strain-dependent in *P. freudenreichii* (Table S4). For example, the CIRM-BIA1^T^ strain does not catabolise L-arabinose, in contrast to most *P.freudenreichii* strains. The *ara*B gene (PFREUD_06570 and PFREUD_06580) encoding a ribulokinase is a pseudogene in the CIRM-BIA1^T^ strain. At least one other *ara*B gene is present at another locus of the genome (PFREUD_22370) and may complement the pseudogene. However, no transporter for arabinose was found, which may explain why CIRM-BIA1^T^ does not catabolize L-arabinose. The ability to use propanediol, a less common growth substrate, was reported for *P. freudenreichii* subsp. *freudenreichii* ATCC 6207 [38]. CIRM-BIA1^T^ possesses a complete pdu (propanediol utilization) operon (from PFREUD_08980 to PFREUD_09150), with predicted proteins similar to those of *Salmonella enterica* serovar Typhimurium [39], [40]. The gene order is not conserved in the two species; however, as in *Salmonella*, an integrase-coding gene was identified at the end of the locus, suggesting that this operon could have been acquired through horizontal transfer.

Regarding nitrogen requirements, *P. freudenreichii* is prototrophic for all amino acids and nucleotides. The complete biosynthetic pathways of all amino acids were reconstituted (to examine the complete pathways with the annotated genes, see http://www.genome.jp/kegg/catalog/org_list.html).

**Figure 4:**
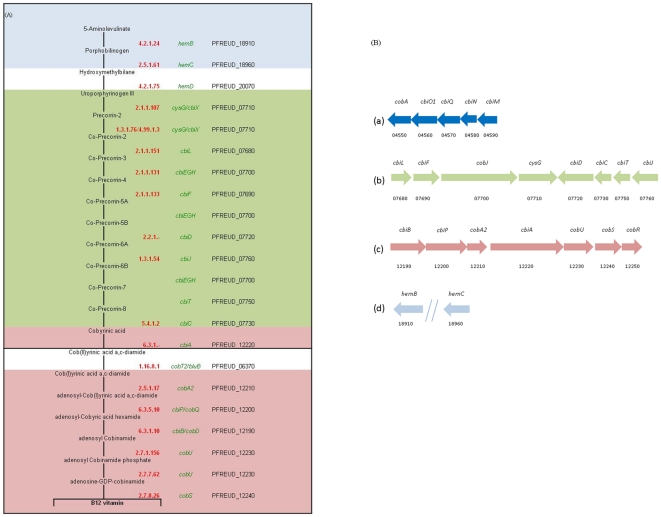
Vitamin B12 biosynthesis in *P. freudenreichii* strain CIRM-BIA1^T^. (A) Vitamin B12 pathway: enzyme number (red), gene name (green) and locus tag (black). (B) Four loci (a, b, c, d) are involved in vitamin B12 biosynthesis. Gene names are indicated above the arrows, locus tags (PFREUD) are indicated below the arrows. The locus (a) codes for the cobalt ABC transporter. The colors of the arrows used in (B) for locus b, c and d are also used for the pathway background in (A), with the exeption of the steps in the white background corresponding to isolated genes (*hemD* and *cobT2/bluB*).

Regarding vitamins, all propionibacteria strains require pantothenate (vitamin B5) and biotin (vitamin H). Some strains require thiamine (B1) and *p*-aminobenzoic acid in addition [3]. Genome data showed that all the vitamin synthesis pathways are complete in strain CIRM-BIA1^T^, with the exception of pantothenate and biotin. In our study, it was shown (see Material & Methods and Table S5) that pantothenate and biotin were indeed required for CIRM-BIA1^T^ growth, in agreement with genomic data. Unexpectedly, we also observed that *P. freudenreichii* CIRM-BIA1^T^ required thiamine whereas the biosynthetic pathway seemed complete. The vitamin B12 synthesis pathway (anaerobic early cobalt incorporation) of one strain of *P. freudenreichii* was previously described in detail [8]–[10]. Fourteen genes were cloned in *E. coli* and were sequenced. The organization of genes for vitamin B12 synthesis in CIRM-BIA1^T^ is in agreement with the results reported for this strain (Fig. 4). However, five new genes (*cbiA*, *cobU*, *cobS*, *cobR* at locus (c) and *hemD*) were discovered and revealed another locus putatively responsible for cobalt transport (PFREUD_20190 and 20200). The complete description of vitamin B12 synthesis pathway in *P. freudenreichii* opens up new prospects for genetic engineering and for the screening of highly productive strains using molecular tools.

#### Wood-Werkman cycle and pyruvate conversion

**Figure 5:**
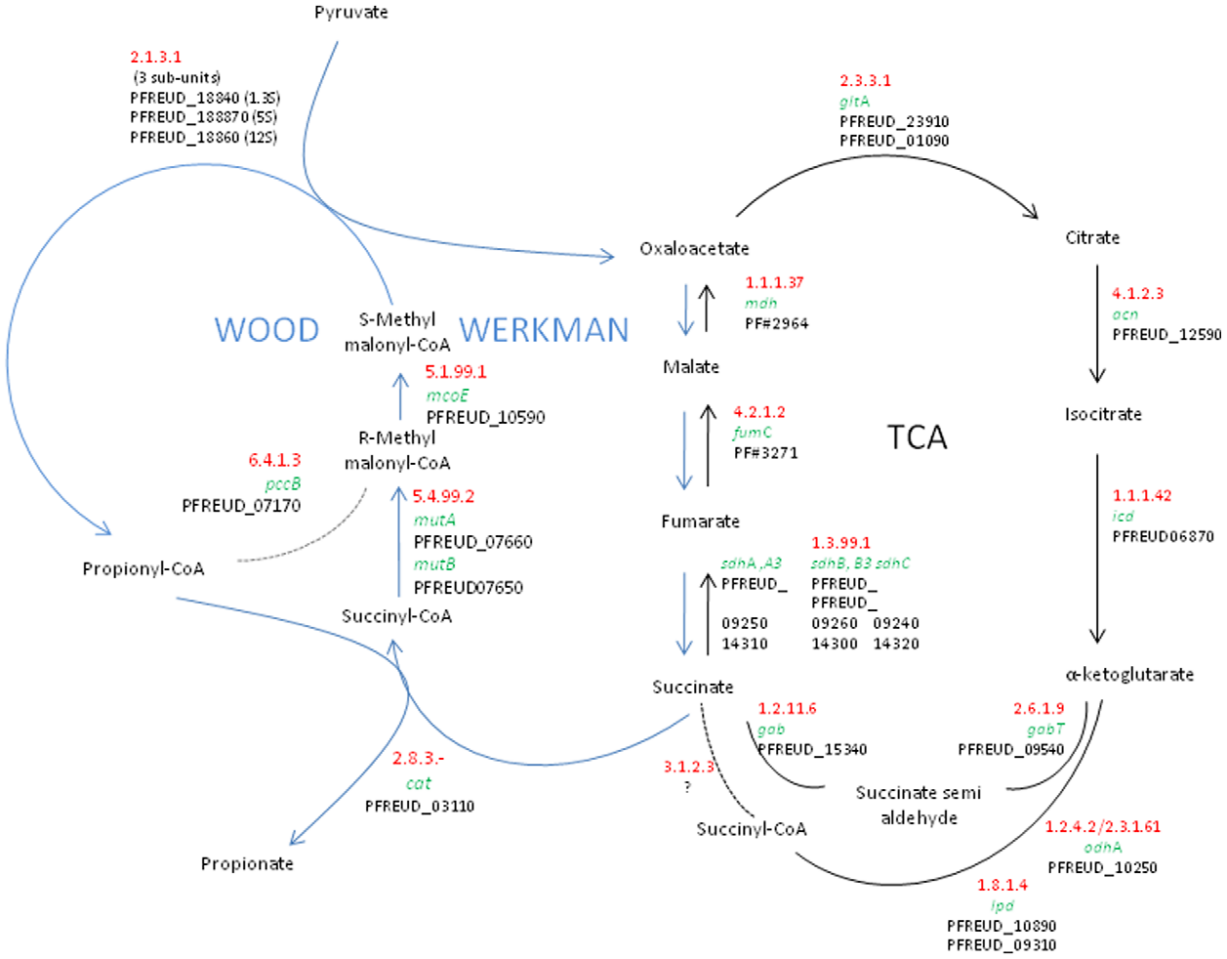
Schematic representation of the Wood-Werkman cycle (in blue) and tricarboxylic acid (TCA) cycle (in black) in *P. freudenreichii* strain CIRM-BIA1^T^. Enzyme numbers are in red, gene names are in green, and locus tags are in black. Reactions are directed toward propionate production, but are reversible.

Propionic acid is the major end product of fermentation in propionibacteria and confers their typical flavor to Swiss-type cheeses. Two pathways of propionate formation have been described in bacteria. The first pathway, known as Wood-Werkman cycle, involves succinyl-CoA and methylmalonyl-CoA as intermediates. It was first described in *P. freudenreichii* and *Pelobacter propionicus* [41], and is present in other bacterial species such as *Bacteroïdes fragilis* [42], *Veillonella parvula* [43], and *Veillonella gazogenes* [44]. The second pathway involves an acrylyl-CoA intermediate and has been described in *Clostridium propionicum* [45]. The Wood-Werkman cycle was extensively investigated in *P. freudenreichii* at biochemical [46]–[48] and genetic levels. It includes a methylmalonyl-CoA carboxytransferase, a methylmalonyl-CoA epimerase, and a methylmalonyl-CoA mutase. The key feature of the Wood-Werkman cycle in *P. freudenreichii* is a transcarboxylation reaction without the involvement of free CO_2_. The enzyme catalyzing this reaction is a methylmalonyl-CoA carboxytransferase, transferring a carboxyl group from methylmalonyl-CoA to pyruvate to form oxaloacetate and propionyl-CoA (Fig. 5). The enzyme involved has been fully characterized and its structure resolved. It is a biotin-dependent carboxytransferase (EC 2.1.3.1) composed of three subunits. The methylmalonyl-CoA carboxytransferase is encoded by a polycistronic gene containing four coding sequences [17]. In CIRM-BIA1^T^, the 1.3S and the 5S subunits, encoded by PFREUD_18840 and PFREUD_18870, have the same length and share 76% and 99% similarity with Swissprot entries P02904 and Q70AC7 from *P. freudenreichii* subsp. *shermanii* W52, respectively. The 12S subunit, encoded by PFREUD_18860, shares 96% similarity with the N-terminal part (524 amino acids) of the Swiss-Prot entry Q8GBW6, and would be a functional but truncated protein, as in the *P. acnes* genome [21]. Another coding sequence (PFREUD_18850) encodes the C-terminal part (98% similarity with the Swiss-Prot entry Q8GBW6) of the 12S subunit. The 12S subunit coding sequence is in agreement with those of *P. acnes* but do not agree with the crystallographic data of the 12S subunit in *P. freudenreichii* [49]. The 12S subunit coding sequence is probably strain-dependant without effect on the enzyme functionality because the catalytic domains are all located in the N-terminal part of the protein.

The complete Wood-Werkman cycle could be reconstituted (Fig. 5), as well as the pathway for oxidative decarboxylation of pyruvate, which results in the formation of acetate and CO_2_. A partial TCA cycle was also reconstituted, but lacked a succinyl-CoA hydrolase (E.C.3.1.2.3) (Fig. 5). This activity was previously described in *P. freudenreichii* subsp. *freudenreichii* CIP103026 [47]. In the CIRM-BIA1^T^ strain, succinyl-CoA formation results from CoA transferase activity.

The co-metabolism of aspartate/asparagine and lactate was previously reported [50]. During lactate fermentation, aspartate is deaminated to fumarate by an aspartate ammonia lyase ( =  aspartase) encoded by *aspA* (PFREUD_16320 and PFREUD_16330), fumarate is then converted to succinate, with regeneration of oxidized co-enzymes and ATP. The intensity of this conversion depends on the conditions and on the *P. freudenreichii* strain [50], [51]. Interestingly, cells using this pathway modulate the conversion of pyruvate towards oxidative decarboxylation to maintain their oxido-reduction balance, leading to more acetate and CO_2_ at the expense of pyruvate, which is reduced to propionate *via* the Wood-Werkman cycle. In addition to this aspartate catabolism pathway, other genes encoding aspartate-converting enzymes were found in the CIRM-BIA1^T^ genome: aminotransferases (*asp*B encoded by PFREUD_04930, *tyr*B encoded by PFREUD_09460, *asp*C encoded by PFREUD_23330), L-aspartate oxidase, (EC 1.4.6.16, encoded by *nad*B (PFREUD_09210 and PFREUD_21690)) and adenylosuccinate synthetase (EC 6.3.4.4, encoded by PFREUD_19280). Each enzymatic activity can impact intracellular aspartate concentration and can modulate CO_2_ production, consequently affecting the size of the eyes that form in cheese.

#### Respiration

Although *P. freudenreichii* is usually grown under anaerobic or microaerophilic conditions, it possesses all the genes required for aerobic respiration: genes encoding NADH dehydrogenase (EC 1.6.5.3/1.6.99.5, *nuo*A, *nuo*B, *nuo*C, *nuo*D, *nuo*E, *nuo*F, *nuo*G, *nuo*H, *nuo*I, *nuo*J, *nuo*K, *nuo*L, *nuo*M, *nuo*N), succinate dehydrogenase (EC 1.3.99.1, *sdh*A, *sdh*A3, *sdh*B, *sdh*B3 and two *sdh*C), cytochrome bd complex (*cyd*A and *cyd*B), ATPase (*atp*A, *atp*B, *atp*C, *atp*D, *atp*E, *atp*F, *atp*G, *atp*H) and the complete pathway for heme synthesis (*hem* genes). Under anaerobic conditions, the electron acceptor in *P. freudenreichii* can be sulfate, fumarate, menaquinone (vitamin K2), or the pool of ferrous iron and humic acid in the soil [52]. Nitrate cannot be used as an electron acceptor, because CIRM-BIA1^T^ has no denitrification capacity. In fact, the gene corresponding to the beta subunit of nitrate reductase is a pseudogene due to a frameshift. The ability to reduce nitrate is the second criterion used to distinguish the subspecies *shermanii* (negative) from *freudenreichii* (positive).

### High storage ability and long survival

*P. freudenreichii* displays numerous features which allow its long-term survival, including the accumulation of energy and carbon storage compounds, the accumulation of compatible solutes, and the induction of a multi-tolerance response under carbon starvation.

Interestingly, *P. freudenreichii* is able to accumulate inorganic polyphosphate (polyP) as an energy reserve whereas most bacteria utilize ATP. Only bacteria particularly adapted to extreme environments are able to use polyP [53]. *P. freudenreichii* strain ST33 grown on lactate accumulates large amounts of long-chain polyP, up to 3% of the cell dry weight [54], whereas ST33 accumulates short chain polyP and exhibits a 100-fold decrease in the amount of long chain polyP when grown on glucose. The key enzyme involved in the synthesis of polyP in bacteria is polyphosphate kinase (PPK). PPK synthesizes polyP by transferring the terminal phosphate of ATP to polyP. In many species, *ppk* mutants are unable to survive during stationary phase [55]. In *P. freudenreichii* CIRM-BIA1^T^, the *ppk* gene is followed by genes coding for NUDIX (a kind of pyrophosphohydrolase) and for an ABC phosphate transporter. Several polyP or pyrophosphate using enzymes were found in the genome (Table S6). We detected nine different NUDIX hydrolases (PFREUD_02720, PFREUD_07140, PFREUD_09230, PFREUD_09830, PFREUD_10620, PFREUD_17200, PFREUD_18300, PFREUD_19450, PFREUD_19940). In eukaryotes and prokaryotes, the number of NUDIX genes varies from 0 to 30. A high number of NUDIX genes reflects the metabolic complexity and adaptability of the organism [56]. Utilization of pyrophosphate instead of ATP also adds metabolic flexibility, because reactions become reversible. For example, pyrophosphate phosphofructokinase (PFREUD_12040) is involved both in glycolysis and in gluconeogenesis [57].

*P. freudenreichii* CIRM-BIA1^T^ is able to synthesize glycogen, as reported for the first time using *in vivo* ^13^C NMR analysis of cells grown in the presence of ^13^C glucose [58]. The genes potentially involved in glycogen metabolism had not been previously described in *P. freudenreichii*. Six genes related to glycogen metabolism were identified in the genome: PFREUD_16180, PFREUD_16190, PFREUD_10630, PFREUD_10670, PFREUD_10700, and PFREUD_22680, encoding ADP-glucose pyrophosphorylase, a glycogen synthase, a glycogen branching enzyme, an α-glucan phosphorylase, and two glycogen debranching enzymes, respectively. Four of these six genes were also found in *P. acnes*. Since phenotypic data indicate that neither *P. freudenreichii* nor *P. acnes* is able to ferment extracellular glycogen, these enzymes must be involved in intracellular glycogen accumulation and/or hydrolysis.

*P. freudenreichii* strains, including CIRM-BIA1^T^, are able to synthesize and accumulate trehalose from glucose and pyruvate [59]. The synthesis of trehalose is enhanced at the beginning of the stationary phase and under oxidative, osmotic, and acid stress conditions [59], [60]. This ability is strain-dependent [60]. Trehalose is most commonly synthesised in bacteria *via* the trehalose-6-phosphate synthase/phosphatase (OtsA–OtsB) pathway and catabolised by trehalose synthase (TreS). The genes *otsA, otsB,* and *treS* were previously identified in strain NIZO B365 [61]. These three genes (PFREUD_12170, PFREUD_12160, PFREUD_10650) were similarly organized in CIRM-BIA1^T^ and the corresponding proteins showed 99%, 99% and 100% similarity, respectively, to the previously reported sequences.

*P. freudenreichii* is also known to accumulate glycine betaine. In addition to osmotic stress adaptation [62], glycine betaine participates in long-term survival, as does trehalose, by acting as a chemical chaperone. Genes supporting glycine betaine transport and biosynthesis reflect this ability. Glycine betaine is synthesized by oxidation of choline (dehydrogenase, PFREUD_19130), leading to betaine aldehyde, which is then oxidized to glycine betaine (Betaine-aldehyde dehydrogenase, *dha1,* PFREUD_01860).

*P. freudenreichii* CIRM-BIA1^T^ survives for a very long time at room temperature even under conditions of carbon starvation, and is considered a non-lytic strain. Starvation and stationary phases induce a multi-tolerance response in *P. freudenreichii* [63], associated with over-expression of molecular protein chaperones [64]. A few strains of this species (about 7%) are unable to survive after one week under such conditions and lyse [65]. The polyP and glycogen accumulated in the exponential phase and trehalose accumulated at the beginning of the stationary phase are likely to be useful for cell survival in the stationary phase, or in the dormant phase induced by carbon or oxygen starvation. Dormancy has previously been reported in other actinobacteria like *Koccuria sp, Mycobacterium* sp. and *Rhodoccocus* sp [66]. Dormancy is defined as ‘a reversible state of low metabolic activity, in which cells can persist for extended periods without division’ [67]. In the *P. freudenreichii* genome, PFREUD_06100 encodes a protein similar (50%) to the Rpf (resuscitation promoting factor) protein from *Mycobacterium smegmatis* ATCC 700084. Rpf is an essential protein for the growth of dormant cells from different actinobacteria and is propably involved in long-term survival of *P. freudenreichii*.

In conclusion, several genes involved in phosphate, glycogen, and trehalose metabolism and a gene encoding an Rpf protein are good candidates to explain the survival of a majority of *P. freudenreichii* strains in long-term stationary phase conditions. The long-term survival ability is favorable for both probiotic and cheese starter applications of dairy propionibacteria.

### Probiotic potential of *Propionibacterium freudenreichii*

General stress adaptation genes, key factors of probiotic capacity, are multicopy and stress-induced in P. freudenreichii. Two copies of the *gro*SL operon (*gro*L: PFREUD_06470, PFREUD_18470; *gro*S: PFREUD_06460), the *clp*B ATP-dependent protease (PFREUD_17920, PFREUD_19250), the endopeptidase *clp*P (PFREUD_08240, PFREUD_08250), the *dna*KJ operon (dnaK: PFREUD_04630, PFREUD_17840; *dna*J: PFREUD_04650, PFREUD_17820), and *grp*E (PFREUD_04640, PFREUD_17830) and 3 copies of the surface serine protease *htr*A (PFREUD_17860, PFREUD_02310, PFREUD_02320) and the *hsp*20 chaperone (PFREUD_09500, PFREUD_22780, PFREUD_22790) were found in the genome. The redundancy and inducibility of this chaperone and protease machinery in *P. freudenreichii* [16], [68] suggests the ability to efficiently and rapidly adapt to stressful environments, such as the human host [69].

Stress-induced genes reflect the ability to cope with digestive (acid and bile) stresses. Regulation of intracellular pH is crucial for survival. Analysis of the *P. freudenreichii* genome reveals a complete *atp*BEFHAGDC operon, whose gamma chain (*atp*G, PFREUD_10480) is induced in *P. freudenreichii* by acid and bile salts [70]. These stimuli also induce pyruvate-flavodoxin oxidoreductase (*nif*J, PFREUD_01840) and succinate dehydrogenase (*sdh*B, PFREUD_14300), which are involved in electron transport and ATP synthesis, as well as glutamate decarboxylase (*gad*B, PFREUD_23230) and aspartate ammonia-lyase (*asp*A, PFREUD_16320), which are involved in intracellular pH homeostasis. Proteins involved in protection and repair of DNA are crucial for survival. Genome analysis demonstrated the presence of members of the SOS response including *lex*A, *rec*A and *uvr*ABC in *P. freudenreichii*. Moreover, the helix-destabilizing Ssb protein (PFREUD_23460), which is involved in DNA recombination and repair, as well as Dps (PFREUD_02870), which protects DNA against oxidative stress, are stress-induced in *P. freudenreichii* [68]. Stress-induced genes also reflect the ability to modulate the envelope properties. This includes the D-alanylalanine synthetase (*ddl*A, PFREUD_13250) and the UDP- MurNAc-pentapeptide synthetase (*mur*F, PFREUD_15540) [68], [71]. The *mur*F gene localizes to the same locus as the entire set of genes corresponding to peptidoglycan biosynthesis in *P. freudenreichii*, suggesting co-regulation. Stress-induced genes include the branched-chain aminoacid transferase *Ilv*E (PFREUD_13350), which is involved in the synthesis of branched-chain fatty acids that are important for stress tolerance in *S. aureus* [72]. Survival and activity within the gut depend on oxidative stress remediation, as bile was shown to induce oxidative stress [73]. *P. freudenreichii* possesses an arsenal of genes for disulfide-reduction and elimination of reactive oxygen species. The *P. freudenreichii* genome encodes a redundant thioredoxin system (10 thioredoxins: PFREUD_05410, PFREUD_19550, PFREUD_24100, PFREUD_24110, PFREUD_10600, PFREUD_07790, PFREUD_17940, PFREUD_18140, PFREUD_08110, PFREUD_00640) and two peptide-methionine-S-oxide reductases (PFREUD_17100, PFREUD_12520). Moreover, in response to bile salts, *P. freudenreichii* over-expressed the iron/manganese superoxide dismutase (*Sod*A, PFREUD_06110), catalase (*Kat*A, PFREUD_23800), Glutathione S-transferase (*Gst*, PFREUD_12610), two cysteine synthases (*Cys*2, PFREUD_16420; *Cys*1, PFREUD_06560) and S-adenosylmethionine synthetase (*Met*K, PFREUD_11410). The occurrence of a sodium/bile acid symporter, PFREUD_14830, reflects adaptation to the gut environment. Moreover, we identified four genes encoding multidrug resistance transporters (PFREUD_08180, PFREUD_17620, PFREUD_22240, PFREUD_00020) indicating an ability to cope with toxic compounds. Two genes encoding heavy metal translocating P-type ATPases (PFREUD_04920, PFREUD_22190) further suggest adaptation to toxic environments. The genome suggests a significant ability to sense changes in the environment. Eleven two-component regulatory system histidine kinases were detected (PFREUD_00320, 00810, 01640, 03260, 06720, 10230, 15210, 17760, 17900, 18790, 21970). Finally, stress-induced genes [16], [68] include polyribonucleotide nucleotidyltransferase (*pnp*A, PFREUD_14570) and the inosine-5-monophosphate dehydrogenase (*gua*B, PFREUD_06480), suggesting the ability to synthesize the alarmone ppGpp in response to stress.

The genome reveals adaptation to the nutritional environment of the gut. *P. freudenreichii* maintains an active metabolism in animal and human guts [17], [19], indicating that it can use substrates present in the colon. Accordingly, the complete pathway corresponding to gluconic acid degradation, including gluconate kinase (PFREUD_01040) and 6-phosphogluconate dehydrogenase (PFREUD_04620) was identified, in agreement with gluconate utilization. The complete *iol* operon, mostly detected in inhabitants of soil but also in *L. casei* BL23 [74], was discovered in *P. freudenreichii*, consistent with utilization of myo-inositol by this bacterium. Finally, the preferred carbon source for propionibacteria, lactic acid, is one of the end products of colic fermentation by indigenous bacteria including lactic acid bacteria and bifidobacteria.

Interaction with the host gut is strongly suggested by the presence of genes encoding key surface proteins. Analysis of the genome using SurfG+ [75] suggests the existence of 161 surface exposed proteins, including seven distinct S-layer proteins (PFREUD_03310, PFREUD_16070, PFREUD_18270, PFREUD_18290, PFREUD_23030, PFREUD_23570, PFREUD_00110), one of which, SlpA (PFREUD_18290) was identified here by surface proteomics. Slp proteins are involved in lactobacilli adhesion [76] and immunomodulation [77]. Two parts (PFREUD_23540 and PFREUD_23560) of a disrupted gene which are similar to an internalin A-like gene, involved in adhesion by other probiotic organisms, exist in the *P. freudenreichii* genome. The protein was identified by proteomics. Moreover, PFREUD_05930 exhibited 53% sequence similarity to the *Bifidobacterium bifidum* surface lipoprotein *Bop*A, which is involved in adhesion to colonocytes and immunomodulation [78]. Finally, the *gtf* gene (PFREUD_19370), responsible for the biosynthesis of a *P. freudenreichii* surface polysaccharide was discovered. A (1,3)- β-D-glucan capsule has been reported to affect immunomodulation in *Pediococcus parvulus* [79]. The involvement of *P.freudenreichii* surface proteins in adhesion [80] and immunomodulatory properties [20] should therefore be investigated.

Some genes support production of beneficial metabolites. The methylmalonyl-CoA carboxytransferase operon is induced by acid and bile salt stress and is also induced *in vivo* during transit through the digestive tract [68], [17]. This result strongly suggests that *P. freudenreichii* is able to release *in vivo* short chain fatty acids known for their beneficial effects on colon epithelial cells [81]–[83]. The bifidogenic effect of dairy propionibacteria means that they are also of major interest for their use as probiotics in the field of microbiota modulation. The compound 1,4-dihydroxy-2-naphtoic acid (DHNA) is a precursor of menaquinone (vitamin K2), and has been identified as a bifidogenic compound [11]. The enzymes involved in menaquinone biosynthesis have been identified in the CIRM-BIA1^T^ genome. DHNA is synthesized by the naphtoate synthase (or dihydroxynaphtoic acid synthethase) in *P. freudenreichii* encoded by *men*B (PFREUD_07540).

### Growth in Swiss-type cheese

Swiss cheese is the biotope where propionibacteria reach the highest population density, i.e., over 10^9^ colony-forming units (cfu)/g. However, *P. freudenreichii* shows poor growth in milk, even for the subspecies *shermanii* which is able to ferment lactose. *P. freudenreichii* is able to grow in Swiss cheese, mainly due to its ability to ferment lactic acid under anaerobic conditions, the abundance of peptides due to the activity of the lactic acid starter bacteria (*P. freudenreichii* does not possess any protease capable of hydrolyzing milk caseins [84]), the favorable temperature of ripening (around 22–24°C for several weeks), and the relatively low NaCl concentration in the curd. *P. freudenreichii* is also able to cope with the stressful conditions of the “cooking” step, when the curd is heated to ∼50–54°C for 30 min, although this ability is strain dependent. Thermotolerant strains differ from thermosensitive strains by constitutive over-expression of stress-related molecular chaperones and ATP-dependent proteases [71]. Furthermore, the existence of the dihydroxyacetone kinase locus (*dha*KL, PFREUD_07980 and PFREUD_07990) induced by stress and starvation in *P. freudenreichii* (Table S1) is also consistent with the acquisition of thermotolerance.

*P. freudenreichii* ferments lactate produced by lactic starter bacteria to propionate, acetate and CO_2_ (see “Reconstruction of specific metabolic pathways”). L-lactate enters the cells due to the expression of a L-lactate permease (PFREUD_18660). No gene encoding a D-lactate permease was found in the genome. The two isomers of lactate are converted into pyruvate by specific lactate dehydrogenases, two NAD-dependent L-lactate dehydrogenases encoded by two paralogs (PFREUD_11570 and PFREUD_12840), and one FAD-dependent D-lactate dehydrogenase (PFREUD_16710).

*P. freudenreichii* also plays a key role in the formation of other cheese flavor compounds. It releases free fatty acids through milk fat lipolysis and short branched-chain fatty acids through amino acid catabolism [5]. Lipolysis is thought to result mainly from the activity of a secreted lipolytic esterase active on milk fat (PFREUD_04340) [35]. Another putative esterase (PFREUD_04240) which is predicted to be surface-exposed may also be involved in lipolysis. Ten intracellular esterases, five with activity confirmed by expression in *E. coli*, and five putative esterases were also found in the *P. freudenreichii* genome [36]. Some of these esterases could be involved in the synthesis of the volatile esters associated with the fruity flavor of cheese. The only esterase gene previously identified in *P. freudenreichii* strain JS, *estA* [85], was absent from the genome of the CIRM-BIA1^T^ strain. The sequences flanking *estA* were close to the lactose locus (Fig. 2), with a transposase (PFREUD_24470) and an integrase (PFREUD_24460) inserted in place of the *estA* gene.

*P. freudenreichii* produces two short branched-chain fatty acids, 2-methylbutanoic acid and 3-methylbutanoic acid, imparting the “cheesy/sweaty” notes in many cheeses. These fatty acids are produced by the catabolism of isoleucine and leucine, respectively. Their synthesis is closely related to that of membrane fatty acids, which consist primarily of methyl-branched chain fatty acids in *P. freudenreichii* [86]. Two permease proteins (high-affinity branched-chain amino acid transport system permease proteins BraE and BraD, encoded by PFREUD_10860 and PFREUD_10870, respectively) enable leucine and isoleucine to be transported into the cell. The first steps in the synthesis of BCFA and membrane fatty acids are common. The pathway involves the activity of a branched-chain aminotransferase, encoded by PFREUD_13350, and a branched-chain ketoacid dehydrogenase complex, composed of three subunits, E1α1, E1β and E2 (encoded by PFREUD_02190, PFREUD_02200, and PFREUD_02210, respectively).

In lactic acid bacteria, adaptation to the rich dairy niche is associated with gene decay, leading to metabolic simplification and auxotrophy. In contrast, *P. freudenreichii* possesses the complete enzymatic arsenal for the *de novo* biosynthesis of aminoacids and vitamins (with the exception of two). This result shows that *P. freudenreichii* cannot be considered a milk-adapted species.

### Conclusions

Annotation of the *P. freudenreichii* genome reveals the hardiness of the bacterium, its ability to cope with different stresses (oxidative, bile salt, temperature), to withstand phage attack, to accumulate glycogen and polyphosphate under favorable conditions, to mobilize these compounds during starvation conditions and, lastly, to synthesize most vitamins and amino acids. Unlike lactic acid bacteria such as *L. bulgaricus*, Propionibacteria do not seem to be over-adapted to cheese conditions. The presence of beta galactosidase probably resulting from a horizontal transfer event seems to be the unique adaptive trait revealed by our study. The genetic basis of the *P. freudenreichii* capacity to produce aromatic compounds was in agreement with those described at the biochemical level: the Wood-Werkman cycle producing propionic acid, pathways for production of acetic acid and CO_2_, production of branched-chain fatty acids from leucine and isoleucine, and discovery of an extracellular lipolytic esterase. Regarding probiotic activity, the complete pathway for synthesis of a bifidogenic compound was reconstructed and a large number of surface proteins involved in adhesion and immunomodulatory activity, as in the *Lactobacillus* and *Bifidobacterium* genera, were identified. Functional validations should now be investigated using transcriptomic and inactivation-complementation approaches. These investigations should take into account the large panel of *P. freudenreichii* phenotypes for aromatic and probiotic properties among the thousands of strains stored and described in microbiological resource centers for this species. The versatility and the high adaptability of the species could also include non-alimentary perspectives.

## Materials and Methods

### Sequencing

The strain used for sequencing was *P. freudenreichii* subsp. *shermanii* CIRM-BIA1^T^ (equivalent names: ATCC9614, American Type Culture Collection, Rockville, MD; CIRM1, CIRM-BIA, INRA, Rennes; TL34, INRA).

The complete sequence of *P. freudenreichii* subsp. *shermanii* CIRM-BIA1^T^ (Genbank accession number FN806773) was determined after genomic DNA fragmentation by mechanical shearing or *Bam*HI partial digest for the construction of plasmid and large insert libraries, respectively. The 3 kb (A), 10 kb (B) and 25 kb (C) fragments were cloned onto pCNS (pSU18 derived, (A) and (B)) and pBBc (pBeloBac11 derived (C)). Vector DNAs were purified and end-sequenced (9216 (A), 29184 (B) and 5376 (C)) using dye-terminator chemistry on ABI3730 sequencers. A pre-assembly was made without repeat sequences as described by Vallenet et al. [87] using the Phred/Phrap/Consed software package (www.phrap.org). The finished genome sequence was achieved by primer walking using a GC sequence finishing kit (GEhealthcare) and transposition bombs. A complement of 4004 sequences (3648 transpositions and 356 primer walking) was needed for gap closure and quality assessment (11.8-fold coverage with less than 1.4/10,000 bp error in sequencing).

### Annotation

Automatic and manual annotations were conducted with the AGMIAL platform [88].

For the vitamin B12 synthesis pathway, the gene nomenclature from [10] was used, with the exception that CobA adoT (encoding a Cob(I)alamin adenosyltransferase) was replaced by CobA2. Genes involved in anti-oxidative properties were identified using OxygeneDB [89]. Subcellular localization was predicted using SurfG+ [69]. The replication origin was identified using Ori-finder [90]. Atlas of gene (Fig. 1) was realized using Circos software [91].

### *P. acnes* comparison

The Promer program from the Mummer package [92] was used to highlight synteny with *P. acnes*. The homology of *P. freudenreichii* predicted proteins with proteins putatively involved in the degradation of host molecules or in the mediation of inflammation was determined by bidirectional best hits with an e-value less than 10^−3^ and covering over 80% of the shortest sequence.

### Vitamin requirement

Vitamin requirements were evaluated by growing *P. freudenreichii* in a chemically defined medium, as previously described [86], pH 6.8, containing lactic acid, eight amino acids, minerals (K, P, Mg, Mn, Fe, Na, Co and Zn), and either i) control medium: nine vitamins (pyridoxal phosphate (B6), nicotinic acid (PP), pantothenate (B5), thiamine (B1), riboflavin (B2), p-aminobenzoic acid, folic acid (B9), biotine (H), and vitamin B12), or ii) test media (all but one of these nine vitamins), or iii) all but p-aminobenzoic acid (an intermediate in the synthesis of vit B9) and vit B9. The media were sterilized by filtration, inoculated with 1.0% of a culture grown in the same medium, and incubated at 30°C under semi-anaerobic (static cultures in air atmosphere) and anaerobic conditions for at least 12 days. Four successive transfers were performed under the same conditions. The growth was followed both by optical density at 650 nm and plate counting on Yeast Extract Lactate agar medium [3].

### Carbohydrate fermentation tests

Fermentation of various carbohydrates was tested using the Biolog plate test (Biolog Inc, Hayward, CA, USA) and api50CH (Biomérieux, Craponne, France), according to manufacturer's instructions.

## Supporting Information

Table S1 List of identified proteins from the *Propionibacterium freudenreichii* genome by 2D-LC MSMS. Three different experimental setups were used and are given in the last column. Setup “a” is nanoLC-ESI/MS/MS, “b” is MALDI/MS/MS and “c” is 2DLC-ESI/MS/MS (first dimension being cation exchange chromatography and second dimension being reverse phase liquid chromatography).
(0.09 MB PDF)

Table S2 Insertion sequences occurence.
(0.00 MB PDF)

Table S3 Bidirectional best hits results between *P. freudenreichii* proteins and *P. acnes* proteins. No *P. acnes* protein putatively involved in the degradation of host molecules or in the mediation of inflammation was found in *P. freudenreichii*.
(0.15 MB PDF)

Table S4 *Propionibacterium freudenreichii* carbon substrate degradation.
(0.03 MB PDF)

Table S5 Results of test of vitamin requirements by *P. freudenreichii* CIRM-BIA1.
(0.04 MB PDF)

Table S6 List of pyrophosphate and polyphosphate enzymes.
(0.03 MB PDF)
