# Supplementary material for: The Complete Genome of Propionibacterium freudenreichii CIRM-BIA1T, a Hardy Actinobacterium with Food and Probiotic Applications
Source: PLoS One. 2010 Jul 23;5(7):e11748. doi: 10.1371/journal.pone.0011748 (PMC2909200; doi:10.1371/journal.pone.0011748)
Supplement: Table S1 — List of identified proteins from the Propionibacterium freudenreichii genome by 2D-LC MSMS. Three different experimental setups were used and are given in the last column. Setup “a” is nanoLC-ESI/MS/MS, “b” is MALDI/MS/MS and “c” is 2DLC-ESI/MS/MS (first dimension being cation exchange chromatography and second dimension being reverse phase liquid chromatography). (0.09 MB PDF) [file pone.0011748.s001.pdf]

Gene	Description	pI	Mr (kDa)	Score	Sequence coverage (%)	Number of peptides	Experimental setup used for identification
PF#33	|Protein of unknown function|5.2 Protein of unknown function similar to proteins from other organisms	5,3	33,5	217,8	17,5	4	b
PF#40	iolB|iolB (Myo-inositol catabolism IolB protein)|2.1.1 Specific carbohydrate metabolic pathway	4,8	31,9	133,7	8,3	2	b
PF#42	|Protein of unknown function|5.2 Protein of unknown function similar to proteins from other organisms	4,7	23,2	1123,4	81,7	20	a, b, c
PF#43	rpiB1|Ribose-5-phosphate isomerase 1|2.1.2 Main glycolytic pathways	5,6	15,4	51,2	8,8	1	b
PF#46	acpP|Acyl carrier protein (ACP)|2.4 Metabolism of lipids	4,1	9,2	178,0	48,2	3	c
PF#47	PF47|Protein mrp homolog (ATP-binding protein)|4.1 Adaptation to atypical conditions	4,7	40,3	528,2	36,6	7	b, c
PF#51	rpmD|50S ribosomal protein L30|3.7.1 Ribosomal proteins	10,4	6,7	166,0	55,0	3	a, c
PF#52	rplO|50S ribosomal protein L15|3.7.1 Ribosomal proteins	10,3	15,5	216,3	29,9	4	c
PF#57	pmm|Phosphomannomutase (PMM)|2.1.1 Specific carbohydrate metabolic pathway	5,2	29,2	51,7	4,1	1	b
PF#61	pip2|Proline iminopeptidase|3.10 Protein degradation	5,3	39,0	226,4	15,3	5	b
PF#70	rbfA|Ribosome-binding factor A|3.6 RNA modification	4,8	16,3	65,2	13,7	2	b
PF#77	manA|Phosphomannose isomerase|2.1.1 Specific carbohydrate metabolic pathway	5,1	42,9	47,3	2,8	1	b
PF#79	sdhB|Succinate dehydrogenase, subunit B|1.4 Membrane bioenergetics (electron transport chain and ATP synthase)	5,4	27,3	199,0	21,8	3	a, b, c
PF#87	pptE|phosphoenolpyruvate-protein phosphoryltransferase PptE|	5,0	51,8	312,5	16,6	4	b
PF#95	rmlA (rfbA)|Glucose-1-phosphate thymidylyltransferase|2.1.1 Specific carbohydrate metabolic pathway	4,9	31,1	51,5	2,8	1	b, c
PF#101	|S-layer domain protein domain protein precursor|1.1 Cell wall	6,6	30,4	186,4	6,4	4	a, b
PF#107	sufD|FeS assembly protein SufD|1.2 Transport/binding proteins and lipoproteins	5,1	44,2	572,6	29,9	7	a, b, c
PF#109	sufC|ABC-type transport system involved in Fe-S cluster assembly, ATPase component, SufC|1.2 Transport/binding proteins and lipoproteins	5,2	26,9	373,2	34,9	6	b, c
PF#115	|Uridine phosphorylase|2.3 Metabolism of nucleotides and nucleic acids	4,8	28,3	76,7	7,8	2	b
PF#135	cspA|Cold shock-like protein CspA |4.1 Adaptation to atypical conditions	4,8	7,4	189,5	54,4	3	a, b, c
PF#137	none|Protein of unknown function|5.2 Protein of unknown function similar to proteins from other organisms	4,7	8,1	120,4	36,5	2	c
PF#146	||	5,0	32,9	1482,8	71,6	24	a
PF#147	murD|UDP-N-acetylmuramoylalanine--D-glutamate ligase (UDP-N- acetylmuramoyl-L-alanyl-D-glutamate synthetase) (D-glutamic acid- adding enzyme)|1.1 Cell wall	5,5	52,5	875,7	40,5	16	a
PF#148	gcvT|Glycine cleavage system T protein, (aminomethyltransferase)|2.2 Metabolism of amino acids and related molecules	5,5	41,1	360,9	23,6	7	a, b
PF#158	rplJ|50S ribosomal protein L10|3.7.1 Ribosomal proteins	4,5	21,9	553,5	62,3	9	b, c
PF#159	rplL|50S ribosomal protein L7/L12|3.7.1 Ribosomal proteins	4,5	13,7	303,6	63,4	5	a, b, c
PF#163	|Protein of unknown function|5.2 Protein of unknown function similar to proteins from other organisms	5,3	27,8	118,0	18,4	3	b
PF#167	ahpC|Alkyl hydroperoxide reductase subunit C (Peroxiredoxin) (Thioredoxin peroxidase) (Alkyl hydroperoxide reductase protein C22) (General stress protein 22)|4.2 Detoxification	4,6	20,7	334,5	33,2	5	b, c
PF#169	pf169|Carboxylic ester hydrolase|2.4 Metabolism of lipids	5,3	24,9	290,4	41,8	5	b
PF#174	metF|5,10-methylenetetrahydrofolate reductase|2.2 Metabolism of amino acids and related molecules	5,6	34,1	111,3	8,3	2	b
PF#179	cbiL|CbiL Precorrin-2 C20-methyltransferase|2.5 Metabolism of coenzymes and prosthetic groups	4,9	26,9	189,8	18,7	4	b

PF#180	cbiF|CbiF Precorrin-4 C11-methyltransferase|2.5 Metabolism of coenzymes and prosthetic groups	5,1	33,9	158,7	12,5	3	a
PF#183	PPA1366|Putative 2-hydroxyhepta-2,4-diene-1,7-dioate isomerase|	4,6	29,6	240,1	15,7	4	b
PF#186	none|Chlorite dismutase|4.2 Detoxification	5,5	25,7	332,1	34,5	6	b
PF#190	inlA|internalin protein|1.1 Cell wall	5,2	61,1	3759,4	73,8	60	a, b
PF#195	hsp20 2|Heat shock protein 20 2 (20 kDa chaperone 2)|3.9 Protein folding	4,9	16,8	452,7	49,3	8	b, c
PF#198	none|Protein of unknown function|5.2 Protein of unknown function similar to proteins from other organisms	5,2	13,8	81,9	24,2	2	b
PF#202	rpmE|50S ribosomal protein L31|3.7.1 Ribosomal proteins	9,2	8,2	49,5	14,9	1	c
PF#213	eno1|Enolase 1 |2.1.2 Main glycolytic pathways	4,5	45,9	1088,1	50,1	14	a, b, c
PF#219	slh|S-layer protein precursor|1.1 Cell wall	4,5	48,4	78,0	3,5	2	a
PF#241	none| Peptidase M23B family / metalloendopeptidase|1 Cell envelope and cellular processes	5,3	39,0	1064,0	58,4	14	a, b
PF#243	ppiA (cypB)|Probable peptidyl-prolyl cis-trans isomerase A|3.9 Protein folding	4,8	18,8	331,0	51,8	6	b, c
PF#244	none|Protein of unknown function|5.2 Protein of unknown function similar to proteins from other organisms	5,3	35,9	149,3	10,7	3	b, c
PF#252	aspA|Aspartate ammonia-lyase|2.2 Metabolism of amino acids and related molecules	5,1	53,1	306,7	12,9	5	b, c
PF#264	impA|Inositol-1-monophosphatase (IMPase) (Inositol-1- phosphatase) (I-1-Pase)|2.1 Metabolism of carbohydrates and related molecules	5,4	30,4	54,4	2,8	1	b
PF#277	murE|UDP-N-acetylmuramoylalanyl-D-glutamate--2,6-diaminopimelate ligase (UDP-N-acetylmuramyl-tripeptide synthetase) (Meso- diaminopimelate-adding enzyme) (UDP-MurNAc-tripeptide synthetase)|1.1 Cell wall	5,3	55,4	136,2	10,4	3	b
PF#279	pf279|Carboxylic ester hydrolase|2.4 Metabolism of lipids	5,4	46,4	1203,6	58,9	15	a, b
PF#286	dps|Starvation-inducible DNA-binding protein |4.1 Adaptation to atypical conditions	4,7	19,1	260,4	52,6	5	b, c
PF#289	||	5,0	7,4	294,1	50,0	4	a, c
PF#302	|UPF0237 protein|	4,6	9,8	68,1	14,6	1	c
PF#310	none|Aldose 1-epimerase|2.1 Metabolism of carbohydrates and related molecules	5,5	32,2	441,3	32,6	6	b
PF#323	none|HesB protein|4.1 Adaptation to atypical conditions	4,1	12,4	93,9	11,2	1	c
PF#326	pspA|Phage shock protein A|3.5.2 Transcription regulation	5,3	28,7	68,0	12,1	2	c
PF#342	pepC, (Blmh)|Aminopeptidase C (Bleomycin hydrolase)|2.2 Metabolism of amino acids and related molecules	5,1	50,0	700,4	41,3	14	a
PF#347	none|Short chain dehydrogenase|4.6 Miscellaneous	5,2	24,0	188,5	22,0	3	b, c
PF#348	rpiB2|Ribose-5-phosphate isomerase 2|2.1.2 Main glycolytic pathways	4,7	17,2	74,3	11,7	1	b
PF#350	glgB|1,4-alpha-glucan branching enzyme (Glycogen branching enzyme) (BE) (1,4-alpha-D-glucan:1,4-alpha-D-glucan 6-glucosyl-transferase)|2.1.1 Specific carbohydrate metabolic pathway	5,2	72,6	106,1	5,5	3	a, b
PF#359	metH|methionine synthase (5-methyltetrahydrofolate:L-homocysteine S-methyltransferase) |2.2 Metabolism of amino acids and related molecules	4,8	125,9	130,2	2,9	3	b
PF#377	glpB|Anaerobic glycerol-3-phosphate dehydrogenase subunit B|2.1.1 Specific carbohydrate metabolic pathway	5,3	45,9	961,9	53,7	16	a, b, c
PF#378	glpA|Anaerobic glycerol-3-phosphate dehydrogenase subunit A|2.1.1 Specific carbohydrate metabolic pathway	5,6	58,8	305,2	20,3	8	a, b, c
PF#379	pf379|Putative carboxylic ester hydrolase|2.4 Metabolism of lipids	6,0	33,7	257,7	26,7	6	b
PF#384	rplS|50S ribosomal protein L19|3.7.1 Ribosomal proteins	10,4	13,2	85,9	15,3	2	c
PF#390	fba1|Fructose-bisphosphate aldolase class II|2.1.2 Main glycolytic pathways	5,2	36,9	738,6	44,4	12	a, b, c
PF#397	argK|Kinase ArgK|1.2.5 Transport/binding of amino-acids	5,4	36,0	200,4	17,2	4	b
PF#399	asnB|Asparagine synthase (Glutamine-hydrolyzing)|2.2 Metabolism of amino acids and related molecules	5,1	72,6	52,8	1,7	1	c
PF#400	ilvD|Dihydroxy-acid dehydratase|2.2 Metabolism of amino acids and related molecules	5,1	65,0	278,9	13,5	6	a, b

PF#406	none|Protein of unknown function|5.2 Protein of unknown function similar to proteins from other organisms	5,1	39,4	992,6	55,5	19	a, b
PF#422	|Forkhead-associated protein|1.3 Sensors (signal transduction)	4,7	17,3	81,3	14,2	2	c
PF#429	gpt|Putative purine phosphoribosyltransferase|2.3 Metabolism of nucleotides and nucleic acids	4,6	18,2	53,2	9,5	1	c
PF#453	trx|Thioredoxin|3.8 Protein modification	4,0	15,5	75,8	8,5	1	c
PF#454	none|Forkhead-associated protein|1.3 Sensors (signal transduction)	5,6	26,0	48,5	4,6	1	b
PF#464	rmlB (rfbB)|DTDP-glucose 4,6-dehydratase|2.1.4 Substrate-specific entries to carbohydrate metabolic pathway	5,5	36,9	527,2	36,4	8	a, b
PF#465	|protein of unknown function|5.2 Protein of unknown function similar to proteins from other organisms	4,9	43,4	556,6	46,2	12	a, b, c
PF#476	greA|Transcription elongation factor GreA|3.5.2 Transcription regulation	4,6	18,1	56,3	12,0	1	c
PF#485	|Pyruvate flavodoxin/ferredoxin oxidoreductase|1.4 Membrane bioenergetics (electron transport chain and ATP synthase)	5,4	67,4	294,9	14,3	5	b
PF#492	iolA (msdA)|iolA (Myo-inositol catabolism IolA protein) (Methylmalonic acid semialdehyde dehydrogenase)|2.1.1 Specific carbohydrate metabolic pathway	5,0	52,8	150,5	9,6	4	a, c
PF#501	thrS|Threonyl-tRNA synthetase (Threonine--tRNA ligase) (ThrRS)|3.7.2 Aminoacyl-tRNA synthetases	5,1	75,8	110,8	2,8	1	b
PF#506	|GntR-family protein transcriptional regulator|3.5.2 Transcription regulation	8,0	27,4	65,3	14,0	2	c
PF#507	tal2|Transaldolase 2|2.1.2 Main glycolytic pathways	5,1	39,4	151,3	8,9	3	c
PF#508	iolG1|iolG1 (Myo-inositol catabolism IolG1 protein) (myo-inositol 2-dehydrogenase)|2.1 Metabolism of carbohydrates and related molecules	4,9	34,7	48,9	6,1	1	b
PF#518	|FAD-dependent pyridine nucleotide-disulphide oxidoreductase:4Fe-4S ferredoxin, iron-sulfur binding:Aromatic-ring hydroxylase|1.4 Membrane bioenergetics (electron transport chain and ATP synthase)	5,5	59,8	897,2	37,9	18	a, c
PF#527	|ABC-type transporter|1.2 Transport/binding proteins and lipoproteins	4,8	31,5	419,2	28,4	8	a
PF#528	none|ABC-type transporter|1.2 Transport/binding proteins and lipoproteins	4,7	31,4	445,4	28,1	8	a
PF#532	|Uncharacterized ATPase related to the helicase subunit of the holliday junction resolvase|3.3 DNA recombination, and repair	5,6	50,2	64,6	5,5	2	a
PF#537	pabC|Aminodeoxychorismate lyase|2.5 Metabolism of coenzymes and prosthetic groups	5,0	43,8	77,7	6,3	2	a
PF#540	|Short chain dehydrogenase|1.4 Membrane bioenergetics (electron transport chain and ATP synthase)	5,4	24,3	246,1	20,7	4	b
PF#561	degQ|secreted serine protease, trypsin-like serine proteases|1.2.1 Transport/binding of proteins/peptides	4,4	47,8	376,9	15,8	6	a
PF#566	parB|chromosome partitioning protein|3.4 DNA packaging and segregation	5,7	36,2	178,8	12,5	3	b
PF#569	rplI|50S ribosomal protein L9|3.7.1 Ribosomal proteins	9,4	16,1	209,3	29,3	4	c
PF#570	rpsR|30S ribosomal protein S18|3.7.1 Ribosomal proteins	11,0	9,0	121,2	29,1	2	c
PF#571	ssb|Single-strand binding protein|3.1 DNA replication	5,2	19,6	406,8	41,0	5	a, b, c
PF#603	pyrG|CTP synthase (UTP--ammonia ligase) (CTP synthetase)|2.3 Metabolism of nucleotides and nucleic acids	6,2	63,8	67,7	2,9	1	c
PF#623	cysS|Cysteine--tRNA ligase|3.7.2 Aminoacyl-tRNA synthetases	4,8	46,0	280,8	17,9	6	a
PF#635	lpdB|Dihydrolipoyl dehydrogenase|1.4 Membrane bioenergetics (electron transport chain and ATP synthase)	5,3	49,9	391,7	23,7	8	a
PF#649	yghU|glutathione S-transferase|4.2 Detoxification	6,9	20,0	214,1	23,8	3	b
PF#650	yghU|glutathione S-transferase|4.2 Detoxification	4,9	18,7	103,9	14,9	2	a, b
PF#658	||	9,4	11,1	78,9	15,1	1	c
PF#660	katA|Catalase|4.1 Adaptation to atypical conditions	5,1	53,6	1763,4	71,2	31	a, b
PF#661	|Peroxidase|4.6 Miscellaneous	4,6	32,9	195,3	16,4	5	b, c
PF#667	pf667|Carboxylic ester hydrolase|2.4 Metabolism of lipids	4,9	34,2	464,9	28,8	7	b

PF#668	ugpQ|Glycerophosphoryl diester phosphodiesterase|2.4 Metabolism of lipids	4,6	28,1	315,0	41,6	6	b, c
PF#684	glyQS|Glycyl-tRNA synthetase (Glycine--tRNA ligase) (GlyRS)|3.7.2 Aminoacyl-tRNA synthetases	5,5	52,9	248,0	11,0	4	b
PF#687	nuoI|NADH-quinone oxidoreductase subunit I (NADH dehydrogenase I subunit I) (NDH-1 subunit I)|1.4 Membrane bioenergetics (electron transport chain and ATP synthase)	5,7	21,5	71,6	12,6	2	b, c
PF#698	carB (pyrA)|Carbamoyl-phosphate synthase large chain (Carbamoyl- phosphate synthetase ammonia chain)|2.2 Metabolism of amino acids and related molecules	4,8	120,7	285,6	7,0	5	a, b
PF#699	carA|Carbamoyl-phosphate synthase small chain|1.2.6 Transport/binding of nucleosides, nucleotides, purines and pyrimidines	5,1	42,5	318,0	19,0	5	a, c
PF#701	efp|Elongation factor P|3.7.4 Translation elongation	4,7	20,6	158,2	12,8	3	b, c
PF#710	none|Hypothetical protein|5.2 Protein of unknown function similar to proteins from other organisms	4,8	56,9	641,0	26,6	10	a
PF#712	ppaX|pyrophosphatase PpaX|2.6 Metabolism of phosphate	4,5	23,2	53,8	5,5	1	a, b
PF#717	|Glutamine synthetase|2.8 Metabolism of nitrogen/nitrate and nitrite	4,8	54,1	418,1	21,3	6	b, c
PF#721	aroA|3-phosphoshikimate 1-carboxyvinyltransferase (phosphoenolpyruvate:3-phosphoshikimate5-O-(1-carboxyvinyl)-transferase) (EPSP synthase)|2.2 Metabolism of amino acids and related molecules	6,0	46,8	212,8	12,2	4	b
PF#731	mpg|3-methyladenine DNA glycosylase|3.3 DNA recombination, and repair	5,4	22,9	79,8	15,3	2	b
PF#733	||	5,2	14,9	186,2	25,4	4	a
PF#749	argS|Arginyl-tRNA synthetase (Arginine--tRNA ligase) (ArgRS)|3.7.2 Aminoacyl-tRNA synthetases	5,1	59,9	183,4	8,8	3	b
PF#758	rplD|50S ribosomal protein L4|3.7.1 Ribosomal proteins	9,8	24,8	247,7	25,3	5	c
PF#759	rplW|50S ribosomal protein L23|3.7.1 Ribosomal proteins	9,6	11,5	76,5	29,1	2	c
PF#760	rplB|50S ribosomal protein L2|3.7.1 Ribosomal proteins	11,1	30,4	259,4	26,3	5	c
PF#761	rpsS|30S ribosomal protein S19|3.7.1 Ribosomal proteins	10,8	10,6	129,9	43,0	3	c
PF#762	rplV|50S ribosomal protein L22|3.7.1 Ribosomal proteins	10,5	17,3	102,0	23,6	3	c
PF#763	rpsC|30S ribosomal protein S3|3.7.1 Ribosomal proteins	10,7	30,0	241,3	12,5	4	c
PF#764	rplP|50S ribosomal protein L16|3.7.1 Ribosomal proteins	10,5	15,7	191,7	48,9	4	c
PF#765	rpmC|50S ribosomal protein L29|3.7.1 Ribosomal proteins	6,6	9,3	211,7	36,6	3	c
PF#767	rplN|50S ribosomal protein L14|3.7.1 Ribosomal proteins	10,0	13,3	78,5	19,5	2	c
PF#768	rplX|50S ribosomal protein L24|3.7.1 Ribosomal proteins	10,4	13,2	128,3	28,5	2	c
PF#769	rplE|50S ribosomal protein L5|3.7.1 Ribosomal proteins	9,6	24,7	237,9	18,6	3	c
PF#770	rpsN1, rpsZ|30S ribosomal protein S14 type Z|3.7.1 Ribosomal proteins	11,3	6,9	76,7	21,3	1	c
PF#771	rpsH|30S ribosomal protein S8|3.7.1 Ribosomal proteins	9,3	14,8	201,3	35,6	3	c
PF#772	rplF|50S ribosomal protein L6|3.7.1 Ribosomal proteins	9,4	19,7	185,2	25,0	4	c
PF#773	rplR|Ribosomal protein L18|3.7.1 Ribosomal proteins	11,4	14,0	325,6	42,5	5	b, c
PF#778	||	6,2	33,7	1077,7	43,4	16	a
PF#779	none|Protein of unknown function |5.2 Protein of unknown function similar to proteins from other organisms	5,9	13,8	54,4	10,0	1	b
PF#803	none|Membran protein of unknown function|5.2 Protein of unknown function similar to proteins from other organisms	5,7	17,0	152,6	18,9	3	c
PF#808	|glutathione S-transferase |3.9 Protein folding	5,4	38,1	438,2	30,1	7	b
PF#823	none|two component system response regulator|3.5.2 Transcription regulation	5,0	26,9	78,5	16,5	2	b, c
PF#824	none|ABC-type transport systems, periplasmic component|1.2.5 Transport/binding of amino-acids	6,4	34,0	47,3	4,0	1	b
PF#842	|Thiamine pyrophosphate enzyme|	5,1	62,9	1108,2	41,4	22	a, b, c

PF#849	galE|UDP-glucose 4-epimerase|2.1.1 Specific carbohydrate metabolic pathway	5,4	35,1	674,3	50,0	11	a, b
PF#856	|protein of unknown function|5.2 Protein of unknown function similar to proteins from other organisms	6,0	32,8	63,9	5,7	2	b
PF#858	ppdk|Pyruvate phosphate dikinase|2.1 Metabolism of carbohydrates and related molecules	4,8	95,8	2845,1	79,7	52	a, b, c
PF#876	dac|carboxypeptidase (serine-type D-Ala-D-Ala carboxypeptidase) (D-alanyl-D-alanine-carboxypeptidase)|3.10 Protein degradation	5,4	46,0	317,9	14,5	5	a
PF#877	|Inorganic pyrophosphatase|2.6 Metabolism of phosphate	4,5	21,2	84,2	10,8	1	b, c
PF#886	ponA|Penicillin-binding protein (Transglycosylase/transpeptidase)|1.1 Cell wall	4,9	77,9	105,8	4,1	2	a
PF#893	CE1826, ptsI|PTS enzyme I|	5,1	57,7	552,8	30,0	12	a, b
PF#894	|Phosphocarrier, HPr family|1.2 Transport/binding proteins and lipoproteins	4,2	9,0	111,3	25,0	1	c
PF#896	rpsL|30S ribosomal protein S12|3.7.1 Ribosomal proteins	11,5	13,6	68,9	6,5	1	c
PF#897	rpoC|DNA-directed RNA polymerase beta chain (RNAP beta subunit) (Transcriptase beta chain) (RNA polymerase beta subunit)|3.5.3 Transcription elongation	7,8	143,5	198,1	4,2	4	c
PF#898	rpoB|DNA-directed RNA polymerase beta chain (RNAP beta subunit) (Transcriptase beta chain) (RNA polymerase subunit beta)|3.5.3 Transcription elongation	4,8	128,6	258,6	6,7	6	b, c
PF#906	|ATP binding protein of ABC transporter|1.2 Transport/binding proteins and lipoproteins	5,9	30,9	218,1	20,5	3	b
PF#907	|ABC transporter permease|1.2 Transport/binding proteins and lipoproteins	9,5	83,5	56,3	1,3	1	a
PF#908	dkgA|2,5-diketo-D-gluconate reductase A|2.5 Metabolism of coenzymes and prosthetic groups	5,0	30,4	534,8	60,0	12	a, b, c
PF#925	dnaK1|Chaperone protein dnaK 1 (Heat shock protein 70 1) (Heat shock 70 kDa protein 1) (HSP70 1)|3.9 Protein folding	4,8	65,4	2125,0	67,3	37	a, b, c
PF#928	hspR1|Heat shock protein transcriptional repressor HspR1 (Hspr1 protein)|3.5.2 Transcription regulation	11,1	15,8	66,3	20,1	2	c
PF#933	yghZ|Oxidoreductase|4.2 Detoxification	5,6	38,8	213,3	13,4	4	b
PF#934	gltA|Citrate synthase 1|2.1.3 TCA cycle	5,6	47,5	790,6	45,9	15	a, b
PF#935	none|Protein of unknown function|5.2 Protein of unknown function similar to proteins from other organisms	5,2	19,9	275,0	41,5	5	b
PF#936	fba2|Fructose-bisphosphate aldolase class I|2.1.2 Main glycolytic pathways	5,0	32,3	942,4	74,2	15	a, b, c
PF#944	aldB|Aldehyde dehydrogenase B (Lactaldehyde dehydrogenase)|2.1.1 Specific carbohydrate metabolic pathway	4,7	53,4	319,8	17,5	5	b
PF#961	|hypothetical protein|6 Protein of unknown function, without similarity to other proteins	5,2	8,5	65,9	17,1	1	c
PF#962	pf962|Carboxylic ester hydrolase|2.4 Metabolism of lipids	5,7	39,9	472,7	33,3	9	b
PF#963	|cell-wall peptidases, NlpC/P60 family secreted protein|1.1 Cell wall	4,8	58,8	2328,4	80,4	34	a, b
PF#971	none|Methylmalonyl-CoA carboxyltransferase 5S subunit. (trancarboxylase 5S) 505 bp|2.1.1 Specific carbohydrate metabolic pathway	5,4	55,6	1237,1	54,3	25	a, b, c
PF#972	none|Methylmalonyl-CoA carboxyltransferase 12S subunit (EC2.1.3.1) (Transcarboxylase 12S subunit). 610 bp|2.1.1 Specific carbohydrate metabolic pathway	5,4	56,4	1755,4	77,3	32	a, b, c
PF#973	none|hypothetical protein|	4,7	8,8	46,2	9,8	1	c
PF#974	bccp|Methylmalonyl-CoA carboxyltransferase , 1.3S subunit (Transcarboxylase, 1.3S subunit). 123bp|2.1.1 Specific carbohydrate metabolic pathway	5,4	12,4	217,7	39,8	3	b, c
PF#977	gpm2|phosphoglycerate mutase|2.1.2 Main glycolytic pathways	5,8	28,0	552,0	59,8	11	a, b, c
PF#982	|Nitroreductase|1.4 Membrane bioenergetics (electron transport chain and ATP synthase)	5,1	18,6	161,6	23,1	3	b
PF#988	cpsA|Carboxypeptidase|3.10 Protein degradation	5,1	44,3	258,0	17,7	5	b, c
PF#991	cys2|cysteine synthase 2|2.2 Metabolism of amino acids and related molecules	5,1	33,6	759,4	58,4	10	a, b, c
PF#995	|protein with unknown function|5.2 Protein of unknown function similar to proteins from other organisms	5,2	5,7	79,3	18,2	1	c
PF#1000	dapD|□2,3,4,5-tetrahydropyridine-2,6-dicarboxylate N-succinyltransferase|2.2 Metabolism of amino acids and related molecules	5,9	32,6	279,1	23,4	6	a, b

PF#1002	|aminotransferase|2.2 Metabolism of amino acids and related molecules	5,9	39,0	127,9	16,5	3	a
PF#1003	fdxA|Ferredoxin|1.4 Membrane bioenergetics (electron transport chain and ATP synthase)	4,0	11,7	72,8	12,3	1	c
PF#1004	gya|Glyoxylate reductase|	5,3	34,1	420,4	28,9	6	b, c
PF#1032	rpmI|50S ribosomal protein L35|3.7.1 Ribosomal proteins	11,8	7,1	49,3	20,3	1	a
PF#1033	rplT|50S ribosomal protein L20|3.7.1 Ribosomal proteins	10,8	13,8	56,8	14,6	1	c
PF#1036	pheS|Phenylalanyl-tRNA synthetase alpha chain (Phenylalanine--tRNA ligase alpha chain) (PheRS)|3.7.2 Aminoacyl-tRNA synthetases	5,7	39,8	145,6	11,5	2	b
PF#1042	gpm1|phosphoglycerate mutase/fructose-2,6-bisphosphatase|2.1.2 Main glycolytic pathways	4,8	26,1	90,9	10,5	2	b
PF#1044	trxB|Thioredoxin reductase|3.8 Protein modification	4,6	34,5	81,2	4,9	1	b
PF#1058	|secreted transglycosydase|1.1 Cell wall	5,7	20,0	1641,3	63,9	24	a, c
PF#1064	lipA|Lipoic acid synthetase|4.6 Miscellaneous	5,4	35,3	94,0	8,9	2	b
PF#1066	rplA|50S ribosomal protein L1|3.7.1 Ribosomal proteins	9,0	25,4	322,7	27,5	4	c
PF#1067	rplK|50S ribosomal protein L11|3.7.1 Ribosomal proteins	9,5	14,9	252,4	26,8	3	c
PF#1068	nusG|Transcription antitermination protein NusG|3.5.4 Transcription termination	4,3	32,4	169,6	11,9	3	c
PF#1077	gnd|6-phosphogluconic dehydrogenase 1|2.1.2 Main glycolytic pathways	5,3	52,6	818,0	41,4	13	a, b
PF#1084	cobA|CobA Uroporphyrinogen III methyltransferase|2.5 Metabolism of coenzymes and prosthetic groups	5,6	27,1	390,6	38,5	6	b, c
PF#1092	dnaK2|Chaperone protein dnaK 2 (Heat shock protein 70 2) (Heat shock 70 kDa protein 2) (HSP70 2)|3.9 Protein folding	4,7	67,2	1614,5	51,6	27	a, b, c
PF#1093	grpE2|Protein GrpE 2 (HSP-70 cofactor 2) (Co-chaperone protein GrpE2)|3.9 Protein folding	4,7	23,0	136,7	19,9	3	b, c
PF#1097	ask|Aspartokinase (Aspartate kinase)|2.2 Metabolism of amino acids and related molecules	5,1	44,9	74,7	3,8	1	c
PF#1107	folE|GTP cyclohydrolase I (GTP-CH-I)|2.5 Metabolism of coenzymes and prosthetic groups	6,1	24,9	67,3	10,2	2	b, c
PF#1111	folK|Putative hydroxymethyldihydropteridine pyrophosphokinase|2.5 Metabolism of coenzymes and prosthetic groups	5,1	19,8	92,0	9,4	2	b
PF#1114	clpC|chaperone clpC (Clp-family ATP-binding protease) (ATP-dependent Clp protease ATP-binding subunit)|3.9 Protein folding	5,2	94,3	202,3	6,5	4	a, c
PF#1122	pyrE|Orotate phosphoribosyltransferase (OPRT) (OPRTase)|2.3 Metabolism of nucleotides and nucleic acids	4,9	19,3	91,0	15,1	2	b
PF#1135	|GTP phosphohydrolase (mRNA-translation-assisting)|3.7.4 Translation elongation	4,9	75,8	378,7	18,0	9	a, b
PF#1136	rplM|50S ribosomal protein L13|3.7.1 Ribosomal proteins	9,7	16,1	303,3	47,6	4	c
PF#1139	glmM|Phosphoglucosamine mutase|2.2 Metabolism of amino acids and related molecules	5,0	47,2	58,9	4,4	1	c
PF#1140	hemB|Delta-aminolevulinic acid dehydratase (Porphobilinogen synthase)|2.5 Metabolism of coenzymes and prosthetic groups	4,9	35,8	241,6	23,8	5	b
PF#1144	gab|Aldehyde dehydrogenase (Succinate-semialdehyde dehydrogenase) (NAD-dependent aldehyde dehydrogenase)|2.1 Metabolism of carbohydrates and related molecules	4,9	52,2	258,8	13,9	6	a, b
PF#1146	polA|Putative DNA polymerase I|3.1 DNA replication	5,0	98,6	69,3	1,5	1	b
PF#1147	|two-component system response regulator|3.5.2 Transcription regulation	4,6	22,6	186,7	28,0	4	b, c
PF#1148	pyk1|Pyruvate kinase 1|2.1.2 Main glycolytic pathways	5,2	54,0	886,3	47,7	16	a, b, c
PF#1151	dskA|Transcriptional regulators, TraR/DksA family|3.5.2 Transcription regulation	4,7	15,7	111,5	15,5	2	c
PF#1152	none|Protein of unknown function|5.2 Protein of unknown function similar to proteins from other organisms	4,8	36,5	474,1	29,8	7	b, c
PF#1166	|2-dehydropantoate 2-reductase|	5,8	32,6	203,2	16,0	3	b
PF#1167	none|Transcriptional regulator, RpiR family|3.5.2 Transcription regulation	5,0	29,9	139,8	19,0	3	b

PF#1180	gyrA|DNA gyrase subunit A|3.1 DNA replication	5,0	100,6	63,4	1,4	1	c
PF#1193	|Restriction endonuclease PvuRts1 I|3.2 DNA restriction and modification (and repair)	5,6	35,3	356,3	23,1	7	a
PF#1197	none|Protein of unknown function|5.2 Protein of unknown function similar to proteins from other organisms	4,5	27,1	67,6	8,7	2	c
PF#1204	namA|NADH-dependent flavin oxidoreductase|1.4 Membrane bioenergetics (electron transport chain and ATP synthase)	4,8	39,1	69,0	5,9	1	c
PF#1214	ispG|4-hydroxy-3-methylbut-2-en-1-yl diphosphate synthase (1- hydroxy-2-methyl-2-(E)-butenyl 4-diphosphate synthase)|2.5 Metabolism of coenzymes and prosthetic groups	5,8	41,1	92,3	7,2	2	b, c
PF#1215	dxr|1-deoxy-D-xylulose 5-phosphate reductoisomerase (DXP reductoisomerase) (1-deoxyxylulose-5-phosphate reductoisomerase) (2-C- methyl-D-erythritol 4-phosphate synthase)|2.5 Metabolism of coenzymes and prosthetic groups	5,4	41,4	373,4	26,6	7	a
PF#1218	frr|Ribosome recycling factor (Ribosome-releasing factor) (RRF)|3.7.5 Translation termination	5,7	20,5	107,9	14,1	2	b
PF#1219	pyrH|Uridylate kinase PyrH|2.3 Metabolism of nucleotides and nucleic acids	4,8	27,0	81,6	16,5	2	b
PF#1220	rpsB|30S ribosomal protein S2|3.7.1 Ribosomal proteins	5,1	35,6	255,8	18,5	5	c
PF#1226	PPA1530|ABC transporter ATP-binding protein|1.2 Transport/binding proteins and lipoproteins	5,4	28,9	264,5	20,5	5	a, b
PF#1228	inhA|Enoyl-[acyl-carrier-protein] reductase [NADH]|2.4 Metabolism of lipids	4,7	26,8	334,1	30,4	4	b
PF#1235	|ABC transporter ATP-binding protein|1.2 Transport/binding proteins and lipoproteins	5,6	39,6	443,6	35,9	9	a, b, c
PF#1236	metB|Cystathionine gamma-synthase (O-succinylhomoserine (thiol)-lyase)|2.2 Metabolism of amino acids and related molecules	5,2	41,6	346,3	23,8	5	b
PF#1239	rpmA|50S ribosomal protein L27|3.7.1 Ribosomal proteins	11,6	9,3	122,4	29,9	2	a, c
PF#1244	proA|Gamma-glutamyl phosphate reductase (GPR) (Glutamate-5- semialdehyde dehydrogenase) (Glutamyl-gamma-semialdehyde dehydrogenase) (GSA dehydrogenase)|2.2 Metabolism of amino acids and related molecules	5,2	43,6	374,7	22,8	6	b
PF#1246	nadD|Probable nicotinate-nucleotide adenylyltransferase|2.5 Metabolism of coenzymes and prosthetic groups	5,1	28,0	55,1	6,3	1	b
PF#1253	leuS|Leucyl-tRNA synthetase (Leucine--tRNA ligase) (LeuRS)|3.7.2 Aminoacyl-tRNA synthetases	5,5	93,7	203,6	5,7	4	a, b
PF#1256	rnc|Ribonuclease III|3.5.2 Transcription regulation	5,2	26,0	108,7	13,8	3	b
PF#1260	||	4,7	15,2	62,0	10,0	1	b, c
PF#1264	|Beta-lactamase-like:RNA-metabolising metallo-beta-lactamase|	5,9	61,1	198,9	9,3	4	b, c
PF#1269	rpsO|30S ribosomal protein S15|3.7.1 Ribosomal proteins	9,9	10,2	59,5	10,3	1	c
PF#1277	||	4,1	19,5	110,3	14,1	2	c
PF#1293	phk|Phosphoketolase pyrophosphate|2.1.2 Main glycolytic pathways	5,6	96,7	194,8	5,9	4	b
PF#1300	menB|Naphthoate synthase (Dihydroxynaphthoic acid synthetase)|2.5 Metabolism of coenzymes and prosthetic groups	5,3	36,7	668,0	53,9	11	a, b, c
PF#1301	pncB|Nicotinate phosphoribosyltransferase|2.5 Metabolism of coenzymes and prosthetic groups	4,9	48,3	220,5	9,8	3	b
PF#1328	|protein of unknown function|5.2 Protein of unknown function similar to proteins from other organisms	5,3	37,8	2304,4	66,2	36	a, b
PF#1330	rplQ|50S ribosomal protein L17|3.7.1 Ribosomal proteins	9,6	21,4	199,1	19,5	3	c
PF#1333	rpsD|30S ribosomal protein S4|3.7.1 Ribosomal proteins	10,1	23,3	278,9	30,3	6	c
PF#1335	rpsM|30S ribosomal protein S13|3.7.1 Ribosomal proteins	10,7	14,0	96,2	12,1	1	b, c
PF#1337	infA|Translation initiation factor IF-1|3.5.2 Transcription regulation	9,5	8,4	73,1	16,4	1	c
PF#1339	|protein of unknown function|5.2 Protein of unknown function similar to proteins from other organisms	4,8	10,6	105,9	26,6	2	b, c
PF#1342	adk|Adenylate kinase (EC 2.7.4.3) (ATP-AMP transphosphorylase)|1.2.6 Transport/binding of nucleosides, nucleotides, purines and pyrimidines	4,7	20,4	65,5	13,1	2	b
PF#1347	oppA|Periplasmic oligopeptide-binding lipoprotein OppA|1.2.1 Transport/binding of proteins/peptides	5,1	61,5	454,4	16,3	8	a

PF#1350	clpP1|ATP-dependent Clp protease proteolytic subunit 1 (Endopeptidase Clp 1)|3.10 Protein degradation	5,1	22,6	526,5	51,7	9	a, b
PF#1351	clpP2|ATP-dependent Clp protease proteolytic subunit 2 (Endopeptidase Clp 2)|3.10 Protein degradation	4,7	24,8	164,4	22,7	3	b, c
PF#1403	dnaN|DNA polymerase III, beta chain|3.1 DNA replication	4,8	41,5	404,6	21,8	5	b, c
PF#1439	nuoC|NADH dehydrogenase I chain C|1.4 Membrane bioenergetics (electron transport chain and ATP synthase)	5,4	28,7	762,3	64,7	13	a, b, c
PF#1440	nuoD|NADH dehydrogenase I chain D|1.4 Membrane bioenergetics (electron transport chain and ATP synthase)	4,8	50,3	1280,7	63,5	25	a, b, c
PF#1441	nuoE|NADH-quinone oxidoreductase chain E|1.4 Membrane bioenergetics (electron transport chain and ATP synthase)	4,9	25,9	83,2	6,6	1	c
PF#1442	nuoF|NADH-quinone oxidoreductase chain F (NADH dehydrogenase I, chain F) (NDH-1, chain F)|1.4 Membrane bioenergetics (electron transport chain and ATP synthase)	5,7	48,6	491,0	28,9	11	a, b
PF#1445	serS1|Seryl-tRNA synthetase|3.7.2 Aminoacyl-tRNA synthetases	5,0	47,3	155,7	12,3	3	b
PF#1446	|hypothetical protein|5.2 Protein of unknown function similar to proteins from other organisms	4,9	24,4	188,8	25,7	5	b, c
PF#1453	tpx|thiol peroxidase|3.8 Protein modification	4,9	17,4	304,1	39,4	5	b
PF#1454	npdA|Silent information regulator protein Sir2 /NAD-dependent deacetylase|4.6 Miscellaneous	5,5	26,8	111,3	10,1	2	b
PF#1456	argG|Argininosuccinate synthase|2.2 Metabolism of amino acids and related molecules	5,0	52,6	257,8	13,2	5	b
PF#1457	|NADPH:quinone reductase and related Zn-dependent oxidoreductases |1.4 Membrane bioenergetics (electron transport chain and ATP synthase)	5,6	33,7	94,9	5,0	2	b
PF#1472	purS|Phosphoribosylformylglycinamidine synthetase PurS|2.3 Metabolism of nucleotides and nucleic acids	4,9	8,9	64,5	21,0	1	c
PF#1484	tkt|Transketolase|2.1.2 Main glycolytic pathways	5,0	74,1	306,3	10,8	5	a, b, c
PF#1486	araM|L-arabinose utilization protein|2.1.1 Specific carbohydrate metabolic pathway	5,0	49,7	848,9	40,4	13	a
PF#1500	atpF|ATP synthase B chain (F0F1 ATP synthase subunit B)|1.4 Membrane bioenergetics (electron transport chain and ATP synthase)	5,3	20,2	127,7	17,4	2	c
PF#1502	atpA|ATP synthase subunit alpha (ATPase subunit alpha) (ATP synthase F1 sector subunit alpha)|1.4 Membrane bioenergetics (electron transport chain and ATP synthase)	5,1	61,3	496,5	19,5	9	a, b, c
PF#1504	atpD|ATP synthase subunit beta (ATPase subunit beta) (ATP synthase F1 sector subunit beta)|1.4 Membrane bioenergetics (electron transport chain and ATP synthase)	4,7	52,5	383,5	17,4	6	a, c
PF#1505	atpC|ATP synthase epsilon chain (ATP synthase F1 sector epsilon subunit)|1.4 Membrane bioenergetics (electron transport chain and ATP synthase)	4,6	16,7	59,4	13,5	2	c
PF#1509	pf1509|Putative carboxylic ester hydrolase|2.4 Metabolism of lipids	6,4	29,6	330,6	30,4	7	b
PF#1512	none|Protein of unknown function|5.2 Protein of unknown function similar to proteins from other organisms	5,0	64,0	87,8	2,0	1	c
PF#1514	|Methylmalonyl-CoA epimerase|2.1.1 Specific carbohydrate metabolic pathway	5,4	16,7	475,8	85,1	9	a, b, c
PF#1515	trx|Thioredoxin|3.8 Protein modification	4,7	34,0	449,3	32,7	6	a, b, c
PF#1516	pgm1|Phosphoglucomutase|2.1.2 Main glycolytic pathways	5,0	58,6	280,2	13,1	4	a, b, c
PF#1521	rplY|Ribosomal protein L25|3.7.1 Ribosomal proteins	4,4	23,5	186,1	23,7	4	c
PF#1523	glmU|UDP-N-acetylglucosamine pyrophosphorylase|2.3 Metabolism of nucleotides and nucleic acids	5,1	54,5	134,2	8,2	4	a
PF#1536	galK|Galactokinase|2.1.1 Specific carbohydrate metabolic pathway	5,6	41,9	751,6	49,7	13	a, b
PF#1541	gdh|glutamate dehydrogenase (NAD(P)-glutamate dehydrogenase)|2.2 Metabolism of amino acids and related molecules	5,5	48,9	353,7	18,1	6	b
PF#1547	none|Protein of unknown function|5.2 Protein of unknown function similar to proteins from other organisms	5,7	68,7	77,9	3,1	2	b, c
PF#1556	||	5,2	37,3	46,1	2,4	1	c
PF#1571	tuf|Elongation factor Tu|3.7.4 Translation elongation	5,0	43,7	1691,1	78,8	27	a, b, c
PF#1587	pta|Phosphate acetyltransferase |2.1 Metabolism of carbohydrates and related molecules	4,7	52,8	224,2	10,4	4	b, c
PF#1588	ackA|Acetate kinase|2.2 Metabolism of amino acids and related molecules	5,4	42,4	1134,4	60,9	20	a, b, c

PF#1601	glf , rfb|UDP-galactopyranose mutase|1.1 Cell wall	5,3	45,3	756,2	41,5	13	a
PF#1602	none|ABC transporter|1.2.1 Transport/binding of proteins/peptides	5,0	64,8	173,0	8,5	4	a
PF#1612	none|Protein of unknown function|5.2 Protein of unknown function similar to proteins from other organisms	6,6	10,7	90,6	20,2	1	c
PF#1630	ldh|lactate dehydrogenase|2.1.2 Main glycolytic pathways	4,9	34,1	169,5	16,5	3	b
PF#1637	pf1637|Putative carboxylic ester hydrolase|2.4 Metabolism of lipids	5,0	32,1	326,5	39,9	7	b
PF#1640	trxA2|Thioredoxin|3.8 Protein modification	9,1	15,2	73,1	11,3	1	c
PF#1652	ugpA|UTP--glucose-1-phosphate uridylyltransferase|2.1 Metabolism of carbohydrates and related molecules	4,9	49,9	281,6	12,3	5	b
PF#1653	||	4,7	11,9	85,2	15,5	1	c
PF#1655	pf1655|Carboxylic ester hydrolase|2.4 Metabolism of lipids	4,6	29,4	321,7	25,7	6	b
PF#1658	trx|Thiol-disulfide isomerase|3.8 Protein modification	4,2	12,2	65,9	9,9	1	c
PF#1659	dtxR|Iron-dependent repressor|3.5.2 Transcription regulation	4,8	24,7	46,0	6,2	1	c
PF#1671	|Sua5_yciO_yrdC|3.7.3 Translation initiation	5,1	22,6	141,5	10,7	2	b
PF#1673	pfp (pfk)|pyrophosphate phosphofructokinase|2.1 Metabolism of carbohydrates and related molecules	4,9	43,2	466,7	20,8	6	a, b, c
PF#1718	gabT|4-aminobutyrate aminotransferase|2.2 Metabolism of amino acids and related molecules	5,3	46,7	639,3	30,3	10	a, b, c
PF#1721	prfB|Peptide chain release factor 2 (RF-2)|3.7.6 Nonribosomal protein synthesis	4,6	41,0	74,0	4,6	1	c
PF#1733	|ribosomal S30AE, sigma 54 modulation protein|3.7.3 Translation initiation	7,9	22,0	141,8	12,1	2	c
PF#1734	|Preprotein translocase SecA subunit|1.2 Transport/binding proteins and lipoproteins	4,9	106,5	79,7	2,3	2	a, b, c
PF#1737	||	4,6	7,2	74,7	26,9	1	c
PF#1738	|protein of unknown function|5.2 Protein of unknown function similar to proteins from other organisms	5,4	17,2	130,6	22,8	2	b
PF#1739	PPA1328|Predicted metal-dependent phosphoesterase|	6,0	31,1	133,7	14,0	3	b
PF#1740	trpS|Tryptophanyl-tRNA synthetase|3.7.2 Aminoacyl-tRNA synthetases	5,4	40,8	421,9	23,1	8	a, b
PF#1746	lpd|Dihydrolipoyl dehydrogenase (E3 component of alpha keto acid dehydrogenase complexes) (Dihydrolipoamide dehydrogenase)|2.1 Metabolism of carbohydrates and related molecules	5,1	49,6	1728,0	72,5	30	a, b, c
PF#1747	braC|Leucine-, isoleucine-, valine-, threonine-, and alanine-binding protein [Precursor] (LIVAT-BP)|1.2.5 Transport/binding of amino-acids	6,1	40,8	80,9	8,3	2	a
PF#1753	|transcriptional regulator|3.5.2 Transcription regulation	6,1	23,4	96,5	15,3	2	b
PF#1760	gatB|Aspartyl/glutamyl-tRNA(Asn/Gln) amidotransferase subunit B (Asp/Glu-ADT subunit B)|3.7 Protein synthesis	5,0	54,8	234,3	10,1	3	b, c
PF#1761	gatA|Glutamyl-tRNA(Gln) amidotransferase subunit A (Glu-ADT subunit A)|3.7 Protein synthesis	4,8	53,0	161,7	7,1	2	b, c
PF#1763	|Amino acid-binding ACT|3.8 Protein modification	4,7	22,8	68,2	11,5	2	b
PF#1774	tyrS|Tyrosyl-tRNA synthetase (Tyrosine--tRNA ligase) (TyrRS)|3.7.2 Aminoacyl-tRNA synthetases	5,7	46,0	649,4	41,9	11	a, b, c
PF#1778	none|S-layer protein |1.1 Cell wall	4,8	50,5	112,9	5,4	3	a
PF#1819	hisD|Histidinol dehydrogenase (HDH)|2.2 Metabolism of amino acids and related molecules	5,0	46,2	235,5	11,4	3	b
PF#1825	|protein of unknown function|5.2 Protein of unknown function similar to proteins from other organisms	4,6	29,0	101,0	6,8	1	c
PF#1838	|outer membrane protein of unknown function |1.1 Cell wall	4,8	47,8	232,7	8,5	4	a
PF#1854	||	4,2	18,4	52,4	8,9	1	c
PF#1861	pf1861|Putative carboxylic ester hydrolase|2.4 Metabolism of lipids	5,9	116,7	250,6	7,2	6	b
PF#1876	|Secreted protease with a PDZ domain|3.10 Protein degradation	4,6	36,3	73,8	3,5	1	a

PF#1882	pip1|Proline iminopeptidase|3.10 Protein degradation	5,3	45,5	613,1	41,3	12	a, b
PF#1885	|ABC transporter, substrate binding protein|1.2.5 Transport/binding of amino-acids	5,0	54,9	890,4	33,5	15	a, b
PF#1890	serA|D-3-phosphoglycerate dehydrogenase / erythronate 4-phosphate dehydrogenase|2.5 Metabolism of coenzymes and prosthetic groups	6,1	42,6	236,4	17,9	5	b
PF#1909	|zinc-binding dehydrogenase|4.6 Miscellaneous	5,4	37,1	839,5	47,0	12	a, b
PF#1915	bccA|Acetyl-/propionyl-coenzyme A carboxylase alpha chain |2.4 Metabolism of lipids	5,0	63,4	73,3	3,0	2	a
PF#1933	|transcriptional regulator TetR family|3.5.2 Transcription regulation	5,9	21,1	56,6	7,2	1	c
PF#1944	pdxS|Pyridoxal biosynthesis lyase pdxS|2.5 Metabolism of coenzymes and prosthetic groups	5,1	31,3	354,3	25,0	5	b, c
PF#1946	zwf|Glucose-6-phosphate 1-dehydrogenase|2.1.2 Main glycolytic pathways	6,0	60,3	235,4	8,9	4	b
PF#1947	opcA|glucose 6-phosphate dehydrogenase effector OpcA|2.1.2 Main glycolytic pathways	5,4	32,2	156,8	17,7	4	b
PF#1951	tpi1|triosephosphate isomerase 1 |2.1.2 Main glycolytic pathways	5,1	27,6	421,7	39,7	7	b, c
PF#1952	pgk|Phosphoglycerate kinase|2.1.2 Main glycolytic pathways	5,2	42,4	471,9	24,8	8	a, b, c
PF#1964	|ABC transporter, ATP-binding protein|1.2 Transport/binding proteins and lipoproteins	5,4	31,6	86,7	7,2	2	b
PF#1971	uvrB|UvrABC system protein B (Protein uvrB) (Excinuclease ABC subunit B)|3.2 DNA restriction and modification (and repair)	5,0	78,3	144,2	5,9	3	b
PF#2003	none|Protein of unknown function|5.2 Protein of unknown function similar to proteins from other organisms	5,6	36,7	464,5	28,2	8	a, b
PF#2005	bkdA2|2-oxoisovalerate dehydrogenase subunit beta (EC 1.2.4.4) (Branched- chain alpha-keto acid dehydrogenase E1 component beta chain) (BCKDH E1-beta) Pyruvate dehydrogenase E1 component subunit beta |2.2 Metabolism of amino acids and related molecu	5,3	35,6	123,2	12,7	3	b
PF#2006	bkdA1|2-oxoisovalerate dehydrogenase subunit alpha (Branched- chain alpha-keto acid dehydrogenase E1 component alpha chain) (BCKDH E1-alpha)|2.2 Metabolism of amino acids and related molecules	4,6	40,5	363,9	19,2	6	b, c
PF#2042	pf2042|Putative carboxylic ester hydrolase|2.4 Metabolism of lipids	4,6	35,2	216,0	23,4	4	b
PF#2048	cypA (yjiB)|Cytochrome P450 (Heme-thiolate monooxygenase)|1.4 Membrane bioenergetics (electron transport chain and ATP synthase)	4,8	44,2	1175,6	58,7	20	a, b, c
PF#2054	mrc/ponA|penicillin-binding protein (□peptidoglycan glycosyltransferase)|1.1 Cell wall	7,7	75,3	67,0	2,3	1	a
PF#2061	slh|Polysaccharide deacetylase precursor (S-layer domain protein)|1.1 Cell wall	6,0	12,4	448,6	96,4	8	a, b
PF#2062	inlA|Internalin protein|1.1 Cell wall	5,1	72,5	2382,5	75,2	31	a, b
PF#2074	|hypothetical secreted protein|6 Protein of unknown function, without similarity to other proteins	6,7	30,0	211,9	11,8	3	a
PF#2085	|Phosphoribosyltransferase/nitroreductase (fusion gene) (Nicotinate-nucleotide-dimethylbenzimidazole phosphoribosyltransferase)|2.5 Metabolism of coenzymes and prosthetic groups	5,1	66,4	846,6	35,0	16	a
PF#2086	pepC|Aminopeptidase C, Bleomycin hydrolase, |3.10 Protein degradation	4,7	53,4	80,9	4,4	1	c
PF#2091	opuCA|Glycine betaine/carnitine/choline transport ATP-binding protein opuCA|1.2.5 Transport/binding of amino-acids	5,5	48,0	593,0	30,0	10	a, b, c
PF#2094	groS1(groES1) (hsp10)|10 kDa chaperonin 1 (Protein Cpn10 1) (groES protein 1) (Heat shock 10 1)|3.9 Protein folding	4,9	10,6	374,3	63,3	5	a, b, c
PF#2096	guaB|Inosine-5 -monophosphate dehydrogenase (IMP dehydrogenase) (IMPDH) (IMPD) / GMP reductase|2.3 Metabolism of nucleotides and nucleic acids	5,5	53,9	289,6	13,4	4	b
PF#2097	guaB1|Inosine-5 -monophosphate dehydrogenaseInosine-5 -monophosphate dehydrogenase (IMP dehydrogenase) (IMPDH) (IMPD) / GMP reductase|2.3 Metabolism of nucleotides and nucleic acids	5,6	39,5	837,4	52,9	12	a, b
PF#2105	cys1|Cysteine synthase 1|2.2 Metabolism of amino acids and related molecules	5,1	31,7	290,8	23,7	4	b
PF#2129	|Phospholipase D/Transphosphatidylase|2.4 Metabolism of lipids	9,9	47,4	67,6	1,7	1	a
PF#2133	purH|Phosphoribosylaminoimidazolecarboxamide formyltransferase (AICAR transformylase)|2.3 Metabolism of nucleotides and nucleic acids	5,4	54,5	1095,2	58,2	19	a
PF#2148	|protein of unknown function|5.2 Protein of unknown function similar to proteins from other organisms	4,9	23,2	102,9	16,0	3	b

PF#2152	asd|Aspartate-semialdehyde dehydrogenase (Semialdehyde dehydrogenase)|2.2 Metabolism of amino acids and related molecules	5,2	36,6	417,3	26,7	6	b, c
PF#2153	|Delta 1-pyrroline-5-carboxylate reductase|2.2 Metabolism of amino acids and related molecules	5,1	28,9	97,4	10,5	2	b
PF#2170	|GCN5-related N-acetyltransferase|4.6 Miscellaneous	5,5	34,3	147,9	15,7	3	b
PF#2174	|inositol-1-phosphate synthase|2.1.1 Specific carbohydrate metabolic pathway	4,8	39,2	933,3	63,2	16	a, b, c
PF#2198	galK|Galactokinase|2.1.1 Specific carbohydrate metabolic pathway	5,0	41,0	111,4	7,8	2	b
PF#2200	nadB|L-aspartate oxidase|2.2 Metabolism of amino acids and related molecules	5,2	54,7	261,5	13,6	6	a, b
PF#2204	cysN|Sulfate adenylyltransferase, large subunit/ ATP-sulfurylase, subunit 1 (ATP:sulfate adenylyltransferase)|2.7 Metabolism of sulfur	4,7	48,1	510,9	28,7	7	b
PF#2206	cysH|Putative phosphoadenosine phosphosulfate reductase|2.7 Metabolism of sulfur	4,9	28,1	186,8	11,2	2	b
PF#2211	cysA|Sulfate/thiosulfate import ATP-binding protein CysA (Sulfate-transporting ATPase)|2.7 Metabolism of sulfur	5,3	37,9	248,4	18,6	5	b
PF#2215	pntA|NAD(P) transhydrogenase subunit alpha (Pyridine nucleotide transhydrogenase subunit alpha) (Nicotinamide nucleotide transhydrogenase subunit alpha)|2.5 Metabolism of coenzymes and prosthetic groups	5,4	54,2	284,9	14,3	6	c
PF#2237	|Pyridine nucleotide-disulphide oxidoreductase|4.6 Miscellaneous	4,9	48,2	409,6	25,5	7	b, c
PF#2241	galE|UDP-glucose 4-epimerase|2.1.4 Substrate-specific entries to carbohydrate metabolic pathway	4,8	35,8	102,1	14,2	3	b
PF#2252	caiA|Crotonobetainyl-CoA dehydrogenase (Crotonobetainyl-CoA reductase)|2.4 Metabolism of lipids	4,9	42,7	119,1	10,1	3	b
PF#2276	sseB|Thiosulfate sulfurtransferase |1.2.3 Transport/binding of inorganic ions	5,1	29,0	250,7	19,1	3	b
PF#2282	caiC|Acyl-CoA synthase|2.4 Metabolism of lipids	5,1	61,0	296,1	10,6	5	b
PF#2291	|serine/threonine protein kinase|2.2 Metabolism of amino acids and related molecules	4,9	74,0	52,3	2,0	1	a
PF#2293	jag|Single-stranded nucleic acid binding R3H|	3,9	33,8	305,8	24,1	6	c
PF#2295	|membrane protein (s-layer)|1.1 Cell wall	4,9	59,3	56,6	1,6	1	a, b
PF#2307	ald|Alanine dehydrogenase|1.1 Cell wall	4,8	39,4	1088,7	56,3	19	a, b, c
PF#2315	gpsA|Glycerol-3-phosphate dehydrogenase [NAD(P)+] (NAD(P)H- dependent glycerol-3-phosphate dehydrogenase)|2.4 Metabolism of lipids	5,0	36,1	99,3	7,1	2	b
PF#2316	xylB|Xylulokinase protein, Carbohydrate kinase|2.1 Metabolism of carbohydrates and related molecules	5,2	55,4	266,8	12,9	5	a
PF#2342	lacZ|Beta-galactosidase (Lactase) LacZ|2.1.1 Specific carbohydrate metabolic pathway	4,9	114,5	401,6	10,0	9	a
PF#2346	fixA (ydiQ)|Electron transfer flavoprotein (FixA protein)|1.4 Membrane bioenergetics (electron transport chain and ATP synthase)	4,7	25,7	83,4	6,0	2	b
PF#2369	PF2369|Putative aldo/keto reductase (oxidoreductase)|4.6 Miscellaneous	5,5	31,2	802,1	67,6	15	a, b
PF#2370	actA|Coenzyme A transferase (Putative succinyl-CoA or butyryl-CoA:coenzyme A transferase)|2.4 Metabolism of lipids	5,5	55,7	1944,3	74,6	35	a, c
PF#2381	purB|Adenylosuccinate lyase|2.3 Metabolism of nucleotides and nucleic acids	5,5	51,9	114,8	9,2	2	b
PF#2397	pgi, PPA2131|Glucose-6-phosphate isomerase (EC 5.3.1.9) (GPI) (Phosphoglucose isomerase) (PGI) (Phosphohexose isomerase) (PHI)|2.1.2 Main glycolytic pathways	5,2	61,8	509,8	24,5	11	a, b
PF#2402	|NADH-flavin reductase|2.5 Metabolism of coenzymes and prosthetic groups	5,0	21,6	83,6	12,3	2	b
PF#2412	nuoG|NADH dehydrogenase I chain G|1.4 Membrane bioenergetics (electron transport chain and ATP synthase)	5,1	84,9	308,5	8,7	5	b, c
PF#2413	|ABC transporter ATP-binding protein|1.2 Transport/binding proteins and lipoproteins	5,2	25,0	78,6	9,3	2	b, c
PF#2416	pf2416|Protein of unknown function|5.2 Protein of unknown function similar to proteins from other organisms	6,4	28,5	92,8	9,6	2	b
PF#2419	tal1|Transaldolase 1|2.1.2 Main glycolytic pathways	4,8	38,0	528,4	28,1	6	b
PF#2423	ahpF|Alkyl hydroperoxide reductase subunit F|4.2 Detoxification	4,9	59,9	140,7	8,5	3	b
PF#2424	rpsG|30S ribosomal protein S7|3.7.1 Ribosomal proteins	10,3	17,4	378,4	64,1	8	c

PF#2426	rpsJ|30S ribosomal protein S10|3.7.1 Ribosomal proteins	9,6	11,7	159,1	32,0	3	c
PF#2442	iolD|iolD (Myo-inositol catabolism iolD protein) (acetolactate synthase protein) (pyruvate:pyruvate acetaldehydetransferase (decarboxylating))|2.1.1 Specific carbohydrate metabolic pathway	5,0	69,8	803,8	35,0	16	a, b
PF#2444	clpB 1|Chaperone clpB 1 (ATP-dependent Clp protease B1) (Clp chaperone)|3.9 Protein folding	5,1	93,5	2980,8	66,6	50	a, b, c
PF#2463	pstS|Phosphate-binding transport protein of ABC transporter system|1.2.3 Transport/binding of inorganic ions	5,1	36,5	114,3	7,7	2	a
PF#2478	mao|flavin-containing amine oxidase|2.2 Metabolism of amino acids and related molecules	5,3	48,3	92,1	7,6	2	b
PF#2494	ribAB|GTP cyclohydrolase II protein, Riboflavin biosynthesis protein|2.5 Metabolism of coenzymes and prosthetic groups	5,5	50,9	166,6	6,5	2	b
PF#2500	mmcV/cysD|Sulfate adenylyltransferase subunit 2 (Sulfate adenylate transferase) (SAT) (ATP-sulfurylase small subunit) (Mitomycin biosynthesis protein V)|2.7 Metabolism of sulfur	5,2	39,3	130,8	10,7	2	b
PF#2521	araB, xylB ?|Carbohydrate kinase|2.1.1 Specific carbohydrate metabolic pathway	5,0	56,5	90,5	5,0	2	a
PF#2531	malY|Bifunctional PLP-dependent enzyme with beta-cystathionase and maltose regulon repressor activities (cystathionine beta-lyase, CBL)|2.2 Metabolism of amino acids and related molecules	4,9	45,0	379,6	18,9	5	b
PF#2535	thiE|thiamine-phosphate diphosphorylase|2.5 Metabolism of coenzymes and prosthetic groups	5,8	23,2	99,3	16,3	2	b
PF#2552	none|nucleoside-diphosphate-sugar epimerases|1.1 Cell wall	5,3	27,7	82,4	8,2	1	c
PF#2566	degQ|Trypsin-like serine protease|1.2.1 Transport/binding of proteins/peptides	5,3	28,7	91,3	7,2	2	a
PF#2570	||	5,3	26,7	126,8	19,6	3	a
PF#2571	clpB 2|Chaperone clpB 2 (ATP-dependent Clp protease B2) (Clp chaperone)|3.9 Protein folding	5,3	94,3	2229,2	54,7	39	a, b, c
PF#2579	groL2 (groEL2) (hsp60)|60 kDa chaperonin 2 (Protein Cpn60 2) (groEL protein 2) (Heat shock protein 60 2)|3.9 Protein folding	4,7	56,5	857,5	33,3	13	a, b, c
PF#2582	|iron-sulfur protein|1.4 Membrane bioenergetics (electron transport chain and ATP synthase)	6,7	57,3	574,4	23,4	8	b, c
PF#2583	|Oxidoreductase|4.6 Miscellaneous	5,6	28,9	803,3	62,8	15	a, b, c
PF#2590	hemE|Uroporphyrinogen decarboxylase|2.5 Metabolism of coenzymes and prosthetic groups	5,4	66,1	152,0	7,9	3	b
PF#2596	dld|d-lactate dehydrogenase|2.1 Metabolism of carbohydrates and related molecules	5,7	63,7	519,4	23,8	9	b, c
PF#2605	maoC|MaoC acyl dehydratase|2.4 Metabolism of lipids	5,4	32,9	98,0	7,9	2	b
PF#2629	tig|Trigger factor (TF)|3.9 Protein folding	4,6	57,4	274,8	15,4	5	b, c
PF#2642	|DSBA-like thioredoxin|4.6 Miscellaneous	4,8	23,0	227,9	30,4	5	b
PF#2654	orn|Oligoribonuclease|3.6 RNA modification	4,8	24,8	99,8	12,6	2	b, c
PF#2657	mutB|Methylmalonyl-CoA mutase large subunit (Methylmalonyl-CoA mutase alpha subunit) (MCM-alpha) (MUTB-(R)-2-Methyl-3-oxopropanoyl-CoA CoA-carbonylmutase)|2.1.1 Specific carbohydrate metabolic pathway	5,2	80,2	1332,7	44,1	20	a, b, c
PF#2658	mutA|Methylmalonyl-CoA mutase small subunit (Methylmalonyl-CoA mutase beta subunit) (MCB-beta)|2.1.1 Specific carbohydrate metabolic pathway	5,0	69,6	1690,3	57,5	31	a, b, c
PF#2671	icd|Putative isocitrate/isopropylmalate dehydrogenase|2.1.3 TCA cycle	5,2	44,8	742,9	47,2	15	a, b, c
PF#2675	metG|Methionyl-tRNA synthetase (Methionine--tRNA ligase) (MetRS)|3.7.2 Aminoacyl-tRNA synthetases	4,7	62,6	181,4	8,4	4	b
PF#2684	guaA|GMP synthase [glutamine-hydrolyzing] (Glutamine amidotransferase) (GMP synthetase)|2.3 Metabolism of nucleotides and nucleic acids	4,9	55,6	257,6	12,6	4	b
PF#2691	groL1 (groEL1)|60 kDa chaperonin 1 (Protein Cpn60 1) (groEL protein 1) (Heat shock protein 60 1)|3.9 Protein folding	4,7	56,1	1010,4	48,7	15	a, b, c
PF#2698	rspI|30S ribosomal protein S9|3.7.1 Ribosomal proteins	9,9	19,5	258,6	43,2	5	c
PF#2700	rpoA|DNA-directed RNA polymerase alpha chain (RNAP alpha subunit) (Transcriptase alpha chain) (RNA polymerase subunit alpha)|3.5.3 Transcription elongation	4,4	37,0	391,2	38,6	8	b, c
PF#2701	sodA|Iron/Manganese superoxide dismutase (Superoxide dismutase [Mn/Fe]) (SODM)|4.2 Detoxification	5,3	22,8	1122,3	71,3	22	a, b, c
PF#2703	| ABC transporter, ATPase subunit|1.2 Transport/binding proteins and lipoproteins	5,6	75,4	69,7	2,9	2	b

PF#2708	citE|Citrate lyase beta chain (Citrase beta chain) (Citrate(Pro-3S)-lyase beta chain)|2.1.3 TCA cycle	5,2	31,1	85,8	7,3	2	c
PF#2722	none|Response regulator receiver protein|3.5.2 Transcription regulation	4,9	18,8	97,8	20,1	2	b, c
PF#2723	guaB|Inosine monophosphate dehydrogenase|2.3 Metabolism of nucleotides and nucleic acids	6,0	51,7	249,6	16,8	4	b
PF#2727	ftsZ|Cell division protein FtsZ|1.7 Cell division	4,5	42,8	304,2	19,6	4	b, c
PF#2730	murF|UDP-N-acetylmuramoyl-tripeptide--D-alanyl-D-alanine ligase (UDP- MurNAc-pentapeptide synthetase) (D-alanyl-D-alanine-adding enzyme)|1.1 Cell wall	5,8	52,0	335,0	12,9	5	b
PF#2732	ftsI|Cell division protein FtsI (penicillin-binding protein 2) (Peptidoglycan glycosyltransferase)|1.1 Cell wall	4,9	65,6	139,3	7,7	3	a
PF#2734	infB|Translation initiation factor IF-2|3.7 Protein synthesis	9,0	101,8	50,3	1,5	1	c
PF#2738	tsf|Elongation factor Ts (EF-Ts)|3.7.4 Translation elongation	4,8	28,9	317,8	22,2	4	b, c
PF#2739	fabG|3-oxoacyl-[acyl-carrier protein] reductase|2.4 Metabolism of lipids	5,6	25,6	81,2	6,6	1	c
PF#2740	|Dehydrogenase|4.6 Miscellaneous	5,0	25,3	141,9	15,3	3	b
PF#2741	sufS|Cysteine desulphurases, SufS|1.2 Transport/binding proteins and lipoproteins	5,4	45,2	683,1	39,2	13	a, b, c
PF#2743	sufB|FeS assembly protein SufB|	5,0	53,9	125,2	7,4	3	c
PF#2745	pdxT|Glutamine amidotransferase subunit pdxT (Glutamine amidotransferase glutaminase subunit pdxT)|2.5 Metabolism of coenzymes and prosthetic groups	5,8	21,2	171,8	15,5	2	b
PF#2748	gap|Glyceraldehyde-3-phosphate dehydrogenase / erythrose 4 phosphate dehydrogenase|2.5 Metabolism of coenzymes and prosthetic groups	5,3	36,1	1942,4	84,1	29	a, b, c
PF#2757	sdhB3|Succinate dehydrogenase iron-sulfur protein|1.4 Membrane bioenergetics (electron transport chain and ATP synthase)	5,6	27,1	981,9	78,0	16	a, b, c
PF#2758	sdhA3|Succinate dehydrogenase flavoprotein subunit|2.1.3 TCA cycle	6,0	74,8	540,3	19,6	12	a, b, c
PF#2761	none|Protein of unknown function|5.2 Protein of unknown function similar to proteins from other organisms	8,6	9,5	97,1	27,4	1	c
PF#2762	rpsP|30S ribosomal protein S16|3.7.1 Ribosomal proteins	8,7	17,7	100,3	21,5	2	c
PF#2764	ffh|GTP binding signal recognition particle protein|3.9 Protein folding	9,1	56,2	71,8	3,1	1	c
PF#2785	ilvC|Ketol-acid reductoisomerase (Acetohydroxy-acid isomeroreductase) (Alpha-keto-beta-hydroxylacil reductoisomerase)|2.2 Metabolism of amino acids and related molecules	5,0	37,2	226,7	19,2	4	b, c
PF#2789	leuC|3-isopropylmalate dehydratase large subunit (Isopropylmalate isomerase) (Alpha-IPM isomerase) (IPMI)|2.2 Metabolism of amino acids and related molecules	5,4	49,4	133,9	9,7	3	b
PF#2791	nrdJ|Vitamin B12-dependent ribonucleotide reductase (Ribonucleoside-diphosphate reductase NrdJ)|2.3 Metabolism of nucleotides and nucleic acids	4,9	104,8	92,6	2,4	2	a
PF#2806	acn|Aconitase, Aconitate hydratase|2.1.3 TCA cycle	4,9	96,3	1629,4	42,5	31	a, b, c
PF#2818	none|Protein of unknown function|5.2 Protein of unknown function similar to proteins from other organisms	5,3	25,8	55,6	7,8	1	a
PF#2825	aroE|Shikimate 5-dehydrogenase|2.2 Metabolism of amino acids and related molecules	5,0	29,1	57,8	6,6	1	b
PF#2832	sdhA|Succinate dehydrogenase, subunit A|1.4 Membrane bioenergetics (electron transport chain and ATP synthase)	7,1	76,4	106,2	3,3	2	c
PF#2836	moxR|ATPases MoxR family|1.4 Membrane bioenergetics (electron transport chain and ATP synthase)	5,4	37,4	112,2	13,7	3	b
PF#2838	aceE|dehydrogenase E1 component (2-oxo-acid dehydrogenase E1 subunit, homodimeric type)|2.1.2 Main glycolytic pathways	5,5	102,1	267,7	6,4	5	b
PF#2843	||	5,2	48,0	1062,2	43,9	17	a, b
PF#2856	|Zn dependant peptidase|3.10 Protein degradation	5,0	48,5	328,5	22,0	7	a
PF#2869	thrA/hom|Homoserine dehydrogenase|2.2 Metabolism of amino acids and related molecules	5,4	46,5	450,1	22,2	8	a, b
PF#2891	dpm|dolichyl-phosphate beta-D-mannosyltransferase|1.1 Cell wall	5,3	31,7	399,6	31,2	6	b, c
PF#2905	rpoZ|DNA-directed RNA polymerase omega chain (RNAP omega subunit) (Transcriptase omega chain) (RNA polymerase omega subunit)|3.5.3 Transcription elongation	4,8	12,9	52,6	8,5	1	c

PF#2912	alaS|Alanyl-tRNA synthetase (Alanine--tRNA ligase) (AlaRS)|3.7.2 Aminoacyl-tRNA synthetases	5,2	97,0	450,4	15,5	10	a, b, c
PF#2931	slh|S-layer protein precursor|1.1 Cell wall	4,9	57,3	98,0	1,7	2	a, b
PF#2932	|Hypothetical protein|6 Protein of unknown function, without similarity to other proteins	4,7	19,8	649,1	69,1	11	a
PF#2945	|Zinc metallopeptidase|3.10 Protein degradation	4,7	47,5	310,4	17,2	4	b
PF#2947	fhs|Formate--tetrahydrofolate ligase|2.1.1 Specific carbohydrate metabolic pathway	5,2	59,1	732,5	36,7	16	a, b
PF#2955	fusA|Elongation factor G (EF-G)|3.7.4 Translation elongation	4,8	76,6	341,0	13,3	5	b, c
PF#2956	rplC|50S ribosomal protein L3|3.7.1 Ribosomal proteins	10,3	23,6	367,3	26,3	4	c
PF#2958	mer|Coenzyme F420-dependent N5,N10-methylene tetrahydromethanopterin reductase|1.4 Membrane bioenergetics (electron transport chain and ATP synthase)	5,2	44,6	227,5	22,4	6	b
PF#2963	folD|Methylenetetrahydrofolate dehydrogenase (Bifunctional protein)|2.5 Metabolism of coenzymes and prosthetic groups	5,2	30,5	212,3	17,1	5	b
PF#2964	mdh|Malate dehydrogenase|2.1 Metabolism of carbohydrates and related molecules	4,9	34,9	1211,1	60,4	19	a, b, c
PF#2971	|protein of unknown function|5.2 Protein of unknown function similar to proteins from other organisms	4,8	34,9	108,2	5,6	1	b, c
PF#2975	|ABC transporter ATP-binding protein|1.2 Transport/binding proteins and lipoproteins	4,9	62,5	103,7	4,6	2	c
PF#2979	valS|Valyl-tRNA synthetase (Valine--tRNA ligase) (ValRS)|3.7.2 Aminoacyl-tRNA synthetases	5,1	97,5	154,8	3,4	3	b
PF#3002	fabF (fabB)|3-oxoacyl-[acyl-carrier-protein] synthase (Beta-ketoacyl-ACP synthase)|2.4 Metabolism of lipids	5,1	43,7	124,0	11,7	2	b, c
PF#3003	fabH|3-oxoacyl-(Acyl-carrier-protein) synthase III|2.4 Metabolism of lipids	4,8	31,7	82,7	3,3	1	c
PF#3004	pf3004|Carboxylic ester hydrolase|2.4 Metabolism of lipids	5,2	32,9	230,0	10,7	2	b, c
PF#3019	glgP|Phosphorylase (alpha-glucan phosphorylase)|2.1 Metabolism of carbohydrates and related molecules	5,2	94,6	354,3	9,7	5	b
PF#3022	pf3022|Homoserine O-acetyltransferase|2.2 Metabolism of amino acids and related molecules	5,8	44,3	197,0	12,8	4	b
PF#3034	hisS|Histidyl-tRNA synthetase (Histidine--tRNA ligase) (HisRS)|3.7.2 Aminoacyl-tRNA synthetases	5,3	48,9	220,7	11,5	4	b
PF#3039	trpB|Tryptophan synthase beta chain (TrpB)|2.2 Metabolism of amino acids and related molecules	5,3	44,7	54,7	2,9	1	b
PF#3040	glnS|Glutaminyl-tRNA synthetase|3.7.2 Aminoacyl-tRNA synthetases	5,2	64,2	219,9	9,1	4	b
PF#3042	|ABC transporter substrate-binding protein|1.2 Transport/binding proteins and lipoproteins	5,0	33,5	335,1	35,9	6	a
PF#3053	ddlA|D-alanine--D-alanine ligase (D-alanylalanine synthetase)|1.1 Cell wall	4,9	40,4	258,6	16,5	5	b, c
PF#3055	ilvE (bcaT)|Branched-chain amino acid aminotransferase |2.2 Metabolism of amino acids and related molecules	5,0	39,8	292,2	19,9	6	b
PF#3068	pnpA|Polyribonucleotide nucleotidyltransferase (Polynucleotide phosphorylase) (PNPase) (Guanosine pentaphosphate synthetase)|2.3 Metabolism of nucleotides and nucleic acids	4,8	79,4	166,9	8,2	3	b, c
PF#3069	glpK|Glycerol kinase (ATP:glycerol 3-phosphotransferase) (Glycerokinase) (GK) |2.1.1 Specific carbohydrate metabolic pathway	4,7	55,7	329,6	14,2	5	b
PF#3074	rpsA|30S ribosomal protein S1|3.7.1 Ribosomal proteins	4,6	53,6	754,1	40,5	15	a, b, c
PF#3082	||	8,9	78,6	588,7	16,1	8	a
PF#3083	aroH|Phospho-2-dehydro-3-deoxyheptonate aldolase|2.2 Metabolism of amino acids and related molecules	5,2	51,1	336,0	13,9	5	b
PF#3085	sucB|Dihydrolipoamide acyltransferase, E2 component of 2-oxoacid dehydrogenase complex|2.1 Metabolism of carbohydrates and related molecules	4,6	59,6	207,5	9,5	3	b, c
PF#3088	dapA|Dihydrodipicolinate synthase|2.2 Metabolism of amino acids and related molecules	5,0	29,4	339,5	31,0	6	a, b, c
PF#3090	glgC|Glucose-1-phosphate adenylyltransferase (ADP-glucose synthase) (ADP-glucose pyrophosphorylase) (ADPGlc PPase)|2.1.1 Specific carbohydrate metabolic pathway	4,9	45,0	157,9	10,8	3	b
PF#3106	slpA|surface layer protein A (S-layer protein A)|1.1 Cell wall	4,7	57,0	2593,7	67,8	39	a, b
PF#3107	glyA|Glycine hydroxymethyltransferase precursor|2.2 Metabolism of amino acids and related molecules	5,3	51,9	1187,2	64,3	22	a

PF#3109	hemL|Glutamate-1-semialdehyde 2,1-aminomutase (GSA) (Glutamate-1-semialdehyde aminotransferase)(GSA-AT)|2.5 Metabolism of coenzymes and prosthetic groups	5,1	45,9	332,8	14,3	4	b
PF#3122	mmsB|3-hydroxyisobutyrate dehydrogenase related beta-hydroxyacid dehydrogenase|2.4 Metabolism of lipids	4,6	28,1	268,1	22,6	4	b
PF#3134	nirA2/sir2|Sulfite reductase [ferredoxin]|2.7 Metabolism of sulfur	5,9	68,0	236,7	13,2	5	b, c
PF#3145	hsp20 1|Heat shock protein 20 1 (20 kDa chaperone 1)|3.9 Protein folding	4,7	17,2	448,4	62,3	8	a, b, c
PF#3161	||	4,2	16,5	88,9	7,5	1	a
PF#3174	lspA|large surface protein A|1.1 Cell wall	4,8	137,3	2461,5	48,1	37	a, b
PF#3204	nifJ|Pyruvate-flavodoxin oxidoreductase|1.4 Membrane bioenergetics (electron transport chain and ATP synthase)	5,2	136,6	2636,5	54,3	47	a, b, c
PF#3205	|Dihydroorotate dehydrogenase|2.3 Metabolism of nucleotides and nucleic acids	4,8	40,7	191,5	13,8	3	b, c
PF#3211	none|Protein of unknown function|5.2 Protein of unknown function similar to proteins from other organisms	6,4	25,5	141,7	13,9	4	a
PF#3216	rpsF|30S ribosomal protein S6|3.7.1 Ribosomal proteins	5,4	12,0	249,4	43,3	4	b, c
PF#3271	fumC|Fumarate hydratase, class-II|2.1.3 TCA cycle	5,3	51,1	2175,1	74,9	38	a, b, c
PF#3341	dhaK|DhaK PTS-dependent dihydroxyacetone kinase, dihydroxyacetone-binding subunit |2.1.1 Specific carbohydrate metabolic pathway	4,7	34,7	163,0	11,2	3	b
PF#3363	Arth_4141|FAD linked oxidase domain protein|1.4 Membrane bioenergetics (electron transport chain and ATP synthase)	5,7	100,5	214,0	5,4	4	b
PF#3403	ppgK|Polyphosphate glucokinase|2.1.2 Main glycolytic pathways	6,0	27,4	134,7	15,7	3	b
PF#3408	rpsE|30S ribosomal protein S5|3.7.1 Ribosomal proteins	10,0	24,0	192,8	18,1	3	c
PF#3411	lysA|Diaminopimelate decarboxylase (DAP decarboxylase)|2.2 Metabolism of amino acids and related molecules	5,9	59,0	336,7	14,5	7	a
PF#3412	|Hypothetical protein|6 Protein of unknown function, without similarity to other proteins	6,5	30,9	360,4	20,2	5	a
PF#3415	hisC|Histidinol-phosphate aminotransferase (Imidazole acetol- phosphate transaminase)|2.2 Metabolism of amino acids and related molecules	6,0	44,4	250,5	17,4	6	b
PF#3426	ansA|L-asparaginase I|2.2 Metabolism of amino acids and related molecules	4,7	37,3	208,8	16,3	4	b
PF#3427	FimP|Type-1 fimbrial major subunit precursor|1.1 Cell wall	4,7	36,9	110,8	10,3	2	a
