# Supplementary material for: The Complete Genome of Propionibacterium freudenreichii CIRM-BIA1T, a Hardy Actinobacterium with Food and Probiotic Applications
Source: PLoS One. 2010 Jul 23;5(7):e11748. doi: 10.1371/journal.pone.0011748 (PMC2909200; doi:10.1371/journal.pone.0011748)
Supplement: Table S2 — Insertion sequences occurence. (0.00 MB PDF) [file pone.0011748.s002.pdf]

IS Name	Family name (sub-group)	N° of complete copies	N° of partial copies
IS*Pfr1*	IS*L3*	10	0
IS*Pfr2*	IS*L3*	1	0
IS*Pfr3*	IS*L3*	4	2
IS*Pfr4*	IS*L3*	1	0
IS*Pfr5*	IS*481*	2	0
IS*Pfr6*	IS*L3*	3	0
IS*Pfr7*	IS*L3*	1	0
IS*Pfr8*	IS*L3*	3	0
IS*Pfr9*	IS*30*	2	0
IS*Pfr10*	IS*3* (IS*407*)	8	1
IS*Pfr11*	IS*3* (IS*3*)	5	0
IS*Pfr12*	IS*3* (IS*51*)	6	0
IS*Pfr13*	IS*3* (IS*51*)	1	0
IS*Pfr14*	IS*30*	1	0
IS*Pfr15*	IS*481*	2	0
IS*Pfr16*	IS*30*	1	0
IS*Pfr17*	IS*481*	1	1
IS*Pfr18*	IS*L3*	1	0
IS*Pfr19*	IS*481*	2	1
IS*Pfr20*	IS*L3*	1	5
IS*Pfr21*	IS*481*	1	0
