# Supplementary material for: The Complete Genome of Propionibacterium freudenreichii CIRM-BIA1T, a Hardy Actinobacterium with Food and Probiotic Applications
Source: PLoS One. 2010 Jul 23;5(7):e11748. doi: 10.1371/journal.pone.0011748 (PMC2909200; doi:10.1371/journal.pone.0011748)
Supplement: Table S3 — Bidirectional best hits results between P. freudenreichii proteins and P. acnes proteins. No P. acnes protein putatively involved in the degradation of host molecules or in the mediation of inflammation was found in P. freudenreichii. (0.15 MB PDF) [file pone.0011748.s003.pdf]

locus tag on *P. freudenreichii*	gene name on *P. freudenreichii*	product	locus tag on *P. acnes*	SurfG+ prediction
PFREUD_00010	dnaA	Chromosomal replication initiator protein	PPA0001	CYT
PFREUD_00090	recF	DNA replication and repair protein recF	PPA0004	CYT
PFREUD_00150	gyrB2	DNA gyrase subunit B	PPA0009	CYT
PFREUD_00160	gyrA	DNA gyrase subunit A	PPA0010	CYT
PFREUD_00170		Hypothetical protein	PPA1806	CYT
PFREUD_00290	iol	myo-inositol 2-dehydrogenase	PPA0466	CYT
PFREUD_00370	ald	Alanine dehydrogenase	PPA2274	CYT
PFREUD_00390		ErfK/YbiS/YcfS/YnhG precursor	PPA2270	CYT
PFREUD_00410		Hypothetical protein	PPA1095	CYT
PFREUD_00470	rmlA (rfbA)	Glucose-1-phosphate thymidylyltransferase	PPA2288	CYT
PFREUD_00670		1-acyl-sn-glycerol-3-phosphate acyltransferase	PPA2237	CYT
PFREUD_00680		dehydrogenase	PPA2236	CYT
PFREUD_00770	gpsA	Glycerol-3-phosphate dehydrogenase [NAD(P)+] (NAD(P)H- dependent glycerol-3-phosphate dehydrogenase)	PPA2315	CYT
PFREUD_00820		two-component response regulator	PPA2112	CYT
PFREUD_00840	ptpA	Low molecular weight protein-tyrosine- phosphatase (protein-tyrosine-phosphatase)	PPA2110	CYT
PFREUD_00860	argF	Ornithine carbamoyltransferase	PPA0584	CYT
PFREUD_01060	aatB	Leucyl/phenylalanyl-tRNA-protein transferase	PPA2221	CYT
PFREUD_01100		Hypothetical protein	PPA2088	CYT
PFREUD_01130	alr	Alanine racemase	PPA2285	CYT
PFREUD_01350	serS1	Seryl-tRNA synthetase	PPA2219	CYT
PFREUD_01430	tpx	thiol peroxidase	PPA0590	CYT
PFREUD_01440	npdA	Silent information regulator protein Sir2 /NAD- dependent deacetylase	PPA2172	CYT
PFREUD_01460	argG	Argininosuccinate synthase	PPA2201	CYT
PFREUD_01470		NADPH:quinone reductase and related Zn-dependent oxidoreductases	PPA2200	CYT
PFREUD_01560		Short chain dehydrogenase	PPA1711	CYT
PFREUD_01580	aroE	Shikimate 5-dehydrogenase	PPA1181	CYT
PFREUD_01630		two-component response regulator LuxR	PPA2028	CYT
PFREUD_01690	fkbP	FK506-binding protein (peptidyl-prolyl cis-trans isomerase)	PPA2163	CYT
PFREUD_01810		Hypothetical protein	PPA0164	CYT
PFREUD_01830		FAD-dependent pyridine nucleotide-disulphide oxidoreductase:4Fe-4S ferredoxin, iron-sulfur binding:Aromatic ring hydroxylase	PPA0163	CYT
PFREUD_01840	nifJ1	Pyruvate synthase/Pyruvate-flavodoxin oxidoreductase	PPA0162	CYT
PFREUD_01850		Dihydroorotate dehydrogenase	PPA0161	CYT
PFREUD_02140		Hypothetical protein	PPA1479	CYT
PFREUD_02170	lysS	Lysyl-tRNA synthetase	PPA0181	CYT
PFREUD_02190	bkdA1	2-oxoisovalerate dehydrogenase subunit alpha (Branched- chain alpha-keto acid dehydrogenase E1 component alpha chain) (BCKDH E1-alpha)	PPA2094	CYT
PFREUD_02200	bkdA2	2-oxoisovalerate dehydrogenase subunit beta (EC 1.2.4.4) (Branched- chain alpha-keto acid dehydrogenase E1 component beta chain) (BCKDH E1-beta) Pyruvate dehydrogenase E1 component subunit beta	PPA2093	CYT
PFREUD_02210	bkdB	dihydrolipoyllysine-residue (2- methylpropanoyl)transferase. Lipoamide acyltransferase component of branched-chain alpha-keto acid dehydrogenase complex (E2) (Dihydrolipoamide branched chain transacylase)	PPA2092	CYT
PFREUD_02220		Hypothetical protein	PPA1999	CYT
PFREUD_02240		Hypothetical protein	PPA0183	CYT
PFREUD_02270		Hypothetical protein	PPA0120	CYT
PFREUD_02290		Hypothetical secreted protein	PPA2334	CYT
PFREUD_02370	lacZ	Beta-galactosidase (Lactase) LacZ	PPA1700	CYT
PFREUD_02390		Transcriptional regulator	PPA1140	CYT
PFREUD_02420	caiA	Crotonobetainyl-CoA dehydrogenase (Crotonobetainyl-CoA reductase)	PPA2216	CYT
PFREUD_02480	fixA (ydiQ)	Electron transfer flavoprotein (FixA protein)	PPA2214	CYT
PFREUD_02490	fixB (ydiR)	Electron transfer flavoprotein, carnitine metabolism (FixB protein)	PPA2213	CYT
PFREUD_02500	fixC (ydiS)	Electron transfer flavoprotein-quinone oxidoreductase (FixC protein)	PPA2212	CYT
PFREUD_02520		DNA or RNA helicase	PPA0896	CYT
PFREUD_02540	udgA (rkpK)	UDP-glucose 6-dehydrogenase	PPA0593	CYT
PFREUD_02550	argS	Arginyl-tRNA synthetase (Arginine--tRNA ligase) (ArgRS)	PPA2128	CYT
PFREUD_02560	fepC3	FepC	PPA0792	CYT
PFREUD_02870	dps	Starvation-inducible DNA-binding protein	PPA2134	CYT
PFREUD_03030		Hypothetical protein	PPA0424	CYT
PFREUD_03100	kduD	2-deoxy-D-gluconate 3-dehydrogenase	PPA0372	CYT
PFREUD_03110	cat	Coenzyme A transferase (Putative succinyl-CoA or butyryl-CoA:coenzyme A transferase)	PPA2053	CYT
PFREUD_03160	PF2369	Putative aldo/keto reductase (oxidoreductase)	PPA2129	CYT
PFREUD_03180	rsmC	rRNA (guanine-N2-)-methyltransferase	PPA2050	CYT
PFREUD_03210		Hypothetical protein	PPA2049	CYT
PFREUD_03230	ppdk	Pyruvate phosphate dikinase	PPA2048	CYT
PFREUD_03250	rbsK	carbohydrate or pyrimidine kinases PfkB family	PPA0018	CYT
PFREUD_03270		two component system response regulator	PPA2043	CYT
PFREUD_03290	metN	Methionine import ATP-binding protein metN	PPA1759	CYT
PFREUD_03380	thiM	Hydroxyethylthiazole kinase	PPA0885	CYT
PFREUD_03610		Zinc-containing alcohol dehydrogenase	PPA2245	CYT
PFREUD_03760		Hemerythrin HHE cation binding region	PPA0240	CYT
PFREUD_03830		Hypothetical protein	PPA0205	CYT
PFREUD_03840	recR	Recombination protein recR	PPA0206	CYT
PFREUD_03860	ileS	Isoleucyl-tRNA synthetase (Isoleucine--tRNA ligase)	PPA0216	CYT
PFREUD_03870		oxidoreductase	PPA0219	CYT
PFREUD_03880	ask	Aspartokinase (Aspartate kinase)	PPA2148	CYT
PFREUD_04000	purD	Phosphoribosylamine-glycine ligase	PPA1993	CYT
PFREUD_04010	purB	Adenylosuccinate lyase	PPA1992	CYT
PFREUD_04050	purL/purQ	Phosphoribosylformylglycinamidine synthase 1 (Phosphoribosylformylglycinamidine synthase I) (FGAM synthase I)	PPA1987	CYT
PFREUD_04250	pepB	Phosphatidylethanolamine-binding protein	PPA2138	CYT
PFREUD_04260		DeaD/DeaH box helicase	PPA2135	CYT
PFREUD_04270	eda, hga, kdgA	2-dehydro-3-deoxyphosphogluconate aldolase/4- hydroxy-2-oxoglutarate aldolase	PPA2133	CYT
PFREUD_04290	pgi, PPA2131	Glucose-6-phosphate isomerase (EC 5.3.1.9) (GPI) (Phosphoglucose isomerase) (PGI) (Phosphohexose isomerase) (PHI)	PPA2131	CYT
PFREUD_04320	Arth_4141	FAD linked oxidase domain protein	PPA0775	CYT
PFREUD_04430		Thiamine pyrophosphate enzyme	PPA1468	CYT
PFREUD_04540	hrpA1	ATP-dependent helicase HrpA	PPA0155	CYT
PFREUD_04550	cobA	CobA Uroporphyrinogen III methyltransferase	PPA0439	CYT
PFREUD_04560	cbiO1	Cobalt import ATP-binding protein CbiO	PPA0434	CYT

PFREUD_04600	gntR	transcription factor (transcription regulation)	PPA0740	CYT
PFREUD_04650	dnaJ2	Chaperone protein dnaJ 2 (DnaJ2 protein) (Heat shock protein 40 2)	PPA2038	CYT
PFREUD_04660	hspr2	Heat shock protein transcriptional repressor HspR2 (Hspr2 protein)	PPA2037	CYT
PFREUD_04670		BadF/BadG/BcrA/BcrD ATPase family protein	PPA1986	CYT
PFREUD_04680	purL	Phosphoribosylformylglycinamidine synthase II (FGAM synthase II)	PPA1977	CYT
PFREUD_04690		Zinc metallopeptidase	PPA1972	CYT
PFREUD_04720	fhs	Formate--tetrahydrofolate ligase	PPA0049	CYT
PFREUD_04770	purF	Amidophosphoribosyltransferase	PPA1970	CYT
PFREUD_04780	purM	Phosphoribosylformylglycinamidine cyclo-ligase	PPA1969	CYT
PFREUD_04810	pf962	Carboxylic ester hydrolase	PPA1967	CYT
PFREUD_05030		Deoxycytidylate deaminase (dCMP deaminase)	PPA1950	CYT
PFREUD_05090		nuclease (RecB family)	PPA1944	CYT
PFREUD_05110		ABC transporter ATP-binding protein	PPA1942	CYT
PFREUD_05130	dhbC	Menaquinone-specific isochorismate synthase	PPA1940	CYT
PFREUD_05140		electron transfer oxidoreductase	PPA1938	CYT
PFREUD_05170	nuoB	NADH-quinone oxidoreductase chain B	PPA1935	CYT
PFREUD_05180	nuoC	NADH-quinone oxidoreductase chain C (NADH dehydrogenase I, chain C)	PPA1934	CYT
PFREUD_05190	nuoD	NADH-quinone oxidoreductase chain D (EC 1.6.99.5) (NADH dehydrogenase I, chain D)	PPA1933	CYT
PFREUD_05200	nuoE	NADH-quinone oxidoreductase chain E	PPA1932	CYT
PFREUD_05210	nuoF	NADH-quinone oxidoreductase chain F (NADH dehydrogenase I, chain F) (NDH-1, chain F)	PPA1931	CYT
PFREUD_05220	nuoG	NADH-quinone oxidoreductase chain G (NADH dehydrogenase I, chain G)	PPA1930	CYT
PFREUD_05240	nuoI	NADH-quinone oxidoreductase subunit I (NADH dehydrogenase I subunit I) (NDH-1 subunit I)	PPA1928	CYT
PFREUD_05300	idsA	Heptaprenyl diphosphate synthase component II	PPA1921	CYT
PFREUD_05330		Metalloprotease (Peptidase family M13)	PPA1909	CYT
PFREUD_05340		Thiamine pyrophosphate (TPP family)	PPA1905	CYT
PFREUD_05350		Pyruvate flavodoxin/ferredoxin oxidoreductase	PPA1904	CYT
PFREUD_05380	xthA	Exodeoxyribonuclease III/exonuclease III	PPA1898	CYT
PFREUD_05410	ahpC	Peroxiredoxin/Alkyl hydroperoxide reductase subunit C /Thioredoxin peroxidase/Alkyl hydroperoxide reductase protein C22/General stress protein 22	PPA1989	CYT
PFREUD_05420	ahpF	Alkyl hydroperoxide reductase subunit F	PPA1990	CYT
PFREUD_05450	rpmG	50S ribosomal protein L33 RpmG	PPA1897	CYT
PFREUD_05460		R_hydratase_like, (R)-hydratase [(R)-specific enoyl-CoA hydratase].	PPA1896	CYT
PFREUD_05470		R_hydratase_like, (R)-hydratase [(R)-specific enoyl-CoA hydratase]	PPA1895	CYT
PFREUD_05480	murB	UDP-N-acetylenolpyruvoylglucosamine reductase (UDP-N- acetylmuramate dehydrogenase)	PPA1894	CYT
PFREUD_05520	nusG	Transcription antitermination protein NusG	PPA1891	CYT
PFREUD_05530	rplK	50S ribosomal protein L11	PPA1890	CYT
PFREUD_05540	rplA	50S ribosomal protein L1	PPA1889	CYT
PFREUD_05550	lipB	Lipoyltransferase (Lipoyl-[acyl-carrier-protein]- protein- N-lipoyltransferase) (Lipoate-protein ligase B)	PPA0692	CYT
PFREUD_05560	lipA	Lipoic acid synthetase	PPA0689	CYT
PFREUD_05570	rplJ	50S ribosomal protein L10	PPA1887	CYT
PFREUD_05580	rplL	50S ribosomal protein L7/L12	PPA1885	CYT
PFREUD_05600	rpoB	DNA-directed RNA polymerase beta chain (RNAP beta subunit) (Transcriptase beta chain) (RNA polymerase subunit beta)	PPA1884	CYT
PFREUD_05610	rpoC	DNA-directed RNA polymerase beta chain (RNAP beta subunit) (Transcriptase beta chain) (RNA polymerase beta subunit)	PPA1883	CYT
PFREUD_05620	rpsL	30S ribosomal protein S12	PPA1878	CYT
PFREUD_05630	rpsG	30S ribosomal protein S7	PPA1876	CYT
PFREUD_05640	fusA	Elongation factor G (EF-G)	PPA1875	CYT
PFREUD_05650	tuf	Elongation factor Tu	PPA1873	CYT
PFREUD_05680	gltX	Glutamyl-tRNA synthetase (Glutamate--tRNA ligase) (GluRS)	PPA1869	CYT
PFREUD_05690	pabC	Branched-chain amino acid aminotransferase/aminodeoxychorismate lyase	PPA2261	CYT
PFREUD_05700	rpsJ	30S ribosomal protein S10	PPA1865	CYT
PFREUD_05710	rplC	50S ribosomal protein L3	PPA1863	CYT
PFREUD_05730	rplW	50S ribosomal protein L23	PPA1861	CYT
PFREUD_05740	rplB	50S ribosomal protein L2	PPA1860	CYT
PFREUD_05750	rpsS	30S ribosomal protein S19	PPA1859	CYT
PFREUD_05780	rplP	50S ribosomal protein L16	PPA1856	CYT
PFREUD_05790	rpmC	50S ribosomal protein L29	PPA1855	CYT
PFREUD_05800	rpsQ	30S ribosomal protein S17	PPA1853	CYT
PFREUD_05810	rplN	50S ribosomal protein L14	PPA1852	CYT
PFREUD_05820	rplX	50S ribosomal protein L24	PPA1851	CYT
PFREUD_05830	rplE	50S ribosomal protein L5	PPA1850	CYT
PFREUD_05840	rpsN1, rpsZ	30S ribosomal protein S14 type Z	PPA1849	CYT
PFREUD_05850	rpsH	30S ribosomal protein S8	PPA1848	CYT
PFREUD_05860	rplF	50S ribosomal protein L6	PPA1846	CYT
PFREUD_05870	rplR	Ribosomal protein L18	PPA1845	CYT
PFREUD_05890	rpmD	50S ribosomal protein L30	PPA1843	CYT
PFREUD_05900	rplO	50S ribosomal protein L15	PPA1842	CYT
PFREUD_05940		ABC transporter, ATPase subunit	PPA2066	CYT
PFREUD_05980	adk	Adenylate kinase (EC 2.7.4.3) (ATP-AMP transphosphorylase)	PPA1834	CYT
PFREUD_05990	map	Methionine aminopeptidase (MAP) (Peptidase M)	PPA1833	CYT
PFREUD_06010		Hypothetical protein	PPA2117	CYT
PFREUD_06020	infA	Translation initiation factor IF-1	PPA1832	CYT
PFREUD_06030		Hypothetical protein	PPA1831	CYT
PFREUD_06040	rpsM	30S ribosomal protein S13	PPA1829	CYT
PFREUD_06050	rpsK	30S ribosomal protein S11	PPA1828	CYT
PFREUD_06060	rpsD	30S ribosomal protein S4	PPA1827	CYT
PFREUD_06070	rpoA	DNA-directed RNA polymerase alpha chain (RNAP alpha subunit) (Transcriptase alpha chain) (RNA polymerase subunit alpha)	PPA1826	CYT
PFREUD_06090	gtfE	Glycosyltransferase	PPA1824	CYT
PFREUD_06110	sodA	Iron/Manganese superoxide dismutase (Superoxide dismutase [Mn/Fe]) (SODM)	PPA1818	CYT
PFREUD_06120		ABC transporter ATP-binding protein	PPA1817	CYT
PFREUD_06150	cbiO2	ABC transporter, ATP-binding protein, cobalt	PPA1813	CYT
PFREUD_06190		methyltransferase	PPA1810	CYT
PFREUD_06200	truA	tRNA pseudouridine synthase A (tRNA-uridine isomerase I) (tRNA pseudouridylate synthase I)	PPA1809	CYT
PFREUD_06240	rplM	50S ribosomal protein L13	PPA1803	CYT
PFREUD_06250	rspI	30S ribosomal protein S9	PPA1802	CYT
PFREUD_06260	glmM	Phosphoglucosamine mutase	PPA1801	CYT
PFREUD_06270	coaA	Pantothenate kinase (Pantothenic acid kinase)	PPA1799	CYT
PFREUD_06280	glmS	Glucosamine--fructose-6-phosphate aminotransferase (Hexosephosphate aminotransferase, D- fructose-6-phosphate amidotransferase)	PPA1794	CYT
PFREUD_06300		Carbohydrate kinase	PPA1792	CYT
PFREUD_06310	sqdX (rfaG)	Glycosyltransferase	PPA1791	CYT

PFREUD_06340		Peptidase, family M22	PPA1784	CYT
PFREUD_06350	rimI	Ribosomal-protein-alanine acetyltransferase	PPA1783	CYT
PFREUD_06360	gcp	Putative O-sialoglycoprotein endopeptidase	PPA1782	CYT
PFREUD_06370	bluB/cobT2	Phosphoribosyltransferase/nitroreductase (fusion gene) (Nicotinate-nucleotide-dimethylbenzimidazole phosphoribosyltransferase)	PPA0953	CYT
PFREUD_06390	mutT	,8-dihydro-8-oxoguanine-triphosphatase (Mutator MutT protein/7,8-dihydro-8-oxoguanine-triphosphatase)	PPA1781	CYT
PFREUD_06410		Hypothetical protein	PPA1776	CYT
PFREUD_06460	groS1(groES1) (hsp10)	10 kDa chaperonin 1 (Protein Cpn10 1) (groES protein 1) (Heat shock 10 1)	PPA1773	CYT
PFREUD_06470	groL1 (groEL1)	60 kDa chaperonin 1 (Protein Cpn60 1) (groEL protein 1) (Heat shock protein 60 1	PPA1772	CYT
PFREUD_06480	guaB1	Inosine-5 -monophosphate dehydrogenase (IMP dehydrogenase) (IMPDH) (IMPD) / GMP reductase)	PPA1768	CYT
PFREUD_06490	guaB2	Inosine-5 -monophosphate dehydrogenase (IMP dehydrogenase) (IMPDH) (IMPD) / GMP reductase	PPA1767	CYT
PFREUD_06550	cysE	Serine acetyltransferase	PPA1765	CYT
PFREUD_06560	cys1	Cysteine synthase 1	PPA0963	CYT
PFREUD_06590	araD1	L-ribulose-5-phosphate 4-epimerase	PPA0882	CYT
PFREUD_06620	lacI1	arabinose operon repressor	PPA0088	CYT
PFREUD_06640		ATP binding protein of ABC transporter , ATPase component	PPA0015	CYT
PFREUD_06680	guaA	GMP synthase [glutamine-hydrolyzing] (Glutamine amidotransferase) (GMP synthetase)	PPA1764	CYT
PFREUD_06800	pcrA	ATP-dependent DNA helicase	PPA1756	CYT
PFREUD_06820	purN	5 -phosphoribosylglycinamide formyltransferase (phosphoribosylglycinamide formyltransferase)	PPA1748	CYT
PFREUD_06830	purH	Phosphoribosylaminoimidazolecarboxamide formyltransferase (AICAR transformylase)	PPA1747	CYT
PFREUD_06840	folD	Methylenetetrahydrofolate dehydrogenase (Bifunctional protein)	PPA1743	CYT
PFREUD_06860	mdh	Malate dehydrogenase	PPA1740	CYT
PFREUD_06910		Hypothetical protein	PPA1735	CYT
PFREUD_06930	rnhA	Ribonuclease H	PPA1729	CYT
PFREUD_06950	caiD	Enoyl-CoA hydratase/carnithine racemase CaiD	PPA1899	CYT
PFREUD_06960	metG	Methionyl-tRNA synthetase (Methionine--tRNA ligase) (MetRS)	PPA1728	CYT
PFREUD_07050		zinc-binding dehydrogenase	PPA2035	CYT
PFREUD_07100	accA	Acetyl-CoA carboxylase	PPA1719	CYT
PFREUD_07110	mutM2	Formamidopyrimidine-DNA glycosylase (DNA- formamidopyrimidine glycosylase)	PPA1623	CYT
PFREUD_07150	maf	Maf-like protein	PPA1709	CYT
PFREUD_07170	pccB	Propionyl-CoA carboxylase beta chain	PPA1707	CYT
PFREUD_07250	birA	BirA, Biotin-(acetyl-CoA carboxylase) ligase	PPA1705	CYT
PFREUD_07290		Peptidase S51 family protein, dipeptidase E	PPA1704	CYT
PFREUD_07300	purK	Phosphoribosylaminoimidazole carboxylase ATPase subunit	PPA1702	CYT
PFREUD_07330	manC	Mannose-1-phosphate guanylyltransferase	PPA1696	CYT
PFREUD_07350	whiB1	Transcription factor WhiB	PPA1694	CYT
PFREUD_07380		Hypothetical protein	PPA1691	CYT
PFREUD_07400	pmm	Phosphomannomutase (PMM)	PPA2023	CYT
PFREUD_07420		Hypothetical protein	PPA1688	CYT
PFREUD_07470	malQ1	4-alpha-glucanotransferase (Amylomaltase) (Disproportionating enzyme) (D-enzyme)	PPA1683	CYT
PFREUD_07480	menC	O-succinylbenzoate-CoA synthase (4-(2'- carboxyphenyl)-4-oxybutyric acid synthase)	PPA0902	CYT
PFREUD_07490	menD	Menaquinone biosynthesis protein	PPA0903	CYT
PFREUD_07520	menF	Isochorismate synthetase (enterochelin biosynthesis)	PPA0904	CYT
PFREUD_07530	menE2	O-succinylbenzoic acid-CoA ligase (2- succinylbenzoate--CoA ligase)	PPA0906	CYT
PFREUD_07540	menB	Naphthoate synthase (Dihydroxynaphthoic acid synthetase)	PPA0907	CYT
PFREUD_07550	pncB	Nicotinate phosphoribosyltransferase	PPA1681	CYT
PFREUD_07580	murI	Glutamate racemase	PPA1675	CYT
PFREUD_07590	rph	Ribonuclease PH (RNase PH) (tRNA nucleotidyltransferase)	PPA1674	CYT
PFREUD_07610	thyA	Thymidylate synthase	PPA1672	CYT
PFREUD_07630	dinB	Nucleotidyltransferase/DNA polymerase involved in DNA repair (DNA polymerase IV)	PPA1670	CYT
PFREUD_07640	argK	Kinase ArgK	PPA0597	CYT
PFREUD_07650	mutB	Methylmalonyl-CoA mutase large subunit (Methylmalonyl-CoA mutase alpha subunit) (MCM-alpha) (MUTB- (R)-2-Methyl-3-oxopropanoyl-CoA CoA-carbonylmutase)	PPA0596	CYT
PFREUD_07660	mutA	Methylmalonyl-CoA mutase small subunit (Methylmalonyl-CoA mutase beta subunit) (MCB-beta)	PPA0595	CYT
PFREUD_07680	cbiL	CbiL Precorrin-2 C20-methyltransferase	PPA0420	CYT
PFREUD_07690	cbiF	CbiF Precorrin-4 C11-methyltransferase	PPA0421	CYT
PFREUD_07700	cobJ/cbiE/cbiG/cbiH	precorrin methylase (precorrin-3B C17- methyltransferase) CbiE/G/H fusion protein	PPA0422	CYT
PFREUD_07710	cysG/cbiX	CysG/CbiX	PPA0423	CYT
PFREUD_07730	cbiC	Precorrin 8X methylmutase CbiC	PPA0429	CYT
PFREUD_07750	cbiT	Precorrin 8 decarboxylase CbiT	PPA0436	CYT
PFREUD_07790	bcp	Bcp Putative peroxiredoxin (Thioredoxin reductase)	PPA1661	CYT
PFREUD_07830	cobB/cobQ	(glutamine amidotransferase) (adenosylcobyric acid synthase (glutamine-hydrolysing))	PPA1654	CYT
PFREUD_07840		UDP-N-acetylmuramyl tripeptide synthase (Mur ligase)	PPA1653	CYT
PFREUD_07890	orn	Oligoribonuclease	PPA1642	CYT
PFREUD_07930		ABC transporter	PPA1639	CYT
PFREUD_07960	ssb2	Single-stranded DNA-binding protein 2 (SSB 2) (Helix-destabilizing protein 2)	PPA1637	CYT
PFREUD_07970		ABC transporter ATP-binding protein	PPA1636	CYT
PFREUD_07980	dhaK	DhaK PTS-dependent dihydroxyacetone kinase, dihydroxyacetone-binding subunit	PPA1947	CYT
PFREUD_07990	dhaL	PTS-dependent dihydroxyacetone kinase, ADP- binding subunit DhaL	PPA1948	CYT
PFREUD_08010	galT	Galactose-1-phosphate uridylyltransferase (UDP- glucose---hexose-1-phosphate uridylyltransferase)	PPA2218	CYT
PFREUD_08040		oxidoreductase	PPA1634	CYT
PFREUD_08050	marR1	Transcriptional regulator, MarR family	PPA1633	CYT
PFREUD_08060		long-chain fatty-acid CoA ligase (AMP-binding enzyme) (long-chain-fatty-acid---CoA ligase)	PPA1632	CYT
PFREUD_08070	pf2652	Hypothetical protein	PPA1631	CYT
PFREUD_08080	thiD2	hydroxymethylpyrimidine/phosphomethylpyrimidinekinase	PPA1712	CYT
PFREUD_08090	gnd2	6-phosphogluconate dehydrogenase, decarboxylating	PPA1629	CYT
PFREUD_08100	pepN	Aminopeptidase N , Lysyl aminopeptidase	PPA1627	CYT
PFREUD_08130	ligA	DNA ligase (NAD+)	PPA1626	CYT
PFREUD_08210	tig	Trigger factor (TF)	PPA1575	CYT
PFREUD_08240	clpP1	ATP-dependent Clp protease proteolytic subunit 1 (Endopeptidase Clp 1)	PPA1573	CYT
PFREUD_08250	clpP2	ATP-dependent Clp protease proteolytic subunit 2 (Endopeptidase Clp 2)	PPA1572	CYT
PFREUD_08260	clpX	ATP-dependent Clp protease (ATP-dependent Clp protease, ATP-binding subunit ClpX)	PPA1571	CYT
PFREUD_08290	pfkA	6-phosphofructokinase	PPA0090	CYT
PFREUD_08310	valS	Valyl-tRNA synthetase (Valine--tRNA ligase) (ValRS)	PPA1568	CYT
PFREUD_08350	folC	Folylpolyglutamate synthase (bifunctionnal enzyme)	PPA0821	CYT
PFREUD_08360	ndk	Nucleoside-diphosphate kinase	PPA0823	CYT
PFREUD_08380		Fe-S oxidoreductases family 2	PPA0824	CYT
PFREUD_08420		Hypothetical protein	PPA0825	CYT
PFREUD_08450	rplU	50S ribosomal protein L21	PPA0827	CYT
PFREUD_08460	rpmA	50S ribosomal protein L27	PPA0829	CYT
PFREUD_08480	obg	GTPase	PPA0833	CYT

PFREUD_08490	proB	Glutamate 5-kinase (Gamma-glutamyl kinase) (GK)	PPA0834	CYT
PFREUD_08510	proA	Gamma-glutamyl phosphate reductase (GPR) (Glutamate-5- semialdehyde dehydrogenase) (Glutamyl-gamma-semialdehyde dehydrogenase) (GSA dehydrogenase)	PPA0835	CYT
PFREUD_08540		Conserved protein, DUF143 domain	PPA0837	CYT
PFREUD_08600		Hypothetical protein	PPA0891	CYT
PFREUD_08610	leuS	Leucyl-tRNA synthetase (Leucine--tRNA ligase) (LeuRS)	PPA0893	CYT
PFREUD_08620	pyrC	Dihydroorotase multifunctional complex type (dihydroorotase)	PPA0998	CYT
PFREUD_08630	PPA0888	NAD-dependent malic enzyme (NAD-ME) (Malate dehydrogenase)	PPA0888	CYT
PFREUD_08660	holA	DNA polymerase III, delta subunit	PPA0897	CYT
PFREUD_08680	rpsT	30S Ribosomal protein S20p	PPA0898	CYT
PFREUD_08710	lepA	GTP-binding protein LepA	PPA0901	CYT
PFREUD_08720	hemM	Coproporphyrinogen oxidase / Oxygen-independent coproporphyrinogen III oxidase	PPA0911	CYT
PFREUD_08730		Hypothetical protein	PPA0912	CYT
PFREUD_08740		inositol-phosphate phosphatase	PPA0913	CYT
PFREUD_08750	hrcA	Heat-inducible transcription repressor hrcA	PPA0915	CYT
PFREUD_08760	dnaJ3	Chaperone protein dnaJ 3 (DnaJ3 protein) (Heat shock protein 40 3)	PPA0916	CYT
PFREUD_08770		Hypothetical protein	PPA0917	CYT
PFREUD_08790		Queuine tRNA-ribosyltransferase	PPA0924	CYT
PFREUD_08810	pip2	Proline iminopeptidase	PPA0932	CYT
PFREUD_08840	phoH	Phosphate starvation-inducible protein PhoH-like protein	PPA0936	CYT
PFREUD_08850		Hypothetical protein	PPA0937	CYT
PFREUD_08870	era	GTP-binding protein	PPA0939	CYT
PFREUD_08910		Metal-dependent hydrolase	PPA0940	CYT
PFREUD_08920	leuA1	2-isopropylmalate synthase	PPA0941	CYT
PFREUD_08930		Hypothetical protein	PPA0409	CYT
PFREUD_08940	uppS2	Undecaprenyl diphosphate synthase (di-trans,poly- cis-decaprenylcistransferase)	PPA0942	CYT
PFREUD_08950	recO	DNA repair protein recO (Recombination protein O)	PPA0944	CYT
PFREUD_09130	pduP	CoA-dependent propionaldehyde dehydrogenase PduP	PPA1741	CYT
PFREUD_09190	glyQS	Glycyl-tRNA synthetase (Glycine--tRNA ligase) (GlyRS)	PPA0949	CYT
PFREUD_09250	sdhA	Succinate dehydrogenase, subunit A	PPA0951	CYT
PFREUD_09260	sdhB	Succinate dehydrogenase, subunit B	PPA0952	CYT
PFREUD_09280	dgt	Deoxyguanosinetriphosphate triphosphohydrolase (dGTPase)	PPA0955	CYT
PFREUD_09320	cof	Cof-like hydrolase	PPA0973	CYT
PFREUD_09340	moxR	ATPases MoxR family	PPA0974	CYT
PFREUD_09350		Hypothetical protein	PPA0975	CYT
PFREUD_09380		alanine racemase	PPA0978	CYT
PFREUD_09400		Hypothetical protein	PPA0979	CYT
PFREUD_09410	fabF (fabB)	3-oxoacyl-[acyl-carrier-protein] synthase (Beta- ketoacyl-ACP synthase)	PPA0980	CYT
PFREUD_09420	acpP	Acyl carrier protein (ACP)	PPA0982	CYT
PFREUD_09440	pf3004	Carboxylic ester hydrolase	PPA0984	CYT
PFREUD_09450		Hypothetical protein	PPA0988	CYT
PFREUD_09470	aceE	dehydrogenase E1 component (2-oxo-acid dehydrogenase E1 subunit, homodimeric type)	PPA0989	CYT
PFREUD_09480		Hypothetical protein	PPA0991	CYT
PFREUD_09550	fnk	fructosamine kinase	PPA1005	CYT
PFREUD_09580	prfB	Peptide chain release factor 2 (RF-2)	PPA1354	CYT
PFREUD_09590	ftsE	ABC transporter, ATP-binding protein	PPA1353	CYT
PFREUD_09630	smpB	SmpB SsrA-binding protein	PPA1351	CYT
PFREUD_09650		Hypothetical protein	PPA1339	CYT
PFREUD_09660		two-component system response regulator	PPA1338	CYT
PFREUD_09670	prt	phosphoribosyltransferase	PPA1335	CYT
PFREUD_09680		ribosomal S30AE, sigma 54 modulation protein	PPA1334	CYT
PFREUD_09690		Preprotein translocase SecA subunit	PPA1333	CYT
PFREUD_09710		Hypothetical protein	PPA1331	CYT
PFREUD_09730		Hypothetical protein	PPA1330	CYT
PFREUD_09740	PPA1328	Predicted metal-dependent phosphoesterase	PPA1328	CYT
PFREUD_09750	trpS	Tryptophanyl-tRNA synthetase	PPA1327	CYT
PFREUD_09760		DEAD/DEAH box helicase domain protein	PPA1325	CYT
PFREUD_09780		Hypothetical protein	PPA1323	CYT
PFREUD_09810		ATP-dependent DNA helicase	PPA1320	CYT
PFREUD_09820	urvD	UvrD/REP helicase / ATP-dependent DNA helicase	PPA1319	CYT
PFREUD_09840	recD	Probable exodeoxyribonuclease V alpha chain RecD	PPA1317	CYT
PFREUD_09850	recB	Exodeoxyribonuclease V beta chain RecB	PPA1316	CYT
PFREUD_09860	recC	Exodeoxyribonuclease V gamma subunit RecC	PPA1315	CYT
PFREUD_09880	uvrD	DNA helicase	PPA1314	CYT
PFREUD_09910		metal dependant hydrolase	PPA1312	CYT
PFREUD_09920		Hypothetical protein	PPA1311	CYT
PFREUD_09950		Hypothetical protein	PPA1309	CYT
PFREUD_09970		Zn dependant peptidase	PPA0122	CYT
PFREUD_09990	pip1	Proline iminopeptidase	PPA2174	CYT
PFREUD_10000		acetyltransferase	PPA0096	CYT
PFREUD_10010	bioB	Biotin synthase	PPA1407	CYT
PFREUD_10060		ATP-binding ABC transporter protein	PPA1274	CYT
PFREUD_10070		ABC transporter ATP-binding protein	PPA1273	CYT
PFREUD_10080		D-3-phosphoglycerate dehydrogenase / erythronate 4-phosphate dehydrogenase	PPA1272	CYT
PFREUD_10160		monophosphatase	PPA1271	CYT
PFREUD_10170	engC	GTPase	PPA1269	CYT
PFREUD_10180	aroA	3-phosphoshikimate 1-carboxyvinyltransferase (phosphoenolpyruvate:3-phosphoshikimate5-O-(1- carboxyvinyl)-transferase) (EPSP synthase)	PPA1268	CYT
PFREUD_10200	rpoE	RNA polymerase sigma -70 factor, sigma-E factor	PPA1266	CYT
PFREUD_10210		anti-sigma factor	PPA2382	CYT
PFREUD_10230		Sensory transduction histidine kinase	PPA1265	CYT
PFREUD_10250	odhA	2-oxoglutarate dehydrogenase, E1 and E2 components	PPA1261	CYT
PFREUD_10300	lysA	Diaminopimelate decarboxylase (DAP decarboxylase)	PPA1259	CYT
PFREUD_10320	thrA/hom	Homoserine dehydrogenase	PPA1258	CYT
PFREUD_10340		Transcription termination factor Rho	PPA1254	CYT
PFREUD_10350	rpmE	50S ribosomal protein L31	PPA1253	CYT
PFREUD_10360	prfA	Peptide chain release factor 1 (RF-1)	PPA1252	CYT
PFREUD_10370	hemK	Methylase	PPA1250	CYT
PFREUD_10460	atpH	ATP synthase delta chain	PPA1242	CYT
PFREUD_10470	atpA	ATP synthase subunit alpha (ATPase subunit alpha) (ATP synthase F1 sector subunit alpha)	PPA1241	CYT
PFREUD_10480	atpG	ATP synthase gamma chain (ATP synthase F1 sector gamma subunit)	PPA1240	CYT
PFREUD_10490	atpD	ATP synthase subunit beta (ATPase subunit beta) (ATP synthase F1 sector subunit beta)	PPA1239	CYT
PFREUD_10530		Cobalamin adenosyltransferase	PPA1236	CYT
PFREUD_10540	pf1509	Putative carboxylic ester hydrolase	PPA1101	CYT

PFREUD_10560		Nuclease of the RecB family	PPA1102	CYT
PFREUD_10590		Methylmalonyl-CoA epimerase	PPA1104	CYT
PFREUD_10600	trxA3	Thioredoxin	PPA1106	CYT
PFREUD_10610	pgm1	Phosphoglucomutase	PPA1105	CYT
PFREUD_10620		NUDIX hydrolase	PPA1110	CYT
PFREUD_10630	glgB	1,4-alpha-glucan branching enzyme (Glycogen branching enzyme) (BE) (1,4-alpha-D-glucan:1,4-alpha-D- glucan 6 glucosyl- transferase)	PPA1111	CYT
PFREUD_10640	PPA1112	Uncharacterized protein probably involved in trehalose biosynthesis	PPA1112	CYT
PFREUD_10650	treS	Trehalose synthase	PPA1113	CYT
PFREUD_10660	glgE	Glycosyl hydrolase, family 13 (putative alpha- amylase, catalytic domain)	PPA1114	CYT
PFREUD_10700	glgX/treX	Glycogen debranching enzyme GlgX Isoamylase	PPA1115	CYT
PFREUD_10710	trmU	tRNA (5-methylaminomethyl-2-thiouridylate)- methyltransferase	PPA1117	CYT
PFREUD_10720	metE	Methionine synthase, vitamin-B12 independent	PPA1118	CYT
PFREUD_10740		Amino acid-binding ACT	PPA1121	CYT
PFREUD_10750	gatC	Glutamyl-tRNA(Gln) amidotransferase subunit C (Aspartyl/glutamyl-tRNA(Asn/Gln) amidotransferase subunit C)	PPA1122	CYT
PFREUD_10760	gatA	Glutamyl-tRNA(Gln) amidotransferase subunit A (Glu-ADT subunit A)	PPA1123	CYT
PFREUD_10770	gatB	Aspartyl/glutamyl-tRNA(Asn/Gln) amidotransferase subunit B (Asp/Glu-ADT subunit B)	PPA1124	CYT
PFREUD_10890	lpd	Dihydrolipoyl dehydrogenase (E3 component of alpha keto acid dehydrogenase complexes) (Dihydrolipoamide dehydrogenase)	PPA1227	CYT
PFREUD_10900	MurA	UDP-N-acetylglucosamine 1- carboxyvinyltransferase (Enoylpyruvate transferase)	PPA1226	CYT
PFREUD_10910	pdxY	Pyridoxal kinase	PPA1225	CYT
PFREUD_10930	glpQ	glycerophosphoryl diester phosphodiesterase	PPA1224	CYT
PFREUD_10940		Hypothetical protein	PPA1223	CYT
PFREUD_10950	dpm	dolichyl-phosphate beta-D-mannosyltransferase	PPA1222	CYT
PFREUD_10980		AsnC-family transcriptional regulatory protein	PPA1220	CYT
PFREUD_11050	luxS	S-ribosylhomocysteine lyase (Autoinducer-2 production protein luxS) (AI-2 synthesis protein)	PPA0450	CYT
PFREUD_11250		Hypothetical protein	PPA1342	CYT
PFREUD_11260	engA	GTP binding protein	PPA1210	CYT
PFREUD_11270	cmk	Cytidylate kinase (CK) (Cytidine monophosphate kinase) (CMP kinase)	PPA1209	CYT
PFREUD_11280	tyrA	Prephenate dehydrogenase	PPA1208	CYT
PFREUD_11290	prcA	20S proteasome alpha-subunit	PPA1207	CYT
PFREUD_11300	pcrB	20S proteasome beta-subunit	PPA1206	CYT
PFREUD_11350		Hypothetical protein	PPA1200	CYT
PFREUD_11370	rpe	Ribulose-phosphate 3-epimerase	PPA1199	CYT
PFREUD_11380		Sun, tRNA and rRNA cytosine-C5-methylases	PPA1196	CYT
PFREUD_11390	fmt	Methionyl-tRNA formyltransferase	PPA1195	CYT
PFREUD_11400	priA	Primosomal protein N	PPA1194	CYT
PFREUD_11410	metK	S-adenosylmethionine synthetase (Methionine adenosyltransferase) (AdoMet synthetase) (MAT)	PPA1193	CYT
PFREUD_11420	rpoZ	DNA-directed RNA polymerase omega chain (RNAP omega subunit) (Transcriptase omega chain) (RNA polymerase omega subunit)	PPA1191	CYT
PFREUD_11430	gmk	Guanylate kinase , Guanosine monophosphate kinase (GMP kinase)	PPA1190	CYT
PFREUD_11440	mihF	Integration host factor MihF	PPA1189	CYT
PFREUD_11470	carB (pyrA)	Carbamoyl-phosphate synthase large chain (Carbamoyl- phosphate synthetase ammonia chain)	PPA1000	CYT
PFREUD_11480	carA	Carbamoyl-phosphate synthase small chain	PPA0999	CYT
PFREUD_11530	aroF	Chorismate synthase (5-enolpyruvylshikimate-3- phosphate phospholyase)	PPA1182	CYT
PFREUD_11560	alaS	Alanyl-tRNA synthetase (Alanine--tRNA ligase) (AlaRS)	PPA1178	CYT
PFREUD_11570	ldh1	L-Lactate dehydrogenase	PPA1952	CYT
PFREUD_11580		Uncharacterized ATPase related to the helicase subunit of the holliday junction resolvase	PPA1176	CYT
PFREUD_11590	aspS	Aspartyl-tRNA synthetase (Aspartate--tRNA ligase) (AspRS)	PPA1175	CYT
PFREUD_11600		Hypothetical protein	PPA1174	CYT
PFREUD_11620	hisS	Histidyl-tRNA synthetase (Histidine--tRNA ligase) (HisRS)	PPA1169	CYT
PFREUD_11630		Beta-lactamase-like	PPA1168	CYT
PFREUD_11640		Hypothetical protein	PPA1167	CYT
PFREUD_11650	relA	GTP pyrophosphokinase	PPA1166	CYT
PFREUD_11660	apt	Adenine phosphoribosyltransferase (AMP:diphosphate phospho-D-ribosyltransferase)	PPA1164	CYT
PFREUD_11700	ruvB	Holliday junction ATP-dependent DNA helicase	PPA1160	CYT
PFREUD_11710	ruvA	Holliday junction ATP-dependent DNA helicase	PPA1159	CYT
PFREUD_11720	ruvC	Crossover junction endodeoxyribonuclease (Holliday junction nuclease) (Holliday junction resolvase)	PPA1158	CYT
PFREUD_11730		Hypothetical protein	PPA1157	CYT
PFREUD_11750	hisA	Phosphoribosyl isomerase A (1-(5-phosphoribosyl)- 5-[(5- phosphoribosylamino)methylideneamino] imidazole-4-carboxamide isomerase) (Phosphoribosylformimino-5- aminoimidazole carboxamide ribotide isomerase) (N-(5 -phosphoribosyl)anthranilate isomerase) (PRAI)	PPA1156	CYT
PFREUD_11760	hisH	Imidazole glycerol phosphate synthase subunit hisH (IGP synthase glutamine amidotransferase subunit) (IGP synthase subunit hisH) (ImGP synthase subunit hisH) (IGPS subunit hisH)	PPA1155	CYT
PFREUD_11770	hisB	Imidazoleglycerol-phosphate dehydratase (IGPD)	PPA1154	CYT
PFREUD_11780	hisC	Histidinol-phosphate aminotransferase (Imidazole acetol- phosphate transaminase)	PPA1153	CYT
PFREUD_11790	hisD	Histidinol dehydrogenase (HDH)	PPA1152	CYT
PFREUD_11810	ybaK/EbsC	YbaC/EbsC protein	PPA2262	CYT
PFREUD_11830	dnaE1	DNA polymerase III alpha subunit	PPA1150	CYT
PFREUD_11850	gltD	Glutamate synthase small subunit	PPA1135	CYT
PFREUD_11860	gltB	Glutamate synthase large subunit (Ferredoxin)	PPA1134	CYT
PFREUD_11890	trpB	Tryptophan synthase beta chain (TrpB)	PPA1131	CYT
PFREUD_11900	trpC	Indole-3-glycerol phosphate synthase (TrpC)	PPA1130	CYT
PFREUD_11920	hisI2	Phosphoribosyl-AMP cyclohydrolase	PPA1126	CYT
PFREUD_11930		Hypothetical protein	PPA1125	CYT
PFREUD_11940	glnS	Glutaminyl-tRNA synthetase	PPA1870	CYT
PFREUD_11990		Hypothetical protein	PPA1099	CYT
PFREUD_12020		Sua5_yciO_yrdC	PPA1092	CYT
PFREUD_12040	pfp (pfk)	pyrophosphate phosphofructokinase	PPA1090	CYT
PFREUD_12060		Hypothetical protein	PPA0959	CYT
PFREUD_12070	hrdD1	RNA polymerase principal sigma factor HrdD	PPA0958	CYT
PFREUD_12080	dnaG1	DNA primase	PPA0957	CYT
PFREUD_12170	otsA	Trehalose-6-phosphate synthase (Alpha,alpha- trehalose-phosphate synthase)	PPA0961	CYT
PFREUD_12190	cbiB/cobD	CbiB/CobD	PPA0418	CYT
PFREUD_12200	cbiP/cobQ	Cobyric acid synthase CbiP/CobQ	PPA0419	CYT
PFREUD_12210	cobA2	Cob(I)alamin adenosyltransferase	PPA0437	CYT
PFREUD_12300	gcvP	Glycine dehydrogenase [decarboxylating] (Glycine decarboxylase) (Glycine cleavage system P-protein)	PPA0742	CYT
PFREUD_12320			PPA1086	CYT
PFREUD_12330	merR1	Transcriptional regulator, MerR	PPA1085	CYT
PFREUD_12340		Forkhead-associated protein	PPA1084	CYT
PFREUD_12350	gcvH	Glycine cleavage H-protein (lipoate-binding)	PPA0743	CYT
PFREUD_12440	thrS	Threonyl-tRNA synthetase (Threonine--tRNA ligase) (ThrRS)	PPA1076	CYT

PFREUD_12500		Hypothetical protein	PPA1066	CYT
PFREUD_12520	msrB	L-methionine (S)-S-oxide reductase	PPA1065	CYT
PFREUD_12530	ybaK	YbaK / prolyl-tRNA synthetases	PPA2186	CYT
PFREUD_12540		Hypothetical protein	PPA1064	CYT
PFREUD_12550	rnd	Ribonuclease D (3 -5 exonuclease)	PPA1063	CYT
PFREUD_12560	dxs	Deoxyxylulose-5-phosphate synthase	PPA1062	CYT
PFREUD_12590	acn	Aconitase, Aconitate hydratase	PPA1061	CYT
PFREUD_12680		SAM-dependent methyltransferase related to tRNA (tRNA (uracil-5-)-methyltransferase)	PPA1060	CYT
PFREUD_12700	TrkA	Trk system potassium uptake protein TrkA	PPA1058	CYT
PFREUD_12710	trkA (ceoC)	Trk system potassium uptake protein TrkA (K(+)- uptake protein TrkA)	PPA1057	CYT
PFREUD_12720		Nucleic acid binding, OB-fold, tRNA/helicase- type	PPA1056	CYT
PFREUD_12770		Type I phosphodiesterase / nucleotide pyrophosphatase	PPA1048	CYT
PFREUD_12780		Hypothetical protein	PPA1047	CYT
PFREUD_12790		Hypothetical protein	PPA1046	CYT
PFREUD_12800	gyrA2	DNA gyrase subunit A (DNA topoisomerase (ATP- hydrolysing))	PPA1045	CYT
PFREUD_12810	panE	2-dehydropantoate 2-reductase	PPA0985	CYT
PFREUD_12820	gyrB1	DNA gyrase subunit B	PPA1042	CYT
PFREUD_12840	ldh2	L-lactate dehydrogenase	PPA0887	CYT
PFREUD_12850	hrdB	RNA polymerase principal sigma factor HrdB	PPA1032	CYT
PFREUD_12860	hrdD2	RNA polymerase principal sigma factor HrdD	PPA1031	CYT
PFREUD_12910	pf1637	Putative carboxylic ester hydrolase	PPA1030	CYT
PFREUD_12920	hrpA2	ATP-dependent helicase HrpA	PPA1029	CYT
PFREUD_12960	deoR2	DeoR transcriptional regulator	PPA2306	CYT
PFREUD_12970	glpC	Anaerobic glycerol-3-phosphate dehydrogenase subunit C	PPA2248	CYT
PFREUD_12980	glpB	Anaerobic glycerol-3-phosphate dehydrogenase subunit B	PPA2249	CYT
PFREUD_12990	glpA	Anaerobic glycerol-3-phosphate dehydrogenase subunit A	PPA2250	CYT
PFREUD_13010	nrdJ	Vitamin B12-dependent ribonucleotide reductase (Ribonucleoside-diphosphate reductase NrdJ)	PPA1026	CYT
PFREUD_13020	nrdR	Transcriptional repressor nrdR	PPA1025	CYT
PFREUD_13050	dinG	ATP-dependent helicase	PPA1022	CYT
PFREUD_13060		FeoB, Ferrous iron transport protein B	PPA1021	CYT
PFREUD_13070	dapF	Diaminopimelate epimerase	PPA1020	CYT
PFREUD_13080	miaA	TRNA isopentenyltransferase	PPA1019	CYT
PFREUD_13090	miaB1	2-methylthioadenine synthetase MiaB protein	PPA1017	CYT
PFREUD_13130	recA	RecA (Recombinase A)	PPA1012	CYT
PFREUD_13140		Hypothetical protein	PPA1011	CYT
PFREUD_13180	cinA	CinA, Competence-damaged protein	PPA1008	CYT
PFREUD_13200	miaB2	2-methylthioadenine synthetase MiaB protein	PPA1006	CYT
PFREUD_13220	thiL	Thiamine monophosphate kinase	PPA1357	CYT
PFREUD_13250	ddlA	D-alanine--D-alanine ligase (D-alanylalanine synthetase)	PPA1359	CYT
PFREUD_13260	plsC1	1-acylglycerol-3-phosphate O-acyltransferase	PPA1360	CYT
PFREUD_13290	iclR	IclR transcriptional regulator	PPA1364	CYT
PFREUD_13330	PPA1366	Putative 2-hydroxyhepta-2,4-diene-1,7-dioate isomerase	PPA1366	CYT
PFREUD_13350	ilvE (bcaT)	Branched-chain amino acid aminotransferase	PPA1368	CYT
PFREUD_13390	ilvC	Ketol-acid reductoisomerase (Acetohydroxy-acid isomeroreductase) (Alpha-keto-beta-hydroxylacil reductoisomerase)	PPA1372	CYT
PFREUD_13430	hisF	Imidazole glycerol phosphate synthase subunit HisF	PPA1376	CYT
PFREUD_13460		Superfamily II RNA helicase	PPA1377	CYT
PFREUD_13470	cof2	Cof protein:HAD-superfamily hydrolase subfamily IIB	PPA0211	CYT
PFREUD_13530		Regulatory protein	PPA1381	CYT
PFREUD_13540		DeoR-family transcriptional regulator	PPA1382	CYT
PFREUD_13550	gluQ	Glutamyl-Q tRNA(Asp) synthetase (Glu-Q-RSs), Glutamyl-Q tRNA(Asp) synthetase	PPA1383	CYT
PFREUD_13580	rluB	Pseudouridine synthase	PPA1385	CYT
PFREUD_13620		ATPase involved in cell division	PPA1387	CYT
PFREUD_13630	xerD	Site-specific recombinase	PPA1388	CYT
PFREUD_13640	nudF	ADP-ribose pyrophosphatase	PPA1389	CYT
PFREUD_13650	pyrG	CTP synthase (UTP--ammonia ligase) (CTP synthetase)	PPA1390	CYT
PFREUD_13660	recN	DNA repair protein	PPA1394	CYT
PFREUD_13670	ppnK	Probable inorganic polyphosphate/ATP-NAD kinase (Poly(P)/ATP NAD kinase)	PPA1395	CYT
PFREUD_13730	nagD	Phosphatase	PPA1401	CYT
PFREUD_13930	tyrS	Tyrosyl-tRNA synthetase (Tyrosine--tRNA ligase) (TyrRS)	PPA1404	CYT
PFREUD_13940	argH	Argininosuccinate lyase (Arginosuccinase)	PPA1346	CYT
PFREUD_13950	argR	Arginine repressor, ArgR	PPA0586	CYT
PFREUD_13960	argD	Acetylornithine and succinylornithine aminotransferase (ACOAT)	PPA1347	CYT
PFREUD_13970	argB	Acetylglutamate kinase	PPA1348	CYT
PFREUD_13990	argC	N-acetyl-gamma-glutamyl-phosphate reductase (AGPR) (N- acetyl-glutamate semialdehyde dehydrogenase) (NAGSA dehydrogenase)	PPA1350	CYT
PFREUD_14010	pheT	Phenylalanyl-tRNA synthetase beta chain (Phenylalanine-- tRNA ligase beta chain) (PheRS)	PPA1408	CYT
PFREUD_14020	pheS	Phenylalanyl-tRNA synthetase alpha chain (Phenylalanine--tRNA ligase alpha chain) (PheRS)	PPA1409	CYT
PFREUD_14040	spoU1	rRNA methylase	PPA1410	CYT
PFREUD_14050	rplT	50S ribosomal protein L20	PPA1412	CYT
PFREUD_14060	rpmI	50S ribosomal protein L35	PPA1413	CYT
PFREUD_14070	infC	Translation initiation factor IF-3	PPA1414	CYT
PFREUD_14100	hisG	ATP phosphoribosyltransferase	PPA1417	CYT
PFREUD_14110	hisE	Phosphoribosyl-ATP pyrophosphohydrolase	PPA1418	CYT
PFREUD_14140		oxidoreductase	PPA1422	CYT
PFREUD_14230	dprA	DNA processing / uptake protein	PPA1429	CYT
PFREUD_14240	chlI	Magnesium chelatase, subunit ChlI	PPA1430	CYT
PFREUD_14260		Hypothetical protein	PPA1432	CYT
PFREUD_14270	rnhB	Ribonuclease HII	PPA1433	CYT
PFREUD_14290	rplS	50S ribosomal protein L19	PPA1435	CYT
PFREUD_14300	sdhB3	Succinate dehydrogenase	PPA1437	CYT
PFREUD_14340	trmD	tRNA (guanine-N1-)-methyltransferase / S- adenosyl-L-methionine:tRNA (guanine-N1-)- methyltransferase	PPA1440	CYT
PFREUD_14350	rimM	16S rRNA processing protein	PPA1441	CYT
PFREUD_14360		Hypothetical protein	PPA1442	CYT
PFREUD_14380		Metallo-dependent hydrolases, subgroup A	PPA1444	CYT
PFREUD_14390	ffh	GTP binding signal recognition particle protein	PPA1445	CYT
PFREUD_14440	mutM3	Formamidopyrimidine-DNA glycosylase (Fapy-DNA glycosylase) (DNA-(apurinic or apyrimidinic site) lyase mutM) (AP lyase mutM)	PPA1451	CYT
PFREUD_14470		Hypothetical protein	PPA1453	CYT
PFREUD_14480	coaD	Phosphopantetheine adenylyltransferase (Pantetheine- phosphate adenylyltransferase) (PPAT) (Dephospho-CoA pyrophosphorylase)	PPA1463	CYT
PFREUD_14490		Methylase	PPA1464	CYT
PFREUD_14500		Hypothetical protein	PPA1465	CYT

PFREUD_14510	recG	ATP-dependent DNA helicase RecG	PPA1466	CYT
PFREUD_14530		Beta-lactamase-like:RNA-metabolising metallo- beta-lactamase	PPA1467	CYT
PFREUD_14560	dapB	Dihydrodipicolinate reductase (DHPR)	PPA1470	CYT
PFREUD_14570	pnpA	Polyribonucleotide nucleotidyltransferase (Polynucleotide phosphorylase) (PNPase) (Guanosine pentaphosphate synthetase)	PPA1471	CYT
PFREUD_14580	rpsO	30S ribosomal protein S15	PPA1472	CYT
PFREUD_14600	glpK	Glycerol kinase (ATP:glycerol 3- phosphotransferase) (Glycerokinase) (GK)	PPA2304	CYT
PFREUD_14620	truB	tRNA pseudouridine synthase B (tRNA pseudouridine 55 synthase)	PPA1478	CYT
PFREUD_14630	rbfA	Ribosome-binding factor A	PPA1489	CYT
PFREUD_14640	infB	Translation initiation factor IF-2	PPA1493	CYT
PFREUD_14660	nusA	Transcription termination/ antitermination factor NusA	PPA1494	CYT
PFREUD_14670		Hypothetical protein	PPA1495	CYT
PFREUD_14690	proS	Prolyl-tRNA synthetase (Proline--tRNA ligase) (ProRS)	PPA1499	CYT
PFREUD_14700		Acetyltransferase family protein	PPA1505	CYT
PFREUD_14710	ispG	4-hydroxy-3-methylbut-2-en-1-yl diphosphate synthase (1- hydroxy-2-methyl-2-(E)-butenyl 4-diphosphate synthase)	PPA1506	CYT
PFREUD_14720	dxr	1-deoxy-D-xylulose 5-phosphate reductoisomerase (DXP reductoisomerase) (1-deoxyxylulose-5-phosphate reductoisomerase) (2-C- methyl-D-erythritol 4-phosphate synthase)	PPA1510	CYT
PFREUD_14730		Hypothetical protein	PPA1514	CYT
PFREUD_14750	frr	Ribosome recycling factor (Ribosome-releasing factor) (RRF)	PPA1518	CYT
PFREUD_14760	pyrH	Uridylate kinase PyrH	PPA1519	CYT
PFREUD_14770	tsf	Elongation factor Ts (EF-Ts)	PPA1520	CYT
PFREUD_14780	rpsB	30S ribosomal protein S2	PPA1521	CYT
PFREUD_14800	xerC	Integrase/recombinase	PPA1522	CYT
PFREUD_14810	def	Polypeptide deformylase	PPA1525	CYT
PFREUD_14820	spoU2	RRNA methylase family protein (TRNA/rRNA methyltransferase)	PPA1526	CYT
PFREUD_14840	PPA1530	ABC transporter ATP-binding protein	PPA1530	CYT
PFREUD_14850		Hypothetical protein	PPA1531	CYT
PFREUD_14860	inhA	Enoyl-[acyl-carrier-protein] reductase (NADH)	PPA1532	CYT
PFREUD_14870	fabG	3-oxoacyl-[acyl-carrier protein] reductase	PPA1533	CYT
PFREUD_14890	myrA	resistance protein	PPA1536	CYT
PFREUD_14910		ABC transporter ATP binding protein	PPA1538	CYT
PFREUD_14930	nifU1	Nitrogen-fixing NifU-like	PPA1544	CYT
PFREUD_14940	sufS	Cysteine desulphurases, SufS	PPA1545	CYT
PFREUD_14950	sufC	ABC-type transport system involved in Fe-S cluster assembly, ATPase component, SufC	PPA1546	CYT
PFREUD_14960		Dioxygenase	PPA1547	CYT
PFREUD_14970	sufD	FeS assembly protein SufD	PPA1548	CYT
PFREUD_14980	sufB	FeS assembly protein SufB	PPA1549	CYT
PFREUD_14990	arsR4	Transcriptional regulator, ArsR family	PPA1550	CYT
PFREUD_15030	pdxT	Glutamine amidotransferase subunit pdxT (Glutamine amidotransferase glutaminase subunit pdxT)	PPA0966	CYT
PFREUD_15040	pdxS	Pyridoxal biosynthesis lyase pdxS	PPA0965	CYT
PFREUD_15050		Transcriptional regulator	PPA0964	CYT
PFREUD_15060	zwf	Glucose-6-phosphate 1-dehydrogenase	PPA1563	CYT
PFREUD_15070	opcA	glucose 6-phosphate dehydrogenase effector OpcA	PPA1564	CYT
PFREUD_15080	devB	6-phosphogluconolactonase	PPA1565	CYT
PFREUD_15090		electron transport protein	PPA0820	CYT
PFREUD_15110	tpi1	triosephosphate isomerase 1	PPA0818	CYT
PFREUD_15120	pgk	Phosphoglycerate kinase	PPA0817	CYT
PFREUD_15130	gap	Glyceraldehyde-3-phosphate dehydrogenase / erythrose 4 phosphate dehydrogenase	PPA0816	CYT
PFREUD_15170		Hypothetical protein	PPA0815	CYT
PFREUD_15180		Hypothetical protein	PPA0814	CYT
PFREUD_15190		ATPase	PPA0813	CYT
PFREUD_15200	uvrC	UvrABC system protein C (Protein uvrC) (Excinuclease ABC subunit C)	PPA0812	CYT
PFREUD_15220		two-component response regulator	PPA0926	CYT
PFREUD_15230		ABC transporter, ATP-binding protein	PPA0927	CYT
PFREUD_15260	uvrA3	UvrABC system protein A (UvrA protein) (Excinuclease ABC subunit A)	PPA0807	CYT
PFREUD_15270		Hypothetical protein	PPA0806	CYT
PFREUD_15280		Beta-lactamase-like	PPA0805	CYT
PFREUD_15300	uvrB	UvrABC system protein B (Protein uvrB) (Excinuclease ABC subunit B)	PPA0803	CYT
PFREUD_15310	coaE	Dephospho-CoA kinase (Dephosphocoenzyme A kinase)	PPA0785	CYT
PFREUD_15330	rpsA	30S ribosomal protein S1	PPA0778	CYT
PFREUD_15340	gab	Aldehyde dehydrogenase (Succinate-semialdehyde dehydrogenase) (NAD-dependent aldehyde dehydrogenase)	PPA0079	CYT
PFREUD_15360	polA	Putative DNA polymerase I	PPA0774	CYT
PFREUD_15370		two-component system response regulator	PPA0772	CYT
PFREUD_15390	pyk1	Pyruvate kinase 1	PPA0769	CYT
PFREUD_15400	rluA	Pseudouridylate synthase, Uncharacterized RNA pseudouridine synthase	PPA0768	CYT
PFREUD_15420	dskA	Transcriptional regulators, TraR/DksA family	PPA0766	CYT
PFREUD_15430		Hypothetical protein	PPA0765	CYT
PFREUD_15450		Hypothetical protein	PPA0763	CYT
PFREUD_15460		Hypothetical protein	PPA0762	CYT
PFREUD_15470	ftsZ	Cell division protein FtsZ	PPA0761	CYT
PFREUD_15490	murC	UDP-N-acetylmuramate--L-alanine ligase (UDP-N- acetylmuramoyl-L-alanine synthetase)	PPA0759	CYT
PFREUD_15500	murG	UDP-N-acetylglucosamine--N-acetylmuramyl- (pentapeptide) pyrophosphoryl-undecaprenol N- acetylglucosamine transferase (Undecaprenyl-PP-MurNAc- pentapeptide-UDPGlcNAc GlcNAc transferase)	PPA0758	CYT
PFREUD_15520	murD	UDP-N-acetylmuramoylalanine--D-glutamate ligase (UDP-N- acetylmuramoyl-L-alanyl-D-glutamate synthetase) (D-glutamic acid- adding enzyme)	PPA0756	CYT
PFREUD_15540	murF	UDP-N-acetylmuramoyl-tripeptide--D-alanyl-D- alanine ligase (UDP- MurNAc-pentapeptide synthetase) (D- alanyl-D-alanine-adding enzyme)	PPA0754	CYT
PFREUD_15550	murE	UDP-N-acetylmuramoylalanyl-D-glutamate--2,6- diaminopimelate ligase (UDP-N-acetylmuramyl-tripeptide synthetase) (Meso- diaminopimelate-adding enzyme) (UDP- MurNAc-tripeptide synthetase)	PPA0753	CYT
PFREUD_15560	ftsI	Cell division protein FtsI (penicillin-binding protein 2) (Peptidoglycan glycosyltransferase)	PPA0752	CYT
PFREUD_15580	mraW	S-adenosyl-L-methionine-dependent methyltransferase mraW	PPA0750	CYT
PFREUD_15590	mraZ	Protein mraZ	PPA0749	CYT
PFREUD_15640	dnaE2	DNA polymerase III alpha subunit	PPA1650	CYT
PFREUD_15660		Helicase protein	PPA0733	CYT
PFREUD_15680		Farnesyltranstransferase / Geranylgeranyl pyrophosphate synthase	PPA0732	CYT
PFREUD_15710		Zn-dependent hydrolase, beta-lactamase fold	PPA0727	CYT
PFREUD_15720	aroH	Phospho-2-dehydro-3-deoxyheptonate aldolase	PPA0726	CYT
PFREUD_15740	plsC3	Acyltransferase PlsC	PPA0724	CYT
PFREUD_15770	ppiA (cypB)	Probable peptidyl-prolyl cis-trans isomerase A	PPA0718	CYT
PFREUD_15850	guaB3	Inosine-5 -monophosphate dehydrogenase (IMP dehydrogenase) (IMPDH) (IMPD) / GMP reductase	PPA0708	CYT

PFREUD_15860		HesB protein	PPA0700	CYT
PFREUD_15880		Hypothetical protein	PPA0699	CYT
PFREUD_15890	pspA	Phage shock protein A	PPA0698	CYT
PFREUD_15920		Hypothetical protein	PPA0696	CYT
PFREUD_15930	gcvT1	Glycine cleavage system T protein, aminomethyltransferase	PPA0744	CYT
PFREUD_15960		ABC transporter	PPA0682	CYT
PFREUD_15970		Glutamine synthetase	PPA0671	CYT
PFREUD_15990		Tetracycline repressor protein, TetR-family transcriptional regulator	PPA1406	CYT
PFREUD_16010	glnE	Glutamate-ammonia-ligase adenylyltransferase ([Glutamate--ammonia-ligase] adenylyltransferase) (Glutamine-synthetase adenylyltransferase) (ATase)	PPA0666	CYT
PFREUD_16020		Hypothetical protein	PPA0665	CYT
PFREUD_16030	glnA	Glutamine synthetase	PPA0664	CYT
PFREUD_16060	map, mapB	Methionine aminopeptidase (MAP) (Peptidase M)	PPA0661	CYT
PFREUD_16080		Hypothetical protein	PPA0651	CYT
PFREUD_16090	pepP	Xaa-Pro aminopeptidase I	PPA0650	CYT
PFREUD_16110	citE	Citrate lyase beta chain (Citrase beta chain) (Citrate(Pro-3S)-lyase beta chain)	PPA1919	CYT
PFREUD_16120	mgtE	magnesium (Mg2+) transporter	PPA0647	CYT
PFREUD_16140	PF47	Protein mrp homolog (ATP-binding protein)	PPA0645	CYT
PFREUD_16150	tatB	Sec-independent protein translocase protein TatB precursor	PPA0643	CYT
PFREUD_16160	dapA	Dihydrodipicolinate synthase	PPA0642	CYT
PFREUD_16170		Methyltransferase	PPA0641	CYT
PFREUD_16180	glgC	Glucose-1-phosphate adenylyltransferase (ADP- glucose synthase) (ADP-glucose pyrophosphorylase) (ADPGlc PPase)	PPA0640	CYT
PFREUD_16190	glgA	glycosyltransferase (glycogen synthase)	PPA0639	CYT
PFREUD_16200		Hypothetical protein	PPA0638	CYT
PFREUD_16240	dapE2	Succinyl-diaminopimelate desuccinylase	PPA0636	CYT
PFREUD_16250	dapD	2,3,4,5-tetrahydropyridine-2,6-dicarboxylate N- succinyltransferase	PPA0630	CYT
PFREUD_16270		aminotransferase	PPA0628	CYT
PFREUD_16280	fdxA	Ferredoxin	PPA0627	CYT
PFREUD_16290	gya	Glyoxylate reductase	PPA2251	CYT
PFREUD_16300	fumC	Fumarate hydratase, class-II	PPA2292	CYT
PFREUD_16320	aspA1	Aspartate ammonia-lyase (Aspartase)	PPA0094	CYT
PFREUD_16370		Acetyltransferase	PPA0624	CYT
PFREUD_16380	gpt	Putative purine phosphoribosyltransferase	PPA0623	CYT
PFREUD_16400		Hypothetical protein	PPA1744	CYT
PFREUD_16460		Hypothetical protein	PPA0587	CYT
PFREUD_16490	glxK/garK	Glycerate kinase GlxK/GarK	PPA2299	CYT
PFREUD_16550	thiG	Thiazole biosynthesis family protein	PPA0519	CYT
PFREUD_16570	dadA1	DadA, Glycine/D-amino acid oxidases	PPA0520	CYT
PFREUD_16930	ychF	GTPase YchF	PPA0575	CYT
PFREUD_16940	apeB	Aminopeptidase	PPA0574	CYT
PFREUD_16970	ispH (lytB)	4-hydroxy-3-methylbut-2-enyl diphosphate reductase	PPA0572	CYT
PFREUD_16980	xseA	Exodeoxyribonuclease VII large subunit	PPA0569	CYT
PFREUD_17010	uppS	Undecaprenyl pyrophosphate synthase (di- trans,poly-cis-decaprenylcistransferase)	PPA0564	CYT
PFREUD_17050	greA	Transcription elongation factor GreA	PPA0561	CYT
PFREUD_17060		Hypothetical membrane protein	PPA0920	CYT
PFREUD_17070		Hypothetical protein	PPA0560	CYT
PFREUD_17090	ung	Uracil-DNA glycosylase	PPA0558	CYT
PFREUD_17100	msrA	peptide-methionine (S)-S-oxide reductase	PPA0556	CYT
PFREUD_17180	mpg	3-methyladenine DNA glycosylase	PPA2247	CYT
PFREUD_17200		NUDIX hydrolase	PPA0550	CYT
PFREUD_17290	ppx	Ppx/GppA phosphatase family	PPA0548	CYT
PFREUD_17300		Hypothetical protein	PPA0547	CYT
PFREUD_17320	eno1	Enolase 1	PPA0545	CYT
PFREUD_17330		MazG-family transcriptional regulator	PPA0543	CYT
PFREUD_17350	mfd	Transcription-repair coupling factor	PPA0541	CYT
PFREUD_17370	pth	Peptidyl-tRNA hydrolase (aminoacyl-tRNA hydrolase)	PPA0536	CYT
PFREUD_17400	prs	Ribose-phosphate pyrophosphokinase	PPA0531	CYT
PFREUD_17440	pyrB	Aspartate carbamoyltransferase	PPA0997	CYT
PFREUD_17460	ispE	4-diphosphocytidyl-2-C-methyl-D-erythritol kinase (CMK) (4-(cytidine-5 -diphospho)-2-C-methyl-D- erythritol kinase)	PPA0527	CYT
PFREUD_17470	ksgA	Dimethyladenosine transferase (S- adenosylmethionine-6-N , N -adenosyl(rRNA) dimethyltransferase) (16S rRNA dimethylase) (High level kasugamycin resistance protein ksgA) (Kasugamycin dimethyltransferase)	PPA0526	CYT
PFREUD_17480	tatD	DNase	PPA0525	CYT
PFREUD_17490		Methylase	PPA0524	CYT
PFREUD_17610	gdh	glutamate dehydrogenase (NAD(P)-glutamate dehydrogenase)	PPA1513	CYT
PFREUD_17840	dnaK1	Chaperone protein dnaK 1 (Heat shock protein 70 1) (Heat shock 70 kDa protein 1) (HSP70 1)	PPA2040	CYT
PFREUD_17920	clpB 2	Chaperone clpB 2 (ATP-dependent Clp protease B2) (Clp chaperone)	PPA2021	CYT
PFREUD_17990	rimJ	ribosomal-protein-alanine acetyltransferase	PPA0495	CYT
PFREUD_18000	moeA1	Molybdenum cofactor synthesis domain	PPA0494	CYT
PFREUD_18070	ugpA	UTP--glucose-1-phosphate uridylyltransferase	PPA0489	CYT
PFREUD_18140	trxA4	thiredoxine	PPA0487	CYT
PFREUD_18150	dtxR	Iron-dependent repressor	PPA0485	CYT
PFREUD_18160	serC	Phosphoserine aminotransferase (phosphoserine transaminase)	PPA0483	CYT
PFREUD_18200		Hypothetical protein	PPA0478	CYT
PFREUD_18210	cspB	Cold shock protein	PPA0477	CYT
PFREUD_18230		Hypothetical protein	PPA0474	CYT
PFREUD_18470	groL2 (groEL2) (hsp60)	60 kDa chaperonin 2 (Protein Cpn60 2) (groEL protein 2) (Heat shock protein 60 2)	PPA0453	CYT
PFREUD_18490	manA	Phosphomannose isomerase	PPA0451	CYT
PFREUD_18570	sdaA	L-serine dehydratase (L-serine ammonia-lyase)	PPA0396	CYT
PFREUD_18580		ABC transporter ATP-binding protein	PPA0395	CYT
PFREUD_18600		Hypothetical protein	PPA0386	CYT
PFREUD_18630		Hypothetical protein	PPA0169	CYT
PFREUD_18640		iron-sulfur protein	PPA0168	CYT
PFREUD_18650		Oxidoreductase	PPA0167	CYT
PFREUD_18680	cysS2	Cysteinyl-tRNA synthetase (Cysteine--tRNA ligase)	PPA0384	CYT
PFREUD_18690		tRNA/rRNA methyltransferase	PPA0383	CYT
PFREUD_18730	ansA	L-asparaginase I	PPA0367	CYT
PFREUD_18740	glyA	Glycine hydroxymethyltransferase precursor	PPA0369	CYT
PFREUD_18750	ispF	2-C-methyl-D-erythritol 2,4-cyclodiphosphate synthase (MECPS) (MECDP-synthase)	PPA0354	CYT
PFREUD_18760	ispD	2-C-methyl-D-erythritol 4-phosphate cytidylyltransferase (4-diphosphocytidyl-2C-methyl-D- erythritol synthase) (MEP cytidylyltransferase) (MCT)	PPA0353	CYT

PFREUD_18770	carD	transcriptional regulator CarD	PPA0357	CYT
PFREUD_18780	regX	Response regulator receiver	PPA0359	CYT
PFREUD_18800	phoU	Putative phosphate transport system protein	PPA0361	CYT
PFREUD_18810	gpm2	phosphoglycerate mutase	PPA0364	CYT
PFREUD_18830	sdh	Serine 3-dehydrogenase	PPA0350	CYT
PFREUD_18840	bccp	Methylmalonyl-CoA carboxytransferase , 1.3S subunit (Transcarboxylase, 1.3S subunit). 123bp	PPA2005	CYT
PFREUD_18850		Hypothetical protein	PPA2006	CYT
PFREUD_18860	mmdA	Methylmalonyl-CoA carboxytransferase 12S subunit (EC2.1.3.1) (Transcarboxylase 12S subunit). 610 bp	PPA2007	CYT
PFREUD_18870		Methylmalonyl-CoA carboxytransferase 5S subunit. (transcarboxylase 5S) 505 bp	PPA2008	CYT
PFREUD_18880	hemL1	Glutamate-1-semialdehyde 2,1-aminomutase (GSA) (Glutamate-1-semialdehyde aminotransferase)(GSA-AT)	PPA0301	CYT
PFREUD_18910	hemB	Delta-aminolevulinic acid dehydratase (Porphobilinogen synthase)	PPA0302	CYT
PFREUD_18930	hemY	Protoporphyrinogen oxidase (PPO) HemY	PPA2095	CYT
PFREUD_18940		Fe-S oxidoreductase	PPA2096	CYT
PFREUD_18970	hemA	Glutamyl-tRNA reductase	PPA0307	CYT
PFREUD_19030	iolG1	iolG1 (Myo-inositol catabolism IolG1 protein) (myo-inositol 2-dehydrogenase)	PPA0469	CYT
PFREUD_19040	tal2	Transaldolase 2	PPA0454	CYT
PFREUD_19050		GntR-family protein transcriptional regulator	PPA0455	CYT
PFREUD_19060	iolC	iolC (Myo-inositol catabolism iolC protein)	PPA0456	CYT
PFREUD_19070		Hypothetical protein	PPA0457	CYT
PFREUD_19080	iolB	iolB (Myo-inositol catabolism IolB protein)	PPA0458	CYT
PFREUD_19090	iolD	iolD (Myo-inositol catabolism iolD protein) (acetolactate synthase protein) (pyruvate:pyruvate acetaldehydetransferase (decarboxylating))	PPA0459	CYT
PFREUD_19100	iolA (msdA)	iolA (Myo-inositol catabolism IolA protein) (Methylmalonic acid semialdehyde dehydrogenase)	PPA0461	CYT
PFREUD_19150	fba1	Fructose-bisphosphate aldolase class II	PPA2011	CYT
PFREUD_19230	speE	spermidine synthase	PPA2016	CYT
PFREUD_19280	purA	Adenylosuccinate synthetase (IMP--aspartate ligase) (AdSS) (AMPSase)	PPA1994	CYT
PFREUD_19310	iunH	Inosine/uridine-preferring nucleoside hydrolase (purine nucleosidase)	PPA2076	CYT
PFREUD_19430		Endoribonuclease L-PSP	PPA0221	CYT
PFREUD_19460	hslR	Ribosome-associated heat shock protein implicated in the recycling of the 50S subunit (S4 paralog)	PPA0202	CYT
PFREUD_19480		Phosphocarrier, HPr family	PPA0141	CYT
PFREUD_19560		Forkhead-associated protein	PPA0189	CYT
PFREUD_19630	galK2	Galactokinase	PPA2192	CYT
PFREUD_19640	nifU2	NifU-like protein	PPA1678	CYT
PFREUD_19710	mesJ	CMP/dCMP deaminase, zinc-binding	PPA2182	CYT
PFREUD_19730	upp	Uracil phosphoribosyltransferase (UMP pyrophosphorylase) (UPRTase)	PPA2184	CYT
PFREUD_19810	typA	GTP-binding protein typA/BipA (Tyrosine phosphorylated protein A)	PPA2003	CYT
PFREUD_19820	gcvT2	glycine cleavage T-protein, aminomethyl transferase	PPA0348	CYT
PFREUD_19830		Hypothetical protein	PPA0347	CYT
PFREUD_19860	dtd	D-tyrosyl-tRNA(Tyr) deacylase	PPA0346	CYT
PFREUD_19870	glnR	Transcriptional regulatory protein	PPA0344	CYT
PFREUD_19910	galE3	UDP-glucose 4-epimerase	PPA0069	CYT
PFREUD_19930	ppk	Polyphosphate kinase	PPA0343	CYT
PFREUD_19940		NUDIX hydrolase NTP pyrophosphohydrolases including oxidative damage repair enzymes	PPA0342	CYT
PFREUD_19980	pstB	Phosphate import ATP-binding protein pstB (Phosphate- transporting ATPase) (ABC phosphate transporter)	PPA0338	CYT
PFREUD_20050		Pyridine nucleotide-disulphide oxidoreductase	PPA0333	CYT
PFREUD_20060	pgm/fbp	phosphoglycerate mutase/Fructose-2,6- bisphosphatase	PPA0324	CYT
PFREUD_20070	hemD	Uroporphyrinogen-III synthase HemD	PPA0323	CYT
PFREUD_20090		Delta 1-pyrroline-5-carboxylate reductase	PPA0319	CYT
PFREUD_20130		endonuclease	PPA0315	CYT
PFREUD_20140		Hypothetical protein	PPA0314	CYT
PFREUD_20150	sms	DNA repair protein	PPA0312	CYT
PFREUD_20160	disA	DNA integrity scanning protein disA	PPA0311	CYT
PFREUD_20170	mutY	MutY, A/G-specific DNA glycosylase	PPA0300	CYT
PFREUD_20210		cobalt ABC transporter	PPA0288	CYT
PFREUD_20250	clpC	chaperone clpC (Clp-family ATP-binding protease) (ATP-dependent Clp protease ATP-binding subunit)	PPA0278	CYT
PFREUD_20260		Lsr2-like protein	PPA0277	CYT
PFREUD_20270	folK	Putative hydroxymethyldihydropteridine pyrophosphokinase	PPA0273	CYT
PFREUD_20280	folB	Dihydroneopterin aldolase	PPA0272	CYT
PFREUD_20290	folP2	Dihydropteroate synthase((2-amino-4-hydroxy-7,8- dihydropteridin-6- yl)methyl-diphosphate:4-aminobenzoate 2-amino-4-hydroxydihydropteridine-6-methenyltransferase)	PPA0271	CYT
PFREUD_20310	folE	GTP cyclohydrolase I (GTP-CH-I)	PPA0261	CYT
PFREUD_20330	hpt	hypoxanthine phosphoribosyltransferase	PPA0259	CYT
PFREUD_20340	tilS	TRNA(Ile)-lysidine synthetase	PPA0258	CYT
PFREUD_20380		Hypothetical protein	PPA0255	CYT
PFREUD_20390	sixA	phosphohistidine phosphatase	PPA0252	CYT
PFREUD_20440	mao	flavin-containing amine oxidase	PPA0250	CYT
PFREUD_20530	dnaX	DNA polymerase III, delta prime subunit	PPA0244	CYT
PFREUD_20540	tmk	Thymidylate kinase (dTMP kinase)	PPA0243	CYT
PFREUD_20550		Hypothetical protein	PPA0242	CYT
PFREUD_20560	topA	DNA topoisomerase I	PPA0241	CYT
PFREUD_20570		transferase	PPA0238	CYT
PFREUD_20600	dmsB	Anaerobic dimethyl sulfoxide reductase, chain B	PPA0516	CYT
PFREUD_20610	dmsA	Anaerobic dimethyl sulfoxide reductase chain A	PPA0517	CYT
PFREUD_20710		secretory protein	PPA0230	CYT
PFREUD_20720		Hypothetical protein	PPA0229	CYT
PFREUD_20770		Hypothetical protein	PPA2173	CYT
PFREUD_20800		Hypothetical protein	PPA2243	CYT
PFREUD_20810	dnaB	Replicative DNA helicase	PPA2244	CYT
PFREUD_20840	namA	NADH-dependent flavin oxidoreductase	PPA2118	CYT
PFREUD_21320	merA	Pyridine nucleotide-disulphide oxidoreductase	PPA1981	CYT
PFREUD_21390	modB	DNA methylase (site-specific DNA- methyltransferase adenine-specific, Putative type III restriction-modification system)	PPA1611	CYT
PFREUD_21400		Putative type III restriction enzyme	PPA1612	CYT
PFREUD_21440	ribH	6,7-dimethyl-8-ribityllumazine synthase (Riboflavin synthase beta chain)	PPA1749	CYT
PFREUD_21450	ribAB	GTP cyclohydrolase II protein, Riboflavin biosynthesis protein	PPA1750	CYT
PFREUD_21460	ribC (ribE)	Riboflavin synthase alpha chain	PPA1751	CYT
PFREUD_21470	ribD	Riboflavin-specific deaminase (diaminohydroxyphosphoribosylaminopyrimidine deaminase) (5- amino-6-(5 phosphoribosylamino)uracil reductase)	PPA1752	CYT
PFREUD_21660	rpiB3	Ribose-5-phosphate isomerase 3	PPA1624	CYT
PFREUD_21700		Hypothetical fusion protein	PPA1172	CYT
PFREUD_21760	moaE2, moaE	Molybdopterin synthase MoaE, Molybdenum cofactor biosynthesis protein E 2	PPA0500	CYT
PFREUD_21810	moaB (mog)	Molybdenum cofactor biosynthesis protein	PPA0513	CYT

PFREUD_21980		Two component transcriptional regulator, LuxR family	PPA0055	CYT
PFREUD_22110		Hypothetical protein	PPA0040	CYT
PFREUD_22160		Metal binding protein	PPA0443	CYT
PFREUD_22330		Aldose 1-epimerase	PPA2052	CYT
PFREUD_22340	uvrA4	Excinuclease ATPase subunit UvrA	PPA0877	CYT
PFREUD_22360	tkt	Transketolase	PPA2318	CYT
PFREUD_22370	araB	L-ribulokinase	PPA2319	CYT
PFREUD_22380	araM	L-arabinose utilization protein	PPA2320	CYT
PFREUD_22400	araB, xylB	Carbohydrate kinase	PPA2321	CYT
PFREUD_22410	araL	L-arabinose operon protein, hydrolase	PPA2322	CYT
PFREUD_22430	rpiB2	Ribose-5-phosphate isomerase 2	PPA2324	CYT
PFREUD_22450	deoR3	Putative regulatory protein, DeoR family	PPA2326	CYT
PFREUD_22470	dhaK/dhaL	Dihydroxyacetone kinase	PPA0622	CYT
PFREUD_22780	hsp20 1	Heat shock protein 20 1 (20 kDa chaperone 1)	PPA0737	CYT
PFREUD_22840		hexulose-6-phosphate isomerase	PPA0881	CYT
PFREUD_22860	pdxA2	4-hydroxythreonine-4-phosphate dehydrogenase	PPA0296	CYT
PFREUD_22890	nadE	Glutamine-dependent NAD(+) synthetase	PPA2266	CYT
PFREUD_22910		polar amino acid ABC transporter, ATP binding component	PPA0600	CYT
PFREUD_22960	pncA	pyrazinamidase/nicotinamidase	PPA0993	CYT
PFREUD_23000		Putative long-chain-fatty-acid--CoA ligase/synthetase	PPA2234	CYT
PFREUD_23010	nfo	endonuclease IV (deoxyribonuclease IV (phage-T4- induced))	PPA2235	CYT
PFREUD_23130	iolH	IolH (Myo-inositol catabolism IolH protein) (isomerase:epimerase)	PPA0465	CYT
PFREUD_23150	pf667	Carboxylic ester hydrolase	PPA0594	CYT
PFREUD_23160	ugpQ2	Glycerophosphoryl diester phosphodiesterase	PPA1839	CYT
PFREUD_23190		sugar (xylose) phosphate isomerase/epimerase	PPA0464	CYT
PFREUD_23200		Inositol 2-dehydrogenase	PPA0463	CYT
PFREUD_23210	lacI4	Transcriptional regulator, LacI family (HTH-type transcriptional regulator degA)	PPA0462	CYT
PFREUD_23300	proV	ABC-type glycine betaine transport, ATP-binding protein	PPA0407	CYT
PFREUD_23440	rplI	50S ribosomal protein L9	PPA2227	CYT
PFREUD_23450	rpsR	30S ribosomal protein S18	PPA2228	CYT
PFREUD_23460	ssb	Single-strand binding protein	PPA2229	CYT
PFREUD_23470	rpsF	30S ribosomal protein S6	PPA2230	CYT
PFREUD_23520		Hypothetical protein	PPA2231	CYT
PFREUD_23650		Hypothetical protein	PPA0125	CYT
PFREUD_23740	aglA	Alpha-1,4-glucosidase	PPA1630	CYT
PFREUD_23780	acs	Fatty-acyl-CoA synthase	PPA1724	CYT
PFREUD_23800	katA	Catalase	PPA0097	CYT
PFREUD_23890	fba2	Fructose-bisphosphate aldolase class I	PPA2024	CYT
PFREUD_23910	gltA1	Citrate synthase	PPA2272	CYT
PFREUD_23920	yghZ	Oxidoreductase	PPA0994	CYT
PFREUD_24000	fepC1	ABC transporter, iron transport ATPase protein	PPA0658	CYT
PFREUD_24040	pcnB	tRNA nucleotidyltransferase PcnB	PPA2301	CYT
PFREUD_24080	gpm1	phosphoglycerate mutase/fructose-2,6- bisphosphatase	PPA0809	CYT
PFREUD_24100	trxB	Thioredoxin reductase	PPA2309	CYT
PFREUD_24120	osmC	OsmC protein	PPA2313	CYT
PFREUD_24140	parB	chromosome partitioning protein	PPA2341	CYT
PFREUD_24220		Hypothetical protein	PPA2353	CYT
PFREUD_00300	iolT2	iolT2 (myo-inositol transporter iolT2)	PPA0467	MEMBRANE
PFREUD_00380	cycA2	D-serine/D-alanine/glycine transporter	PPA2269	MEMBRANE
PFREUD_00960	hflC1	Stomatin/prohibitin homolog	PPA1973	MEMBRANE
PFREUD_01640		two component sensor kinase	PPA2027	MEMBRANE
PFREUD_01730	cydB	Cytochrome d ubiquinol oxidase, subunit II	PPA0175	MEMBRANE
PFREUD_02100		ABC-type transporter	PPA0179	MEMBRANE
PFREUD_02230	whiP	Hypothetical transmembrane protein	PPA0182	MEMBRANE
PFREUD_02360	galP	Sodium:galactoside symporter	PPA2331	MEMBRANE
PFREUD_02650		sugar transporter	PPA1822	MEMBRANE
PFREUD_03190	matE	Multi antimicrobial extrusion protein MatE	PPA2259	MEMBRANE
PFREUD_03280		ABC-type transport systems, periplasmic component	PPA1758	MEMBRANE
PFREUD_03300		ABC transporter, permease protein	PPA1760	MEMBRANE
PFREUD_03400	aroP	Aromatic amino acid transport protein aroP	PPA1069	MEMBRANE
PFREUD_03410		Cation-efflux transport protein	PPA1228	MEMBRANE
PFREUD_03570	PPA1043	Anaerobic C4-dicarboxylate transporter	PPA1043	MEMBRANE
PFREUD_03780		Hypothetical membrane protein	PPA0093	MEMBRANE
PFREUD_03890	mviN	Conserved membrane protein, MviN-like protein	PPA2147	MEMBRANE
PFREUD_03930		aminoacid permease	PPA0177	MEMBRANE
PFREUD_03990		transport protein	PPA2034	MEMBRANE
PFREUD_04230		Hypothetical protein	PPA2140	MEMBRANE
PFREUD_04490	PPA2042	Putative low-affinity phosphate transport protein	PPA2042	MEMBRANE
PFREUD_04500		Hypothetical protein	PPA2041	MEMBRANE
PFREUD_04570	cbiQ2	Cobalt transport protein CbiQ	PPA0433	MEMBRANE
PFREUD_04590	cbiM	Cobalt transport protein CbiM	PPA0430	MEMBRANE
PFREUD_04610	gnuT	Gluconate permease (transmembrane)	PPA0738	MEMBRANE
PFREUD_05020	czcD	Co/Zn/Cd cation transporter CzcD	PPA1951	MEMBRANE
PFREUD_05080	menA	1,4-dihydroxy-2-naphthoateoctaprenyltransferase	PPA1945	MEMBRANE
PFREUD_05120		Hypothetical membrane protein	PPA1941	MEMBRANE
PFREUD_05160	nuoA	NADH-quinone oxidoreductase chain	PPA1936	MEMBRANE
PFREUD_05230	nuoH	NADH-quinone oxidoreductase subunit H (NADH dehydrogenase I subunit H)	PPA1929	MEMBRANE
PFREUD_05250	nuoJ	NADH-quinone oxidoreductase chain J (NADH dehydrogenase I, chain J)	PPA1926	MEMBRANE
PFREUD_05260	nuoK	NADH dehydrogenase I chain K	PPA1925	MEMBRANE
PFREUD_05270	nuoL	NADH dehydrogenase (quinone)	PPA1924	MEMBRANE
PFREUD_05280	nuoM	NADH dehydrogenase I chain M	PPA1923	MEMBRANE
PFREUD_05290	nuoN	NADH dehydrogenase I chain N	PPA1922	MEMBRANE
PFREUD_05360		Hypothetical membrane protein	PPA1900	MEMBRANE
PFREUD_05950	oppC	ABC transporter, permease protein OppC	PPA2065	MEMBRANE
PFREUD_05970	secY	Preprotein translocase SecY subunit	PPA1835	MEMBRANE
PFREUD_06130		ABC transporter	PPA1816	MEMBRANE
PFREUD_06140	pf2694	ABC-type transporter, permease components	PPA1814	MEMBRANE
PFREUD_06160		ABC transporter-associated permease	PPA1812	MEMBRANE
PFREUD_06710	pspC	Possible stress-response transcriptional regulator protein PspC	PPA1763	MEMBRANE
PFREUD_06740		Hypothetical protein	PPA1762	MEMBRANE
PFREUD_07180		Hypothetical protein	PPA0667	MEMBRANE
PFREUD_07310	purE	Phosphoribosylaminoimidazole carboxylase catalytic subunit	PPA1701	MEMBRANE
PFREUD_07430		Cation transport protein	PPA1687	MEMBRANE
PFREUD_07900		Membrane protein	PPA1641	MEMBRANE

PFREUD_07920		Hypothetical membrane protein	PPA1640	MEMBRANE
PFREUD_07940	PPA1638	Putative ABC transporter	PPA1638	MEMBRANE
PFREUD_08220		Hypothetical membrane protein	PPA1574	MEMBRANE
PFREUD_08860		Hypothetical secreted protein	PPA0938	MEMBRANE
PFREUD_09240	sdhC1	Succinate dehydrogenase subunit C	PPA0950	MEMBRANE
PFREUD_09290		ABC-type transport system, permease component	PPA0956	MEMBRANE
PFREUD_09510		Amino acid permease. Membran protein	PPA0969	MEMBRANE
PFREUD_10050		Permease protein of oligopeptide ABC transporter	PPA1275	MEMBRANE
PFREUD_10270		Hypothetical protein	PPA1260	MEMBRANE
PFREUD_10390	tagO	Undecaprenyl-phosphate alpha-N- acetylglucosaminyltransferase	PPA1248	MEMBRANE
PFREUD_10430	atpB	ATP synthase A chain (ATPase protein 6) (F1F0- ATPase subunit a)	PPA1245	MEMBRANE
PFREUD_10440	atpE	ATP synthase C chain (F1F0-ATPase subunit c)	PPA1244	MEMBRANE
PFREUD_10450	atpF	ATP synthase B chain (F0F1 ATP synthase subunit B)	PPA1243	MEMBRANE
PFREUD_10510		Hypothetical secreted protein	PPA1237	MEMBRANE
PFREUD_10730		Hypothetical membrane anchored protein	PPA1119	MEMBRANE
PFREUD_11200		Permeases of the drug/metabolite transporter (DMT) superfamily	PPA0923	MEMBRANE
PFREUD_11670	secF	Protein-export membrane protein secF	PPA1163	MEMBRANE
PFREUD_11870	lgt	Prolipoprotein diacylglyceryl transferase	PPA1133	MEMBRANE
PFREUD_12240	cobS	Cobalamin 5 -phosphate synthase (adenosylcobinamide-GDP ribazoletransferase)	PPA0442	MEMBRANE
PFREUD_12370		Small basic protein (membrane)	PPA1081	MEMBRANE
PFREUD_12390	pgs1	CDP-diacylglycerol--glycerol-3-phosphate 3- phosphatidyl- transferase	PPA1079	MEMBRANE
PFREUD_12420	pgs2	CDP-diacylglycerol--glycerol-3-phosphate 3- phosphatidyl-transferase	PPA1078	MEMBRANE
PFREUD_13190		Phosphatidylglycerophosphate synthase	PPA1007	MEMBRANE
PFREUD_13320		Membrane protease	PPA1365	MEMBRANE
PFREUD_13510	tatC	Sec-independent protein translocase protein TatC homolog	PPA1378	MEMBRANE
PFREUD_13690		Cobalt permease	PPA1398	MEMBRANE
PFREUD_13720		BioY family protein. Membrane protein	PPA1400	MEMBRANE
PFREUD_14090		Hypothetical protein	PPA1416	MEMBRANE
PFREUD_14200	uppP (BacA)	Undecaprenyl-diphosphatase (Undecaprenyl pyrophosphate phosphatase) (Bacitracin resistance protein)	PPA2224	MEMBRANE
PFREUD_14320	sdhC2	Succinate dehydrogenase cytochrome B-558 subunit	PPA1439	MEMBRANE
PFREUD_14430		Hypothetical membrane protein	PPA1448	MEMBRANE
PFREUD_14610	glpF	Glycerol uptake facilitator protein,	PPA2305	MEMBRANE
PFREUD_14740	cdsA	Phosphatidate cytidylyltransferase	PPA1517	MEMBRANE
PFREUD_14830		Transporter, sodium/bile acid symporter family	PPA1527	MEMBRANE
PFREUD_15290	terC	Membrane protein TerC	PPA0804	MEMBRANE
PFREUD_15440		Hypothetical protein	PPA0764	MEMBRANE
PFREUD_15510	ftsW1	Cell division protein FtsW	PPA0757	MEMBRANE
PFREUD_15530	mraY	Phospho-N-acetylmuramoyl-pentapeptide- transferase (UDP- MurNAc-pentapeptide phosphotransferase)	PPA0755	MEMBRANE
PFREUD_15700		Hypothetical transmembrane protein	PPA0728	MEMBRANE
PFREUD_15810		Hypothetical protein	PPA0715	MEMBRANE
PFREUD_15950		Hypothetical protein	PPA0683	MEMBRANE
PFREUD_16130		Hypothetical protein	PPA0646	MEMBRANE
PFREUD_16220		Chloride channel	PPA2253	MEMBRANE
PFREUD_16340	dcuA	C4-dicarboxylate transporter	PPA0095	MEMBRANE
PFREUD_16350	pimH	Integral membrane efflux protein	PPA1946	MEMBRANE
PFREUD_16500	cstA	Carbon starvation protein	PPA0660	MEMBRANE
PFREUD_17000		Hypothetical membrane protein	PPA0565	MEMBRANE
PFREUD_17210		Hypothetical membrane protein	PPA0549	MEMBRANE
PFREUD_17510		dolichyl-phosphate-mannose-protein mannosyltransferase (Glycosyl transferase, family 39)	PPA0523	MEMBRANE
PFREUD_17530	rutG	Pyrimidine permease RutG (Pyrimidine utilization protein G)	PPA0522	MEMBRANE
PFREUD_17580		Hypothetical membrane protein	PPA0770	MEMBRANE
PFREUD_17590		Hypothetical protein	PPA0771	MEMBRANE
PFREUD_17660	proP	Proline/betaine transporter	PPA0634	MEMBRANE
PFREUD_17670		Permease	PPA0285	MEMBRANE
PFREUD_17980		Hypothetical membrane protein	PPA0496	MEMBRANE
PFREUD_18180		permease	PPA0480	MEMBRANE
PFREUD_18660	lldP	L-lactate permease	PPA0166	MEMBRANE
PFREUD_18720		Amino acid permease. membrane protein	PPA0368	MEMBRANE
PFREUD_19180	dedD	Conserved membrane protein (DedA family)	PPA2014	MEMBRANE
PFREUD_19240		Oligopeptide transporter, OPT family protein.	PPA0554	MEMBRANE
PFREUD_19320		Hypothetical membrane protein	PPA0931	MEMBRANE
PFREUD_19570		Forkhead-associated protein	PPA0188	MEMBRANE
PFREUD_19590	ftsW2	Cell division protein	PPA0186	MEMBRANE
PFREUD_19650	feoB	Ferrous iron transport protein B	PPA1677	MEMBRANE
PFREUD_19680		Na+/H+ antiporter	PPA2179	MEMBRANE
PFREUD_20120		Hypothetical membrane protein	PPA0317	MEMBRANE
PFREUD_20180		ABC transporter	PPA0156	MEMBRANE
PFREUD_20400	corA	CorA, Mg2+ and Co2+ transporters	PPA1716	MEMBRANE
PFREUD_20460	cycA1	D-serine/D-alanine/glycine transporter	PPA1643	MEMBRANE
PFREUD_20590	dmsC	Anaerobic dimethyl sulfoxide reductase chain C	PPA0515	MEMBRANE
PFREUD_20700		Hypothetical membrane protein	PPA0231	MEMBRANE
PFREUD_20740	NhaA	sodium:proton antiporter	PPA0227	MEMBRANE
PFREUD_20780		Cation-transporting ATPase	PPA2240	MEMBRANE
PFREUD_20910	slgT	Na+/galactose cotransporter / Sodium/glucose cotransporter	PPA2193	MEMBRANE
PFREUD_20950	mntH	Mn2+ and Fe2+ transporter	PPA2026	MEMBRANE
PFREUD_21530	pntB	NADH dehydrogenase	PPA0626	MEMBRANE
PFREUD_21540	pntA	NAD(P)(+) transhydrogenase (AB-specific).	PPA0625	MEMBRANE
PFREUD_22020		Major facilitator super family MSF1	PPA0048	MEMBRANE
PFREUD_22420		Hypothetical membrane protein	PPA2323	MEMBRANE
PFREUD_22800	kup	Potassium transport system protein	PPA2187	MEMBRANE
PFREUD_23170		Membrane protein, Transporter, MFS superfamily	PPA1837	MEMBRANE
PFREUD_23310	proW	ABC-type glycine betaine transport protein	PPA0406	MEMBRANE
PFREUD_23320	proZ	PROZ-like protein (ABC-type glycine betaine transport, permease protein)	PPA0405	MEMBRANE
PFREUD_23400		Binding-protein-dependent transport systems inner membrane component	PPA0399	MEMBRANE
PFREUD_23410		Binding-protein-dependent transport systems inner membrane component	PPA0057	MEMBRANE
PFREUD_23620	pigV	GPI mannosyltransferase 2	PPA0128	MEMBRANE
PFREUD_24010	fepD	iron ABC transporter, permease protein	PPA0657	MEMBRANE
PFREUD_24190	oxaA	Conserved membrane protein	PPA2350	MEMBRANE
PFREUD_00180		Hypothetical protein	PPA1807	PSE
PFREUD_00350		ABC transporter	PPA0099	PSE
PFREUD_00810		Sensor protein, ATPase-like:Histidine kinase	PPA2113	PSE
PFREUD_00980	norB	Nitric-oxide reductase subunit B (nitric-oxide reductase)	PPA1975	PSE
PFREUD_01090	gltA2	Citrate synthase	PPA1423	PSE
PFREUD_01490		membrane protein without function	PPA2195	PSE

PFREUD_01610		ABC transporter permease	PPA2031	PSE
PFREUD_01720	cydA	Cytochrome d ubiquinol oxidase subunit I	PPA0176	PSE
PFREUD_02090		ABC transporter, transmembrane region	PPA0178	PSE
PFREUD_05510	secE	SecE/Sec61-gamma subunit of protein translocation complex	PPA1892	PSE
PFREUD_05930	bopA	solute binding protein of the ABC transport system	PPA1840	PSE
PFREUD_06630	biaA	ABC transport system component	PPA0017	PSE
PFREUD_06790		Phospholipase D/Transphosphatidylase	PPA1761	PSE
PFREUD_06900		Hypothetical protein	PPA1736	PSE
PFREUD_07360		Glycosyltransferase	PPA1693	PSE
PFREUD_07460		Hypothetical protein	PPA1684	PSE
PFREUD_08330	dsbG	DSBA oxidoreductase	PPA1567	PSE
PFREUD_08340		Hypothetical membrane protein	PPA1566	PSE
PFREUD_08650	comEC	ComE operon protein 3	PPA0895	PSE
PFREUD_09360	chlD	ChlD, Mg-chelatase subunit ChlD	PPA0976	PSE
PFREUD_09370		Von Willebrand factor, type A	PPA0977	PSE
PFREUD_09430	fabH	3-oxoacyl-(Acyl-carrier-protein) synthase III	PPA0983	PSE
PFREUD_09600	ftsX	Cell division protein	PPA1352	PSE
PFREUD_09640		Hypothetical transmembrane protein	PPA1344	PSE
PFREUD_09940		Secreted protease with a PDZ domain	PPA1310	PSE
PFREUD_10030		ABC transporter, substrate binding protein	PPA1277	PSE
PFREUD_10040		ABC transporter permease protein	PPA1276	PSE
PFREUD_10970		Hypothetical protein	PPA1221	PSE
PFREUD_11540		Aminodeoxychorismate lyase	PPA1180	PSE
PFREUD_11680	secD	Protein-export membrane protein secD	PPA1162	PSE
PFREUD_12270		transporter	PPA1203	PSE
PFREUD_12360		Hypothetical protein	PPA1082	PSE
PFREUD_12380		Hypothetical protein	PPA1080	PSE
PFREUD_12690	ydaO	Amino acid permease	PPA1059	PSE
PFREUD_13110		Metal dependent phosphohydrolase	PPA1014	PSE
PFREUD_13210	ftsK	DNA translocase FtsK	PPA1356	PSE
PFREUD_14120		FtsH	PPA0260	PSE
PFREUD_14280	lepB	Signal peptidase I	PPA1434	PSE
PFREUD_14310	sdhA3	Succinate dehydrogenase flavoprotein subunit	PPA1438	PSE
PFREUD_14550		Hypothetical protein	PPA1469	PSE
PFREUD_14680		Hypothetical protein	PPA1496	PSE
PFREUD_15410	lspA	Lipoprotein signal peptidase, signal peptidase II	PPA0767	PSE
PFREUD_15480	ftsQ	Cell division protein FtsQ	PPA0760	PSE
PFREUD_15780		Hypothetical protein	PPA0717	PSE
PFREUD_16040		Hypothetical protein	PPA0663	PSE
PFREUD_16450		ABC transporter glycine betaine ?	PPA0404	PSE
PFREUD_16480		major facilitator superfamily	PPA1726	PSE
PFREUD_18030		Hypothetical protein	PPA0492	PSE
PFREUD_18790	senX	Sensor-like histidine kinase	PPA0360	PSE
PFREUD_19390		Metallophosphoesterase	PPA2150	PSE
PFREUD_19400	ponA	Penicillin-binding protein (Transglycosylase/transpeptidase)	PPA2149	PSE
PFREUD_19580	pf456	Hypothetical protein	PPA0187	PSE
PFREUD_19610	pknB	Serine/threonine protein kinase	PPA0184	PSE
PFREUD_19960	pstC	ABC transporter, permease protein	PPA0340	PSE
PFREUD_20650		Hypothetical protein	PPA0235	PSE
PFREUD_21970		two component sensor kinase	PPA0068	PSE
PFREUD_22080		Hypothetical membrane protein	PPA0043	PSE
PFREUD_22490		Membrane permease (sugar transporter)	PPA0295	PSE
PFREUD_23610		Alpha-amylase	PPA0592	PSE
PFREUD_23640	mrc/ponA	penicillin-binding protein (peptidoglycan glycosyltransferase)	PPA0126	PSE
PFREUD_23840		Chloride channel protein	PPA0080	PSE
PFREUD_24060		Hypothetical protein	PPA2302	PSE
PFREUD_02530		Hypothetical secreted protein	PPA2130	SECRETED
PFREUD_02660		Metallophosphoesterase	PPA1498	SECRETED
PFREUD_03070		Hypothetical protein	PPA0444	SECRETED
PFREUD_06750	feuS	iron ABC transport system, solute-binding protein precursor	PPA0334	SECRETED
PFREUD_09270	dus	TIM-barrel enzyme, dihydrouridine synthase	PPA0954	SECRETED
PFREUD_12220	cbiA	Cobyrinic acid A,C-diamide synthase, CbiA	PPA0438	SECRETED
PFREUD_14400	ftsY	Signal recognition particle receptor	PPA1447	SECRETED
PFREUD_16260		Hypothetical secreted protein	PPA0629	SECRETED
PFREUD_16950	rmuC	DNA recombination protein	PPA0573	SECRETED
PFREUD_17340		Hypothetical protein	PPA0542	SECRETED
PFREUD_19470	ptsI	PTS enzyme I	PPA0351	SECRETED
PFREUD_19600	pbpA	Penicillin-binding protein	PPA0185	SECRETED
PFREUD_22120		Hypothetical membrane protein	PPA0039	SECRETED
