# Supplementary material for: The Complete Genome of Propionibacterium freudenreichii CIRM-BIA1T, a Hardy Actinobacterium with Food and Probiotic Applications
Source: PLoS One. 2010 Jul 23;5(7):e11748. doi: 10.1371/journal.pone.0011748 (PMC2909200; doi:10.1371/journal.pone.0011748)
Supplement: Table S4 — Propionibacterium freudenreichii carbon substrate degradation. (0.03 MB PDF) [file pone.0011748.s004.pdf]

Carbon substrates	*P. freudenreichii* (1)	*P. freudenreichii* (2)	CIP103027^T
glucose	+	+	+
galactose	+	+	+
D-mannose	+	+	+
D-fructose	+	d+	+
lactose	+ or – (3)	d+	+
glycerol	+	+	+
erythritol	+	+	+
adonitol	d+	+	+
Lactic acid	+	+	+
Gluconic acid	nr	d-	+
Esculine hydrolysis	+	+	-
inositol	d+	+	-
L-arabinose	+	d+	-
ribose	d+	d-	-
melibiose	-	d-	-
D-raffinose	-	d-	+
Saccharose	-	d-	-
L-arabitol	nr	d-	-
Xylitol	nr	d-	-

+, positive reaction in 90-100% of strains; -, negative reaction in 90-100% of strains; d+, positive reaction in 40-90% of strains; d-, negative reaction in 40-100% of strains;

(1) Reference [35]

(2) This study: results of tests in Api 50CH strips for 150 *P. freudenreichii* strains

(3) Depending on the subspecies *freudenreichii* or *shermanii*

nr: not reported
