# Supplementary material for: The Complete Genome of Propionibacterium freudenreichii CIRM-BIA1T, a Hardy Actinobacterium with Food and Probiotic Applications
Source: PLoS One. 2010 Jul 23;5(7):e11748. doi: 10.1371/journal.pone.0011748 (PMC2909200; doi:10.1371/journal.pone.0011748)
Supplement: Table S5 — Results of test of vitamin requirements by P. freudenreichii CIRM-BIA1. (0.04 MB PDF) [file pone.0011748.s005.pdf]

Table S5 : Results of test of vitamin requirements by *P. freudenreichii* CIRM-BIA1[T]

Atmosphere	Mediuma	maximal OD_{650}	% OD_{ctl} [a]	Time (d)	CFU/mL[b]
Anaerobic	Control (9 vitamins) [c]	1.2	100	6.8	8.7
	without vit. B6	1.1	95	6.8	8.8
	without vit. PP	1.1	94	6.8	8.8
	without p-aminobenzoic acid and vit. B9	1.1	97	6.8	8.8
	without vit. B2	1.1	95	8.8	8.9
	without vit. B12	1.0	84	9.5	8.6
	without vit. B1	0.6	36	7.1	nd [d]
	without vit. B5	0.2	6	> 12	nd
	without vit. H	0.2	6	> 12	nd
Semi-anaerobic	Control (9 vitamins)	1.5	100	9.0	8.7
	without vit. B6	1.4	95	8.3	8.7
	without vit. PP	1.5	103	8.3	8.8
	without p-aminobenzoic acid and vit. B9	1.3	92	9.5	8.7
	without vit. B2	1.4	95	9.3	8.8
	without vit. B12	1.4	98	8.8	8.6
	without vit. B1	1.1	19	9.0	nd
	without vit. B5	0.1	2	> 12	nd
	without vit. H	0.1	2	> 12	nd

[a] % OD_{ctl} , OD_{650} of the medium tested / OD_{650} in the control medium x 100

[b] CFU, colony-forming units

[c] the control medium contained 9 vitamins: pyridoxal phosphate (B6), nicotinic acid (PP), pantothenate(B5), thiamine(B1), riboflavin(B2), p-aminobenzoic acid, folic acid (B9), biotine (H), and vitamin B12)

[d] nd, not determined
