# Supplementary material for: The Complete Genome of Propionibacterium freudenreichii CIRM-BIA1T, a Hardy Actinobacterium with Food and Probiotic Applications
Source: PLoS One. 2010 Jul 23;5(7):e11748. doi: 10.1371/journal.pone.0011748 (PMC2909200; doi:10.1371/journal.pone.0011748)
Supplement: Table S6 — List of pyrophosphate and polyphosphate enzymes. (0.03 MB PDF) [file pone.0011748.s006.pdf]

locus_tag	product	EC number	function	gene name
PFREUD_02720	NUDIX hydrolase		4.1 Adaptation to atypical conditions	
PFREUD_07140	NUDIX hydrolase		4.1 Adaptation to atypical conditions	
PFREUD_09230	NUDIX hydrolase		4.1 Adaptation to atypical conditions	
PFREUD_10620	NUDIX hydrolase		4.1 Adaptation to atypical conditions	
PFREUD_17200	NUDIX hydrolase		4.1 Adaptation to atypical conditions	
PFREUD_18300	NUDIX hydrolase		4.1 Adaptation to atypical conditions	
PFREUD_19450	NUDIX hydrolase		4.1 Adaptation to atypical conditions	*pfp (pfk)*
PFREUD_19940	NUDIX hydrolase		4.1 Adaptation to atypical conditions	*cobU*
PFREUD_09830	NUDIX hydrolase		4.1 Adaptation to atypical conditions	*phk*
PFREUD_12230	CobU Bifunctional cobalamin biosynthesis pyrophosphate enzyme	2.7.7.62	2.5 Metabolism of coenzymes and prosthetic groups	*ppgK*
PFREUD_15680	Geranylgeranyl pyrophosphate synthase	2.5.1.29	2.5 Metabolism of coenzyme and prosthetic group	
PFREUD_17010	Undecaprenyl pyrophosphate synthase (di-trans,poly-cis-decaprenylcistransferase)	2.5.1.31	2.4 Metabolism of lipids	
PFREUD_14200	Undecaprenyl-diphosphatase (Undecaprenyl pyrophosphate phosphatase) (Bacitracin resistance protein)	3.6.1.27	1.1 Cell wall	
PFREUD_12930	Phosphoketolase pyrophosphate	4.1.2.9	2.1.2 Main glycolytic pathways	*ppnK*
PFREUD_02600	Polyphosphate glucokinase	2.7.1.63	2.1.2 Main glycolytic pathways	*uppP (BacA)*
PFREUD_19930	Polyphosphate kinase	2.7.4.1	2.6 Metabolism of phosphate	
PFREUD_13670	Probable inorganic polyphosphate/ATP-NAD kinase (Poly(P)/ATP NAD kinase)	2.7.1.23	2.6 Metabolism of phosphate	*uppS*
PFREUD_12040	pyrophosphate phosphofructokinase	2.7.1.90	2.1 Metabolism of carbohydrates and related molecules	
PFREUD_22260	ribose-phosphate pyrophosphokinase (RPPK) (Phosphoribosyl pyrophosphate synthetase) (P-Rib-PP synthetase) (PRPP synthetase)	2.7.6.1	2.3 Metabolism of nucleotides and nucleic acids	
PFREUD_05340	Thiamine pyrophosphate (TPP family)		2.6 Metabolism of phosphate	
PFREUD_04430	Thiamine pyrophosphate enzyme		2.6 Metabolism of phosphate	*ppk*
